# Retargeting Gram-Positive-Only Adarotene-Derived Antibacterials to Broad-Spectrum Antibiotics

**DOI:** 10.3390/antibiotics14090956

**Published:** 2025-09-21

**Authors:** Salvatore Princiotto, Luigi Cutarella, Alessandra Fortuna, Marta Mellini, Bruno Casciaro, Maria Rosa Loffredo, Alvaro G. Temprano, Floriana Cappiello, Livia Leoni, Maria Luisa Mangoni, Mattia Mori, Loana Musso, Francesca Sacchi, Cecilia Pinna, Giordano Rampioni, Sabrina Dallavalle, Claudio Pisano

**Affiliations:** 1 Department of Food, Environmental and Nutritional Sciences (DeFENS), University of Milan, via Celoria 2, 20133 Milan, Italy; salvatore.princiotto@unimi.it (S.P.); loana.musso@unimi.it (L.M.); francesca.sacchi@unimi.it (F.S.); cecilia.pinna@unimi.it (C.P.); 2 Department of Biotechnology, Chemistry and Pharmacy, University of Siena, via Aldo Moro 2, 53100 Siena, Italy; l.cutarella@student.unisi.it (L.C.); alvarogacho@usal.es (A.G.T.); mattia.mori@unisi.it (M.M.); 3 Department of Science, University Roma Tre, Viale G. Marconi 446, 00146 Rome, Italy; alessandra.fortuna@uniroma3.it (A.F.); marta.mellini@uniroma3.it (M.M.); livia.leoni@uniroma3.it (L.L.); 4 Laboratory affiliated to Pasteur Italia-Fondazione Cenci Bolognetti, Department of Biochemical Sciences, Sapienza University of Rome, 00185 Rome, Italy; bruno.casciaro@uniroma1.it (B.C.); mariarosa.loffredo@uniroma1.it (M.R.L.); floriana.cappiello@uniroma1.it (F.C.); marialuisa.mangoni@uniroma1.it (M.L.M.); 5 Experimental Hepatology and Drug Targeting (HEVEPHARM), Institute of Biomedical Research of Salamanca (IBSAL), University of Salamanca, 37007 Salamanca, Spain; 6 Center for the Study of Liver and Gastrointestinal Diseases (CIBEREHD), Carlos III National Institute of Health, 28029 Madrid, Spain; 7 IRCCS Fondazione Santa Lucia, Via Ardeatina 306/354, 00179 Rome, Italy; 8 Special Products Line, 03012 Anagni, Italy; c.pisano@specialspa.it

**Keywords:** antibacterial, adarotene, broad-spectrum, outer membrane, multidrug-resistance, Gram-positive, Gram-negative

## Abstract

**Background**: Bacterial resistance to antibiotics continues to rise globally, posing a significant public health challenge and incurring substantial social and economic burdens. In response, the World Health Organization (WHO) has published a list of priority pathogens for which effective treatment options are critically limited. Several antibiotics are categorized as Gram-positive-only (GPO) agents due to their lack of activity against Gram-negative species. Although these compounds often target conserved bacterial processes, their limited spectrum is largely attributed to poor penetration of the Gram-negative outer membrane (OM). **Results**: In this study, we designed and synthesized a series of adarotene-derived compounds to evaluate the impact of introducing positively charged groups on their interaction with the Gram-negative OM. One of the newly synthesized derivatives, **SPL 207**, displayed minimum inhibitory concentration (MIC) values ranging from 8 to 64 µM against a panel of Gram-positive and Gram-negative bacteria. The ability of **SPL207** to disrupt outer and inner membrane permeability was evaluated using fluorescence assays and confocal microscopy, revealing that the compound compromises membrane integrity across all tested Gram-negative bacteria. Strong synergistic activity was observed in combination with colistin against three *P. aeruginosa* colistin-resistant strains. Atomistic details of membrane interference were elucidated by molecular dynamics (MD) simulations, with **SPL207** clearly acting as a membrane destabilizer by enhancing Ca^2+^ ions diffusion and lipids destabilization. **Conclusions**: Although the observed MIC values remain above clinically acceptable thresholds, these findings provide a promising proof of concept. The further structural optimization of adarotene derivatives may yield novel broad-spectrum agents with improved antimicrobial potency against MDR pathogens.

## 1. Introduction

The rising prevalence of multidrug-resistant (MDR) pathogens—and those resistant to nearly all available antibiotics—is contributing to a growing number of deaths from bacterial infections worldwide. In addition, this trend is inversely paralleled by a decline in the discovery and development of new antibiotics. It has been predicted that 10 million people worldwide will die per year from antibiotic-resistant infections by 2050 [1,2,3].

From an economic perspective, infections due to MDR pathogens lead to prolonged hospitalization and repeated antibiotic courses, causing estimated extra healthcare costs of about EUR 1.1 billion to the healthcare systems in EU/EEA countries [4]. These costs are set to rise dramatically if novel antimicrobials will not be identified. In the last two decades Gram-negative bacteria have emerged as particularly problematic pathogens. With the launch of the EU4Health 2021–2027 program, and in line with HORIZON 2020, the European Commission has confirmed the fight against communicable diseases in general and, more specifically, against antibiotic resistance as a critical priority. The World Health Organization issued a list of ‘Priority Pathogens’ for which new antibiotics are urgently needed. The vast majority of these pathogens are Gram-negative, including third-generation cephalosporin- and carbapenem-resistant *Escherichia coli* and *Klebsiella pneumoniae*, carbapenem-resistant *Acinetobacter baumannii* and *Pseudomonas aeruginosa*, and fluoroquinolone-resistant *Shigella* species and *Salmonella enterica* ser. Typhi [1,5].

The lack of Gram-negative antibacterial discovery could be traced to the unique structure of their outer membrane (OM), usually composed of lipopolysaccharides (LPS). LPS are made up of lipid A and a long, negatively charged, oligosaccharide. Lipid A molecules contain 4–7 acyl chains (predominantly saturated), attached to a phosphorylated disaccharide. The adjacent negative charges of the LPS are stabilized by divalent cations. These features allow LPS molecules to stack together very tightly, making passive diffusion extremely challenging for most small molecules [6,7]. As many compounds cannot readily passively diffuse across the OM, small molecules permeate the OM through porins and/or the self-promoted uptake (SPU) pathway. Porins are water-filled β-barrels lined interiorly with charged amino acids. They form a narrow channel allowing only certain molecules to rapidly diffuse (e.g., certain β-lactams and fluoroquinolones). Other small molecules can pass through the OM via the SPU pathway by displacing the divalent cations, temporarily destabilizing the LPS layer. Compounds that utilize SPU are typically polycationic, as for instance colistin [8]; however, some compounds likely enter through a combination of both porins and SPU pathways [6].

For the above reasons, many antibiotics are inactive against Gram-negative pathogens, hence they have been termed “Gram-positive-only” (GPO) antibiotics [6]. The spectrum of activity of GPO antibiotics can be extended to Gram-negative bacteria through the addition of chemical groups that facilitate the passage of the pharmacophore across the OM. So far, the unique marketed example of broad-spectrum antibiotic derived from a GPO antibiotic is cefiderocol. This is made of a cephalosporin pharmacophore conjugated to a catechol-type siderophore. During the infection, bacteria experience iron starvation and respond expressing siderophores. The iron-bound catechol moiety is recognized by specific bacterial receptors located on the OM, promoting cefiderocol uptake. Once in the periplasm, the cephalosporin moiety can bind to the penicillin binding protein and inhibit cell wall synthesis [9]. A similar strategy has been recently used for expanding the range of activity of aztreonam, a GPO β-lactam antibiotic [10].

In the past, extensive screening campaigns for new classes of antibiotics gave no relevant results for Gram-negative bacteria. The lack of cell permeability was recognized as a major problem, since no rationale processes existed to improve hit compounds. However, recent structure-activity relationship (SAR) studies have provided some cues useful to transform GPO into broad-spectrum antibiotics. According to these criteria, named the eNTRy rules, compounds are most likely to be accumulated in Gram-negative bacteria if they: (i) contain a non-sterically encumbered ionizable Nitrogen; (ii) have low Three dimensionality; (iii) are relatively Rigid; (iv) have some non-polar functionality [6]. In support of the validity of the eNTRy rules, promising results have been recently published about modifications of bakuchiol, a prenylated phenolic monoterpene with antibacterial activity only against Gram-positive bacteria. Bakuchiol derivatives with antibacterial activity against Gram-negative pathogens and low toxicity against eukaryotic cells were obtained by introducing an aliphatic tail containing up to 3 positively charged amine groups. This tail favors the crossing through the OM, allowing the bakuchiol derivatives to disrupt the OM and cause cell death [11].

**Figure 1:**
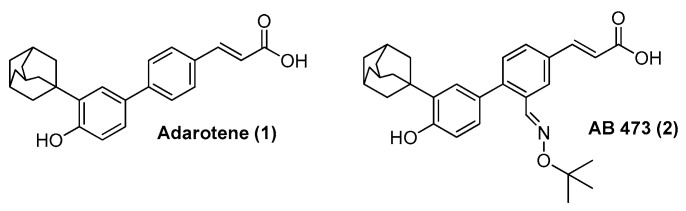
Structure of adarotene (**1**) and AB473 (**2**).

In 2018, the ability of atypical retinoids to penetrate and embed lipid bilayers was correlated with their bactericidal activity [12]. Starting from this evidence, we have recently synthesized novel adarotene-like molecules, belonging to the class of the atypical retinoids, endowed with significant antimicrobial activity against MDR strains of *Enterococcus faecalis* and *Staphylococcus aureus*. The most promising compound (**2**, AB473) showed a Minimal Inhibitory Concentration (MIC) of 4 µM (2 μg/mL) for all tested strains and very low cytotoxicity on human cells (Figure 1) [13,14] suggesting a potential high therapeutic index.

However, the great limitation of the adarotene derivatives obtained so far, including **AB473**, is their poor activity against Gram-negative bacteria (MIC > 128 μM against *E. coli*, *P. aeruginosa*, *A. baumannii*) [12,13]. To overcome this limitation, we designed new adarotene derivatives by following the above-mentioned eNTRy rules [6].

**Figure 2:**
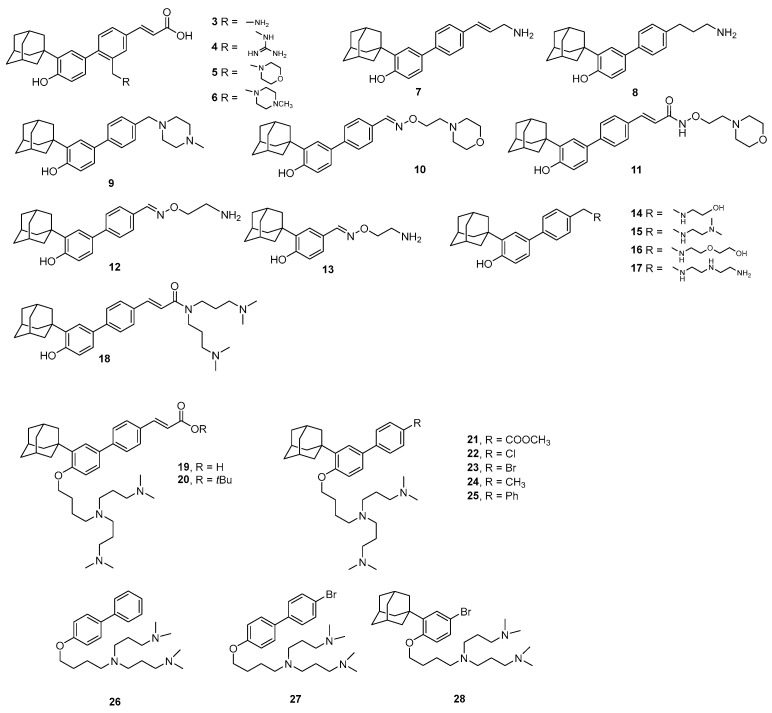
Analogs of adarotene with protonatable groups.

In particular, we aimed to investigate whether various positively charged nitrogen species could enhance the translocation of compounds across the Gram-negative OM. We hypothesized that the incorporated basic cationic moieties could favor the interaction between the derivatives and the negatively charged bacterial cell membrane. The subsequent insertion of the hydrophobic moiety into the phospholipid bilayer could lead to the destruction of the integrity of the bacterial membrane, leakage of intracellular content and bacterial cell death. Beyond enhanced OM crossing, amino moieties could provide other advantages, including higher solubility in aqueous environments (e.g., biological fluids) [15,16]. Thus, a diverse set of amine-containing adarotene-derived compounds were designed and synthesized (Figure 2). Additionally, a shift from simple amino groups to polycationic groups was implemented, with the aim of obtaining molecules capable of penetrating Gram-negative bacteria via the SPU pathway [6].

Bearing in mind that the amino groups need to be embedded within a compound possessing appropriate flexibility, the structural features of the linker (e.g., geometry, orientation, size, and chemical composition) connecting the charged group(s) to the core skeleton, as well as the position of attachment, were carefully modulated. The installation of the selected moieties was designed not only on the biphenyl skeleton, but also on the key functional groups of the parent compound (e.g., the carboxylic group and the phenolic OH), which will serve as sites of attachment of the chains by amide and ether bonds, respectively (Figure 2). To assess membrane integrity disruption, fluorescence assays and microscopy imaging evaluation were planned for the most promising compounds identified by their MIC profiles. A validated *P. aeruginosa* OM model was developed to enable in silico investigations of ligand penetration. Finally, considering that colistin is one of the last-resort treatments for *P. aeruginosa*, we aimed to evaluate whether the most promising compounds exhibit synergistic effects against colistin-resistant strains.

## 2. Results and Discussion

### 2.1. Chemistry

Adarotene analogs and derivatives were synthesized to explore their structural and biological properties. Given the critical role of protonatable nitrogen-containing side chains in enhancing anti-Gram-negative activity, all synthesized compounds incorporated (poly)aminic residues.

**Scheme 1:**
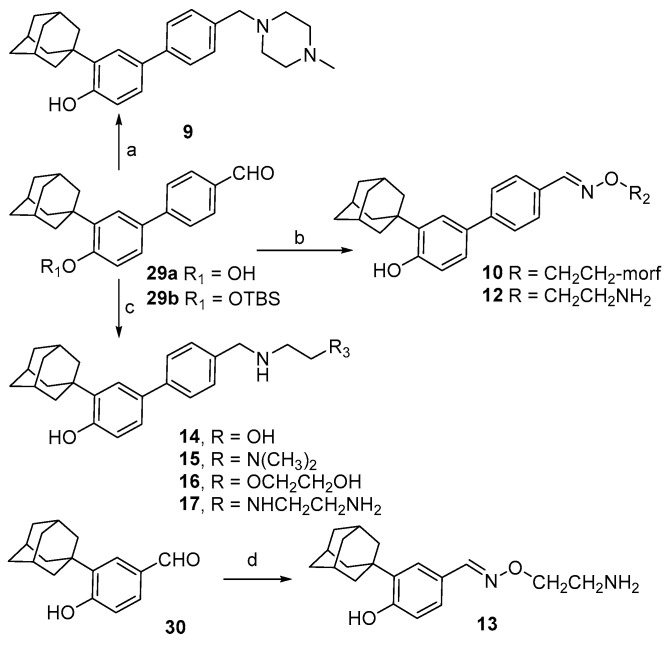
Reagents and conditions: (a) **29b**, 1-methylpiperazine, formic acid, BF_3_Et_2_O, CH_3_CN, 6 h, 85 °C, 38%; (b) for **10**: **29a**, *O*-(2-morpholinoethyl)hydroxylamine hydrochloride, pyridine, EtOH, 5 h, reflux, 67%; for **12**: **29a**, 2-(aminooxy)ethan-1-amine hydrochloride, pyridine, EtOH, refluxed for 5 h, 51%; (c) for **14**: i. **29a**, ethanolamine, MeOH, 0 °C then 18 h at rt, ii. NaBH_4,_ MeOH, 0 °C, 30 min, 97%; for **15**: i. **29a**, *N*^1^,*N*^1^-dimethylethane-1,2-diamine, MeOH, 0 °C then 18 h at rt, ii. NaBH_3_CN, MeOH, 0 °C, 40 min, 84%; for **16**: i. **29a**, 2-(2-aminoethoxy)ethan-1-ol, MeOH, 0 °C then 18 h at rt, ii. NaBH_3_CN, MeOH, 0 °C, 30 min, 54%; for **17**: i. **29a**, *N*^1^-(2-aminoethyl)ethane-1,2-diamine, 0 °C then 18 h rt, ii. NaBH_4_, MeOH, 0 °C, 30 min, 49%; (d) 2-(aminooxy)ethan-1-amine hydrochloride, pyridine, EtOH, 3 h, reflux, 37%.

Aldehydes **29a,b** [14,17] were used as starting material for the preparation of benzylamines **9** and **14**–**17**, as well as oximes **10** and **12** (Scheme 1). Reductive amination of **29a** with *N*-methyl piperazine in presence of formic acid and BF_3_Et_2_O afforded derivative **9**. Treatment with ethanolamine and *N*,*N*-dimethylethylenediamine gave **14** and **15**, respectively; longer and more flexible chains were installed by reductive amination using diethylenetriamine and its corresponding alcohol (compound **17** and **16**, respectively). Oximes **10** and **12** were prepared by treatment of the suitable aldehyde with the corresponding *O*-alkylated hydroxylamine in EtOH at reflux temperature. Similarly, aldehyde **30** furnished oxime **13** upon reaction with 2-(aminooxy)-ethan-1-amine hydrochloride.

**Scheme 2:**
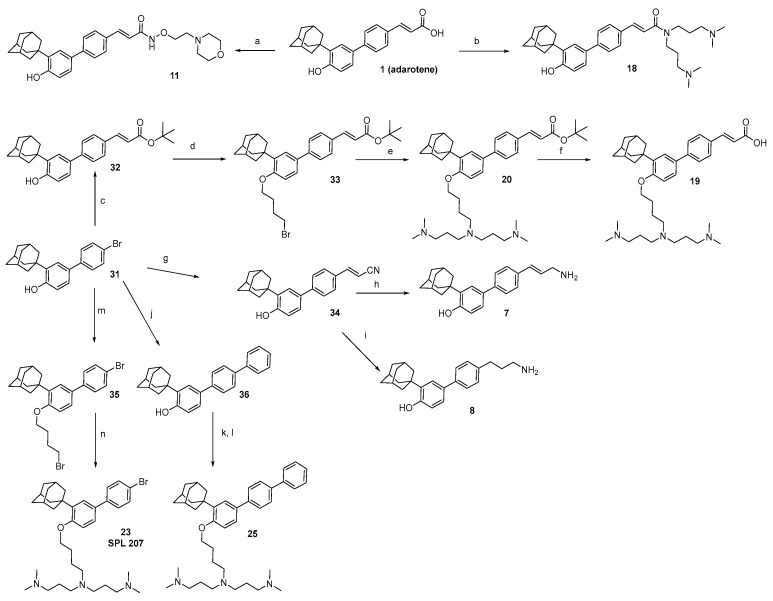
Reagents and conditions: (a) i. DIPEA, HBTU, DMF, 0 °C, 10 min.; ii. *O*-(2-morpholinoethyl)hydroxylamine hydrochloride, 48 h, rt, 24%; (b) *N*^1^-(3-(dimethylamino)propyl)-*N*^3^,*N*^3^-dimethylpropane-1,3-diamine, CH_2_Cl_2_, DIPEA, BOP, 48 h, rt, 54%; (c) *tert*-butyl acrylate, Pd(OAc)_2_, TEA, tri-*o*-tolylphosphine, 110 °C, 1 h, 90%; (d) K_2_CO_3_, 1,4-dibromobuthane, acetone, 6 h, reflux, 66%; (e) *N*^1^-(3-(dimethylamino)propyl)-*N*^3^,*N*^3^-dimethylpropane-1,3-diamine, DMF, 24 h, rt, 47%; (f) TFA, CH_2_Cl_2_, 2 h, 0 °C, quant.; (g) acrylonitrile, tri-*o*-tolylphosphine, Pd(OAc)_2_, TEA, 46%; (h) AlCl_3_, THF, 1 M LiAlH_4_ in THF, 1 h, 0 °C, 28%; (i) 1 M LiAlH_4_ in THF, 2 h, 0 °C, 15%; (j) phenylboronic acid, Pd(PPh_3_)_4_, K_2_CO_3_, H_2_O: THF (1: 2), 4 h, 80 °C, 46%; (k) 1,4-dibromobutane, K_2_CO_3_, acetone, 6 h, reflux, 40%; (l) *N*^1^-(3-(dimethylamino)propyl)-*N*^3^,*N*^3^-dimethylpropane-1,3-diamine, THF, 4 h, reflux, 20%; (m) 1,4-dibromobutane, K_2_CO_3_, acetone, 8 h, reflux, 65%; (n) *N*^1^-(3-(dimethylamino)propyl)-*N*^3^,*N*^3^-dimethylpropane-1,3-diamine, DMF, 16 h, rt, 42%.

Hydroxamate **11** and amide **18** were obtained from adarotene, by condensation with *O*-[2-(morpholin-4-yl)ethyl]hydroxylamine and *N*^1^-(3-(dimethylamino)propyl)-*N*^3^,*N*^3^-dimethylpropane-1,3-diamine, respectively. Common brominated intermediate **31** was employed to introduce a polyamine chain on different portions of the biphenyl adarotene-like skeleton (Scheme 2). Heck reaction with *t*-butyl acrylate promptly afforded *t*-butyl ester **32**, ready for further functionalization. Alkylation of the phenolic residue with 1,4-dibromobutane resulted in compound **33**, which was alkylated and final hydrolyzed to give acid **19**. Derivative **34** was obtained by Heck reaction with acrylonitrile, whose cyano group was reduced to allyl amine **7** and its saturated analog **8**. Suzuki coupling between bromine **31** and phenylboronic acid afforded compound **36**, which was *O*-alkylated with 1,4-dibromobutane and functionalized with *N*^1^-(3-(dimethylamino)propyl)-*N*^3^,*N*^3^-dimethylpropane-1,3-diamine to give derivative **25**. Application of the same synthetic route on **31** gave compound **23**.

**Scheme 3:**
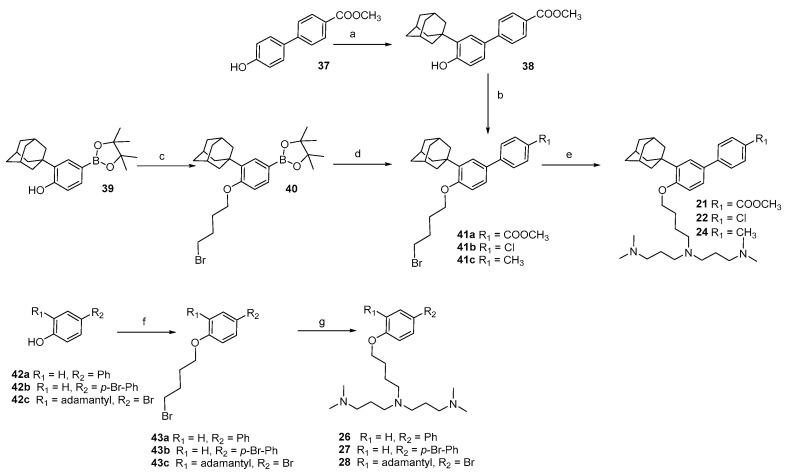
Reagents and conditions: (a) 1-adamantanol, H_2_SO_4_, 2 h, rt, 42%; (b) for **41a**: **38**, 1,4-dibromobuthane, K_2_CO_3_, acetone, 4 h, reflux, 53%; (c) 1,4-dibromobuthane, K_2_CO_3_, acetone, 4 h, reflux, 76%; (d) for **41b**: 1-bromo-4-chlorobenzene, THF: H_2_O (2: 1), K_2_CO_3_, Pd(PPh_3_)_4_, 4 h, 80 °C, 39%; for **41c**: 1-bromo-4-methylbenzene, THF: H_2_O (2: 1), K_2_CO_3_, Pd(PPh_3_)_4_, 4 h, 80 °C, 47%; (e) for **21**: **41a**, *N*^1^-(3-(dimethylamino)propyl)-*N*^3^,*N*^3^-dimethylpropane-1,3-diamine, THF, 6 h, reflux, 39%; for **22**: **41b**, *N*^1^-(3-(dimethylamino)propyl)-*N*^3^,*N*^3^-dimethylpropane-1,3-diamine, THF, 4 h, reflux, 24%; for **24**: **41c**, *N*^1^-(3-(dimethylamino)propyl)-*N*^3^,*N*^3^-dimethylpropane-1,3-diamine, THF, 4 h, reflux, 25%; (f) for **43a**: **42a**, 1,4-dibromobuthane, K_2_CO_3_, acetone, 3 h, reflux, 60%; for **43b**: **42b**, 1,4-dibromobuthane, K_2_CO_3_, acetone, 3 h, reflux, 65%; for **43c**: **42c**, 1,4-dibromobuthane, K_2_CO_3_, acetone, 5 h, reflux, 69%; (g) for **26**: **43a**, *N*^1^-(3-(dimethylamino)propyl)-*N*^3^,*N*^3^-dimethylpropane-1,3-diamine, DMF, overnight, rt, 24%; for **27**: **43b**, *N*^1^-(3-(dimethylamino)propyl)-*N*^3^,*N*^3^-dimethylpropane-1,3-diamine, THF, 3 h, reflux, 26%; for **28**: **43c**, *N*^1^-(3-(dimethylamino)propyl)-*N*^3^,*N*^3^-dimethylpropane-1,3-diamine, THF, 3 h, reflux, 24%.

A similar procedure was employed for the *O*-alkylation of ester **38**, obtained by Friedel-Crafts reaction of adamantanol on the biphenyl moiety (Scheme 3). In order to change the substituent at C(4′), treatment of boronate **39** [18] with dibromobutane (compound **40**) was followed by Suzuki-Myiaura coupling with chlorobenzene or toluene, to afford **41b** and **41c**, respectively. Substitution of the terminal bromide with the triamine resulted in the obtainment of compounds **21**, **22** and **24**. Installation of the basic side chain at the phenolic oxygen was carried out also on biphenyls **42a** and **42b**, as well as on adamantanyl bromophenol **42c**.

According to the eNTRy rules, incorporating protonatable nitrogen-containing groups into the retinoid biphenyl scaffold may facilitate the compound’s passage through the Gram-negative OM [6].

With this rationale, we initially evaluated a set of compounds (**3**–**6**) from our in-house collection, each bearing an amino group on the same carbon that carries the substituent in **AB473** (Figure 2, Table 1).

These compounds were tested against *S. aureus* (Gram-positive) and *E. coli* (Gram-negative) model strains by determining the MIC (Table 1). Although the compounds **4**, **5**, and **6** showed mild activity against the Gram-positive strain, they were inactive against the Gram-negative one. These results prompted us to explore the introduction of protonatable chains in other positions of the biphenyl scaffold.

We therefore evaluated adarotene analogs featuring chains that differ in length, polarity, and flexibility in place of the acrylic moiety, aiming to balance the lipophilic portions of the molecule with the protonatable groups (Figure 2).

Compound **7**, having a -CH_2_NH_2_ group in place of the carboxylic acid moiety of adarotene (Figure 2), retained a good activity against Gram-positive (MIC = 4 µM), not showing however any activity against Gram-negative. The increase in flexibility of the chain (compound **8**) did not give any improvement (MIC = 8 µM), whereas the introduction of a methylpyrazine group (compound **9**) completely abolished the activity. The derivative obtained by installing a morpholino moiety through an oxime spacer (compound **10**) was inactive against both *S. aureus* and *E. coli*, while a weak effect against *S. aureus* (MIC = 32 µM) was obtained using the hydroxamate of adarotene as a spacer for the morpholino group (compound **11**). Thus, the morpholino group was replaced with a primary amine (compound **12**); however, the compound remained inactive against *E. coli* while showing potent activity against *S. aureus* (MIC = 4 µM).

The removal of one of the aromatic rings (compounds **13** vs. **12**) produced a further decrease in activity against the Gram-positive strain. The introduction of other secondary and tertiary amino groups (compounds **14**, **15**, and **16**) confirmed a good activity against the Gram-positive bacterium (MIC = 8–64 µM) but still no effect against the Gram-negative one.

Interestingly, compound **17**, carrying a polyamine chain in place of the acrylic moiety, showed moderate activity against *E. coli*, with MIC = 32 µM, retaining also activity against *S. aureus* (MIC = 16 µM). Moreover, the insertion of a polyamine chain through an amide bond with the carboxylic group of adarotene gave compound **18** with good activity against both *E. coli* and *S. aureus* (MIC = 32 µM and 8 µM, respectively).

The antimicrobial activity of compounds **7**, **12**, and **17** was also tested against other representative Gram-negative pathogens (Table S1). Compounds **7** and **12** were not active (MIC values > 128 μM), while compound **17** showed mild activity against *A. baumannii*, *E. coli*, *K. pneumoniae* and *P. aeruginosa* strains, with MIC values ranging from 64 µM to 128 µM (Table S1).

Successively, we focused our attention on the position of the polyamine moieties. We thus installed a branched protonatable chain on the phenolic OH. The acid (compound **19**) showed a comparable MICs (MIC = 8 and 16 µM) against both *S. aureus* and *E. coli*, respectively, while the corresponding *t*-butyl ester (compound **20**) showed reduced activity against *E. coli* (MIC = 64 µM). Antibacterial activity was improved by directly linking an ester group to the aromatic system. Notably, compound **21** exhibited good activity against *S. aureus* and *E. coli*, with MIC values of 4 µM and 8 µM, respectively. Accordingly, we evaluated the effect of introducing various substituents on the aromatic ring while keeping the rest of the molecular structure unchanged (compounds **22**–**25**). The same range of activities of compound **21** was retained in compounds **22**, **23**, and **24**, having a chloro, bromo, and methyl group, respectively, in place of the ester moiety.

A similar activity was maintained after the removal of one of the phenyl groups (compound **28**), whereas the introduction of a third phenyl group (compound **25**) abolished the activity against the Gram-negative strain (MIC = 128 µM). A further simplification of the scaffold with the removal of the adamantyl group (compounds **26**, **27**) caused an overall reduction in the activity, even against the Gram-positive strain.

This initial screening allowed the identification of compounds capable of inhibiting the growth of a Gram-negative bacterium such as *E. coli*. For this reason, the active compounds were subsequently tested against another opportunistic Gram-negative pathogen, the more clinically challenging *P. aeruginosa*. As reported in Table 2, compounds **18**, **19**, **20**, and **22** were inactive (MIC ≥ 128 µM). Compounds **21** and **28** showed a mild activity with MIC = 64 µM, while the most active compound resulted to be compound **23** (MIC = 32 µM), which was also tested against other Gram-negatives (*A. baumannii*, *E. cloacae*, *E. coli* MG1655, and *K. pneumoniae*; Table 3). The results showed MIC values ranging from 8 µM to 64 µM, confirming compound **23** (hereafter referred to as **SPL207**) as the most promising candidate for further investigation. As a preliminary assessment of potential cytotoxicity, the effect of the compound **SPL207** on eukaryotic cells was tested against the human alveolar lung epithelial A549 cells and the human keratinocyte HaCaT cells (Figures S3 and S4). Compounds **7**, **12** and **18** were also included. As reported in Figure S3, for the A549 cell line, the percentage of cell viability remained above 80% upon treatment with all four tested compounds up to a concentration of 16 μM. In the case of the HaCaT cell line (Figure S4), viability remained at 100% up to 16 μM for all four compounds. Treatment with compound **7** did not affect cell viability even at concentrations up to 32 μM. In any case, the concentrations at which an effect on cell viability was observed are higher than the MIC values of the selected molecules against *S. aureus* and most of the tested Gram-negative strains, except for *P. aeruginosa*.

**Figure 3:**
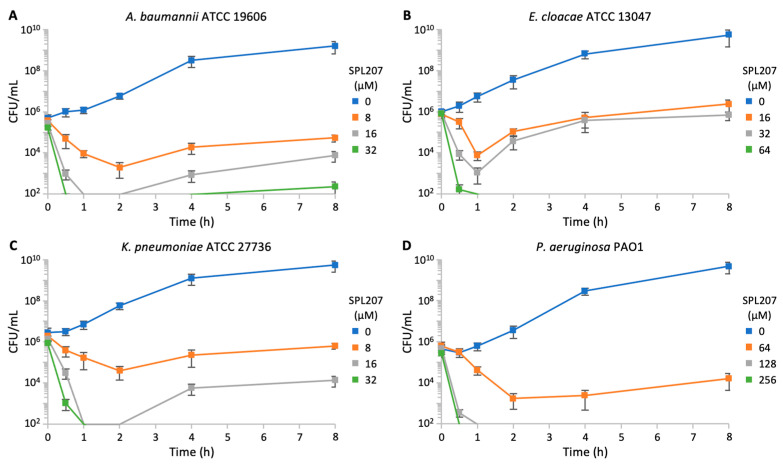
Time-kill curves of (**A**) *A. baumannii* ATCC 19606, (**B**) *E. cloacae* ATCC 13047, (**C**) *K. pneumoniae* ATCC 27736 and (**D**) *P. aeruginosa* PAO1 treated with **SPL207** at 1× MIC (orange lines), 2× MIC (gray lines) or 4× MIC (green lines). The untreated controls are shown with blue lines. Data are mean values from three independent experiments with standard deviations. The detection limit of the assay was 10^2^ CFU/mL.

Time-kill assays performed on *A. baumannii*, *E. cloacae*, *K. pneumoniae*, and *P. aeruginosa* cultures showed a drastic drop of CFU counts after treatment with **SPL207** at 2 × MIC and 4 × MIC (Figure 3). Hence, **SPL207** displays bactericidal activity.

### 2.2. Biology and Computational Analysis

#### 2.2.1. Investigation About *P. aeruginosa* Resistance to **SPL207**

We reasoned that the antimicrobial activity of **SPL207** against *P. aeruginosa* could be mainly weakened by efflux pumps capable of extruding this compound outside of the cell [19] and/or by low permeability of the OM [20,21]. To test these hypotheses, the activity of **SPL207** was assessed in *P. aeruginosa* mutant strains deleted in efflux pump genes or defective in OM biogenesis.

Since the efflux pumps MexAB-OprM, MexXY, MexCD-OprJ, and MexEF-OprN play a key role in *P. aeruginosa* resistance to many antimicrobial agents [19,22], MIC assays were performed on PAO1-KP ∆*efflux*, a *P. aeruginosa* PAO1 mutant with deletion of the *mexAB-oprM*, *mexXY*, *mexCD-oprJ*, and *mexEF-oprN* operons [23,24]. As reported in Table 4, **SPL207** showed the same MIC in PAO1-KP ∆*efflux* and its isogenic wild-type strain PAO1-KP, indicating that efflux pumps are not responsible for poor activity of **SPL207** in *P. aeruginosa*.

MIC assays were also performed in a *P. aeruginosa* PAO1 mutant strains with increased OM permeability. These strains are conditional mutants in which *lptE* or *lptH* expression can be induced by arabinose supplementation. LptE and LptH proteins are involved in the transport of lipopolysaccharides to the OM, hence low expression of *lptE* or *lptH* affects OM biogenesis and increases OM permeability [25,26].

Preliminary experiments were performed to identify the concentrations of arabinose that support growth of the *lptE* and *lptH* conditional mutants. In agreement with literature data [25,26], in the absence of arabinose *P. aeruginosa* growth was slightly reduced or abolished in the *lptE* and *lptH* conditional mutants, respectively, while arabinose supplementation did not affect PAO1 wild-type growth rate (Figure S1A,B). As shown in Table 4, the antimicrobial activity of **SPL207** increased when the *lptE* and *lptH* conditional mutants were grown in the presence of the lowest arabinose concentrations required to minimize their growth defect (i.e., 0.002% [*w*/*v*] and 0.125% [*w*/*v*] arabinose for the *lptE* and *lptH* conditional mutants, respectively). Conversely, MIC was restored to wild type levels when the conditional mutants were grown with high arabinose concentration (i.e., 0.5% [*w*/*v*] for both conditional mutants). Interestingly, increased activity of **SPL207** in *P. aeruginosa* was observed also in the presence of ethylenediaminetetraacetic acid (EDTA), a divalent cation chelating agent that increases OM permeability [27] (Table 4 and Figure S1C).

These results suggest that low OM permeability of *P. aeruginosa* could account for the limited activity of **SPL207** against this pathogen.

#### 2.2.2. Investigation of **SPL207** Mechanism of Action and Synergy with Colistin

**Figure 4:**
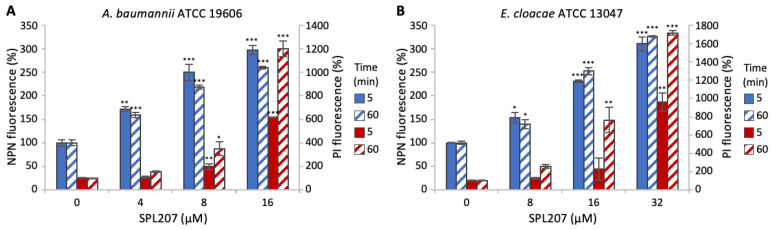

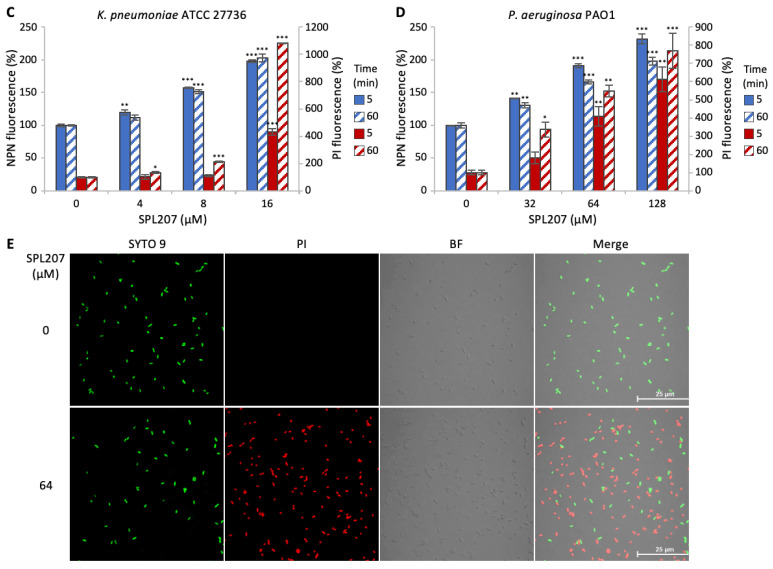
Effect of **SPL207** on bacterial membrane integrity. (**A**–**D**) Uptake of NPN (blue bars) or PI (red bars) by the (**A**) *A. baumannii* ATCC 19606, (**B**) *E. cloacae* ATCC 13047, (**C**) *K. pneumoniae* ATCC 27736, and (**D**) *P. aeruginosa* PAO1 strains after treatment for 5 min (full bars) or 60 min (striped bars) with increasing concentrations of **SPL207** (corresponding to 0.5×, 1×, and 2× MIC of **SPL207** for each strain), reported as fluorescence emission relative to untreated samples (control, considered as 100%). Data represent the mean of two independent experiments with standard deviations. Asterisks indicate a statistically significant difference with respect to the untreated samples (* *p* < 0.05, ** *p* < 0.01, *** *p* < 0.001; ANOVA). (**E**) Confocal microscopy images showing *P. aeruginosa* PAO1 cells incubated for 15 min in the absence or in the presence of 64 µM **SPL207**. From left to right: SYTO 9 fluorescence; PI fluorescence; BF, bright field; merge, overlap of bright field and fluorescence images.

Previous studies showed that adarotene derivatives can penetrate lipid bilayers and permeabilize the membranes of Gram-positives, ultimately causing cell lysis [12,14]. To investigate whether **SPL207** shares a similar mechanism of action in Gram-negative bacteria, its effect on membrane integrity was evaluated in *A. baumannii*, *E. cloacae*, *K. pneumoniae*, and *P. aeruginosa*. Briefly, a potential impact of **SPL207** on OM and inner membrane (IM) permeability was evaluated by fluorescent assays based on the probes *N*-phenyl-1-naphthylamine (NPN) and propidium iodide (PI) [28,29]. In all four strains tested, an increase in NPN and PI fluorescence was observed as the concentration of **SPL207** increased (Figure 4A–D). These results indicate that **SPL207** impairs the OM and IM integrity in all the Gram-negative bacteria tested.

Additionally, to evaluate a potential loss of *P. aeruginosa* envelope integrity in response to **SPL207**, a Live/Dead staining assay [30] was performed. As shown by confocal microscopy imaging, **SPL207** treatment strongly increased the fraction of red fluorescent PAO1 cells (Figure 4E), indicative of membrane damage and cell death (only these cells can uptake the red fluorescent dye PI). Impairment of membrane integrity in **SPL207** treated cells is in line with the fast-killing activity exerted by this compound (Figure 3D).

**Figure 5:**
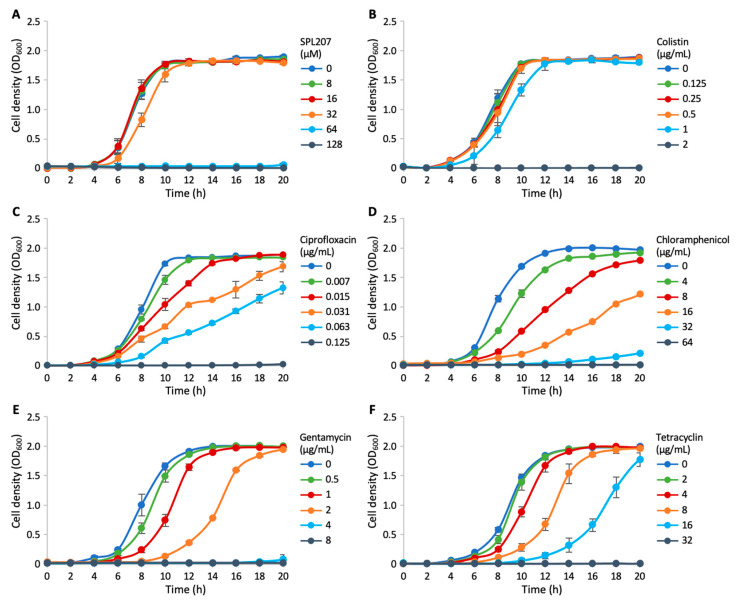
Growth curves of *P. aeruginosa* PAO1 strain grown in MHB-II supplemented with increasing concentrations of (**A**) **SPL207** (µM), (**B**) colistin (µg/mL), (**C**) ciprofloxacin (µg/mL), (**D**) chloramphenicol (µg/mL), (**E**) gentamycin (µg/mL), and (**F**) tetracycline (µg/mL). Data are mean values from three independent biological replicates with standard deviations.

These findings indicate that **SPL207** may exert its antibacterial effect via a mechanism akin to that of colistin, involving interaction with the OM and consequent membrane disruption [31]. Interestingly, similar to colistin and in contrast to antibiotics with different mechanisms of action (e.g., ciprofloxacin, chloramphenicol, gentamycin, and tetracycline), **SPL207** has no effect on the bacterial growth kinetics when at sub-MICs (Figure 5). The results of the permeability assays and the all-or-nothing effect shared by **SPL207** and colistin on *P. aeruginosa* growth support the mechanism of action of **SPL207** as a membrane perturbating agent.

Based on the similarities in the mechanisms of action of **SPL207** and colistin, the potential interaction between these two antimicrobials was evaluated. Checkerboard assays revealed that colistin and **SPL207** have a synergistic antimicrobial activity against *P. aeruginosa*, with a Fractional Inhibitory Concentration Index (FICI) = 0.5 (Table 5 and Figure S2; FICI ≤ 0.5 indicates synergy between antimicrobials) [32]. Conversely, checkerboard assays showed no interaction, either positive or negative, between **SPL207** and other two anti-*P. aeruginosa* antibiotics of clinical relevance such as ciprofloxacin or tobramycin. The effect of the colistin-**SPL207** combination was investigated also in *P. aeruginosa* colistin-resistant strains obtained by in vitro evolution experiments [33]. As reported in Table 5 and Figure S2, the MIC of colistin in these strains was higher than 4 μg/mL (corresponding to the colistin breakpoint in *P. aeruginosa* according to the EUCAST guidelines; i.e., colistin MIC ≤ 4 μg/mL, susceptible strain; colistin MIC > 4 μg/mL, resistant strain) [34]. The results demonstrated strong synergistic activity between colistin and **SPL207** against the three colistin-resistant strains tested (FICI ranging from 0.047 to 0.094; Table 5 and Figure S2). It is important to note that, in the presence of **SPL207** at concentrations of 8 μM (for PAO1 col^R^1), 2 μM (for PAO1 col^R^3), and 4 μM (for PAO1 col^R^5), the MIC of colistin against the tested strains decreased below the breakpoint [34].

Overall, **SPL207** possesses intrinsic antimicrobial activity and acts as a resistance breaker by resensitizing colistin-resistant *P. aeruginosa* strains to this last-resort antibiotic.

#### 2.2.3. Small Molecule/Membrane Interaction Analysis by MD Simulations

To provide atomistic details of the interaction between the most promising representative **SPL207** and the *P. aeruginosa* OM, extended molecular dynamics (MD) were carried out by adapting a computational protocol validated previously [35].

**Figure 6:**
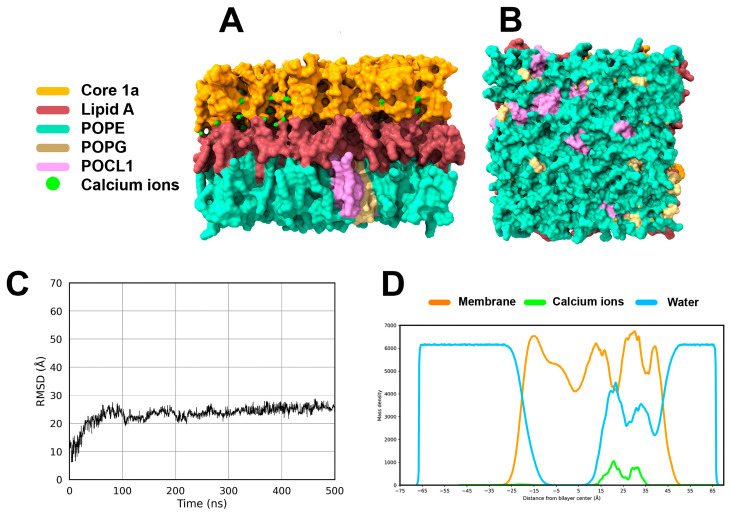
Representation of the *P. aeruginosa* OM model as obtained by 500 ns of MD simulation. (**A**,**B**) the representative frame extracted from the MD simulation is shown in a side and bottom view, respectively. Membrane components are shown as a colored surface: Core 1a (orange); Lipid A (brown); POPG (beige); POPE (light green); POCL1 (purple), while Ca^2+^ ions are represented as green spheres. (**C**). RMSD plot of the *P. aeruginosa* OM model along MD time. (**D**) mass density profile of water molecules (cyan), OM (orange), and Ca^2+^ ions (green).

The *P. aeruginosa* OM computational model was validated by 500 ns of all-atom MD simulations, analyzed through frames clustering, mass density distribution along the *z*-axis, and root mean square deviation (RMSD) (Figure 6). While the representative MD frame (i.e., the centroid frame of the most populated cluster) shows a homogeneous distribution of POPE, POPG, POCL1 phospholipids in the inner leaflet (Figure 6A,B), the RMSD plot indicates that the membrane model is conformationally stable in the MD simulation time (Figure 6C). Visual inspection of the mass density plot (Figure 6D) highlights the peculiar density of Ca^2+^ ions in the PA-LPS region, supporting their crucial role in stabilizing the OM of Gram-negative bacteria through electrostatic interactions with anionic components of the LPS [36,37,38]. Overall, this analysis suggests that the *P. aeruginosa* OM model is coherent with biological data, and it is suitable for ligand penetration studies in silico.

**Figure 7:**
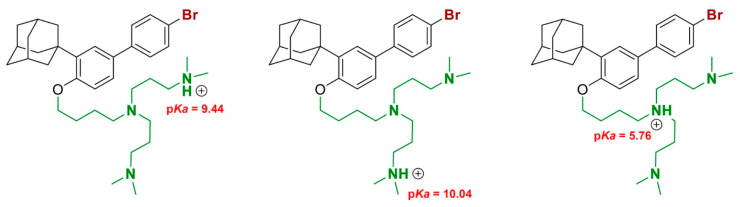
p*K*_a_ values of the amino groups in **SPL207** as predicted by the MoKa software [39].

To evaluate the interaction of **SPL207** and its parent compound adarotene (**1**) into the *P. aeruginosa* OM model, 500 ns of all-atom MD simulations were performed. The p*K*_a_ analysis of amino groups in **SPL207** carried out with the MoKa software version 4.0.12 [39] at pH = 7.4 (Figure 7) led to the selection of the protonated form of the molecule with total formal charge = +2 for this in silico study.

**Figure 8:**
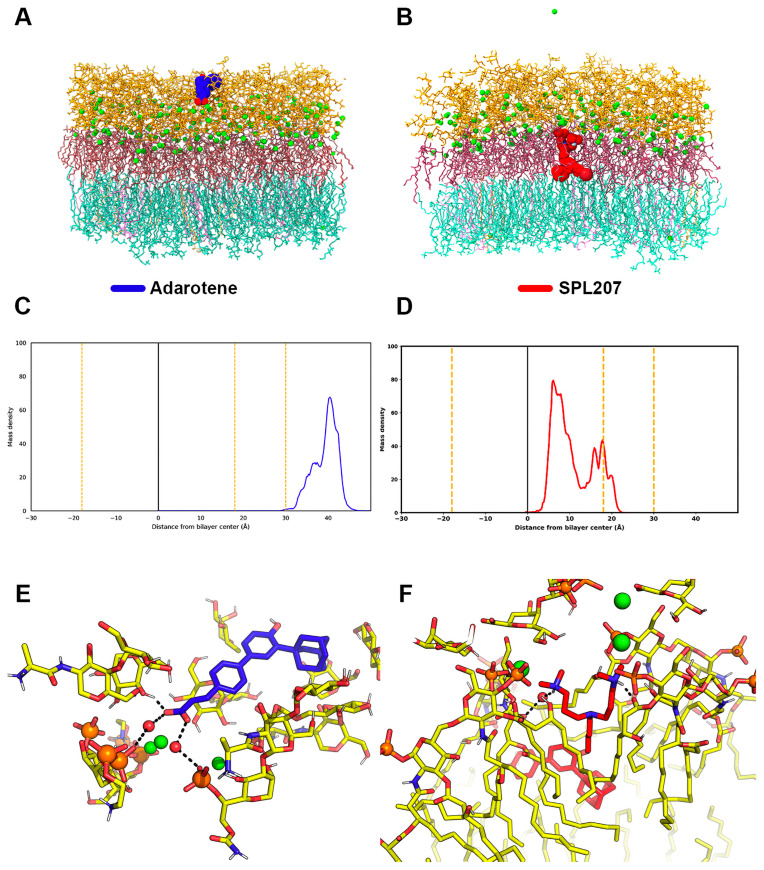
Analysis of MD trajectories with **1** and **SPL207**. (**A**,**B**) global view of the binding mode of **1** (**A**) and **SPL207** (**B**) to the *P. aeruginosa* OM model in the representative MD frames. The OM is represented as sticks, the Core 1a portion is colored orange, the Lipid A is colored brown, POPE is colored light green, POPG is colored beige, and POCL1 is colored purple; Ca^2+^ ions are represented as green spheres. **1** is shown as blue spheres, **SPL207** as red spheres. (**C**,**D**) mass density peak analysis for **1** ((**C**), blue line) and **SPL207** ((**D**), red line). The solid black line indicates the center of the membrane, while the density peaks of the phosphate barrier are represented by orange dashed lines. (**E**,**F**) Magnification of the binding mode of **1** ((**E**), blue sticks) and **SPL207** ((**F**), red sticks) within the *P. aeruginosa* OM as depicted by MD simulations. Water molecules involved in intermolecular interactions are shown as red spheres, Ca^2+^ ions as green spheres. Phosphate groups are shown as orange spheres. Polar contacts are highlighted by black dashed lines.

Cluster analysis of MD frames shows that, in the most populated cluster, **SPL207** penetrates deeply into the OM (Figure 8A) compared to **1**, this latter remaining locked within the PA-LPS region (Figure 8B). This behavior is also confirmed by the mass density plot, showing that **1** is unable to overcome the first phosphate layer of core 1a (orange dashed lines in Figure 8C), while **SPL207** successfully reaches the central part of the OM (Figure 8D). In evaluating atomistic details of the binding mode of **1** and **SPL207**, significant differences were observed. The carboxylic acid group of **1** (Figure 8E) establishes water-bridged H-bond interactions with the phosphate groups within the PA-LPS. The adamantyl moiety and the phenolic –OH group are projected towards the outer space and do not establish significant interactions with the *P. aeruginosa* OM model. In contrast, the positively charged amino groups of **SPL207** (Figure 8F) establish direct and water-bridged H-bonds interactions with negatively charged phosphate groups of the lipid A. This suggests that positively charged groups in **SPL207** may play a crucial role in OM embedding. Finally, the adamantyl moiety of **SPL207** is positioned towards the hydrophobic tails of POPE, POPG and POCL1 of the *P. aeruginosa* OM model.

#### 2.2.4. Displacement of Ca^2+^ Ions and Membrane Destabilization by **SPL207**

**Figure 9:**
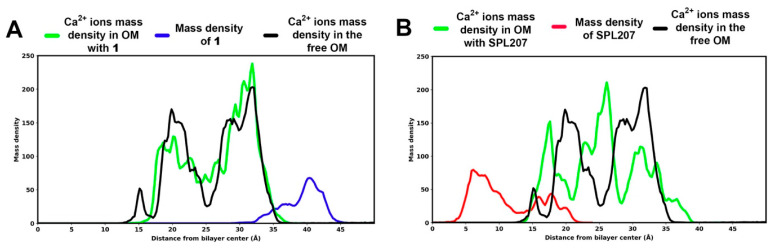
Mass density profile of Ca^2+^ ions within 20 Å of **1** ((**A**), blue line) and **SPL207** ((**B**), red line). In green line is represented the mass density of Ca^2+^ ions in the OM system with **1** and **SPL207**, respectively, in black line is represented the mass density of Ca^2+^ ions in the ligand-free OM system.

Based on the stabilizing role of Ca^2+^ ions in bacterial OMs, [34,35,36] it is expected that small molecules acting as membrane destabilizers can displace Ca^2+^ ions from the LPS. To assess this ability by **1** and **SPL207**, the mass density of Ca^2+^ ions within 20 Å of each molecule was monitored along MD trajectories (Figure 9). The mass density peak (MDP) of **1** fails to affect the mass density profile of Ca^2+^ ions, such as underlined by the comparison with the Ca^2+^ ions density in the free membrane (Figure 9A). In contrast, **SPL207** modifies the mass density profile of Ca^2+^ ions with respect to the free membrane (Figure 9B) suggesting that **SPL207** might displace Ca^2+^ ions more effectively than **1**. To further confirm these results, the mean squared displacement analysis of Ca^2+^ ions was carried out [40,41,42]. Results in Table 6 clearly show that **SPL207** imprints a significant Ca^2+^ ions displacement in the three axes compared to the baseline displacement in the free PA OM model, which is greater than that observed for **1**.

**Figure 10:**
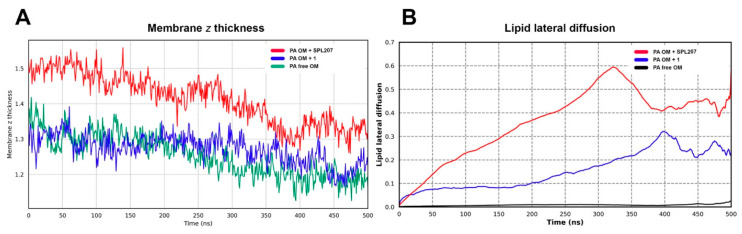
(**A**) Membrane *z* thickness analysis: green = ligand-free OM; blue = OM with **1**; red = OM with **SPL207**. (**B**) Lipid diffusion analysis: black = ligand-free OM; blue = OM with **1**; red = OM with **SPL207**.

Finally, the membrane *z* thickness and lipid diffusion analysis were calculated along MD trajectories as descriptors of the physical change that occur in the structure of the PA-OM. Membrane *z* thickness represents the distances in the *z* axis between the outer and the inner leaflets of the PA OM model, and its comparison in Figure 10A clearly shows that the free OM and the OM/**1** system are highly superimposable, whereas the presence of **SPL207** induces a greater movement of phospholipids along the *z* axis, further confirming **SPL207**’s ability to destabilize the PA OM in silico.

The analysis of lateral lipid diffusion further allows us to describe the movement of lipids along the *xy* axis over the MD trajectory. The results show that **SPL207** triggers a larger movement of lipids than **1**. Moreover, unlike compound **1**, **SPL207** induces a marked increase in lipid diffusion from the very beginning of the MD production trajectory.

Overall, MD results clearly point to the potential mechanism of action of **SPL207** as a membrane destabilizer through improved diffusion of Ca^2+^ ions and lipids destabilization.

## 3. Materials and Methods

### 3.1. Chemistry

All reagents and solvents were reagent grade or were purified by standard methods before use. ^1^H and ^13^C NMR spectra were recorded on Bruker AV600 (Billerica, MA, USA) spectrophotometer at 600 and 150 MHz, respectively, or Bruker Neo 400 spectrophotometer at 400 and 100 MHz, respectively. Chemical shifts are reported in ppm (δ) relative to TMS. The coupling constants, *J* are reported in Hertz (Hz). All compounds were routinely checked by thin layer chromatography (TLC) using precoated silica gel 60 F254, aluminum foil and the spots were detected under UV light at 254 nm and 365 nm or were revealed spraying with 10% phosphomolybdic acid (PMA) in ethanol.

Compounds **3**, **5**, **6**, **39** [18], **4** [13], **1**, **29a-b**, **34** [17], **30** [43], **31** [44], **32** [45], **42c** [46] were prepared according to reported procedures.

**3-((3r,5r,7r)-adamantan-1-yl)-4′-((*E*)-3-aminoprop-1-en-1-yl)-[1,1′-biphenyl]-4-ol (7)**. To a solution of AlCl_3_ (49 mg, 0.37 mmol) in dry THF (1.25 mL) 1 M LiAlH_4_ in THF (0.56 mL) was added at 0 °C and the solution was stirred 10 min, then compound **34** [17] (100 mg, 0.28 mmol) dissolved in THF (1.25 mL) was added at 0 °C. The mixture was stirred for 1 h at 0 °C, then quenched with water and 1M NaOH and extracted with AcOEt. The organic phase was dried with Na_2_SO_4_ and concentrated under reduced pressure. The crude was purified by preparative TLC (CH_2_Cl_2_: MeOH = 9:1) to afford product in 28% yield (28 mg) as a white solid. R*_f_* = 0.39 (CH_2_Cl_2_: MeOH = 9:1). ^1^H-NMR (400 MHz, DMSO-*d*_6_) δ: 7.56–7.49 (2H, m); 7.47–7.39 (2H, m); 7.37–7.27 (2H, m); 6.86 (1H, d, *J* = 8.3 Hz); 6.54 (1H, d, *J* = 16.0 Hz); 6.35 (1H, dt, *J* = 16.0 Hz, *J* = 5.7 Hz); 3.37 (2H, d, *J* = 5.7 Hz); 2.22–2.10 (6H, m); 2.09–2.01 (3H, m); 1.82–1.67 (6H, m). ^13^C-NMR (100 MHz, DMSO-*d*_6_) δ: 156.4; 140.1; 136.3; 135.4; 131.2; 130.7; 129.0; 127.0 (×2C); 126.5 (×2C); 125.0; 124.9; 117.3; 43.8; 40.3 (×3C); 37.1 (×3C); 36.7; 28.9 (×3C).

**3-((3r,5r,7r)-adamantan-1-yl)-4′-(3-aminopropyl)-[1,1′-biphenyl]-4-ol (8)**. To a solution of compound **34** (128 mg, 0.359 mmol) in dry THF (2.5 mL) 1 M LiAlH_4_ in THF (0.05 mL) was added at 0 °C. The mixture was stirred for 2 h at 0 °C, then quenched with water and 1 M NaOH and extracted with AcOEt. The organic phase was dried with Na_2_SO_4_ and concentrated under reduced pressure. The crude was purified by column chromatography (CH_2_Cl_2_: MeOH = 95:5 with 1% of conc. NH_3_ aqueous solution) to afford product **8** in 15% yield (20 mg) as a white solid. R*_f_* = 0.39 (CH_2_Cl_2_: MeOH = 9:1). ^1^H-NMR (400 MHz, CH_3_OH-*d*_4_) δ: 7.48–7.43 (2H, m); 7.36 (1H, d, *J* = 2.3 Hz); 7.27–7.21 (3H, m); 6.78 (1H, d, *J* = 8.2 Hz); 2.83–2.78 (2H, m); 2.72 (2H, t, *J* = 7.2 Hz); 2.27–2.20 (6H, m); 2.12–2.05 (3H, m); 1.95–1.79 (8H, m). ^13^C-NMR (100 MHz, CH_3_OH-*d*_4_) δ: 157.1; 140.5; 137.7; 132.2; 129.7 (×2C); 127.5 (×2C); 126.1; 125.8; 117.6; 41.6 (×3C); 41.4; 38.3 (×3C); 38.0; 33.5; 30.66 (×3C).

**3-((3r,5r,7r)-adamantan-1-yl)-4′-((4-methylpiperazin-1-yl)methyl)-[1,1′-biphenyl]-4-ol (9)**. To a suspension of aldehyde **29b** [17] (200 mg, 0.448 mmol) in CH_3_CN (1 mL) 1-methylpiperazine (49 mg, 0.49 mmol), formic acid (41 mg, 0.90 mmol) and BF_3_Et_2_O (0.002 mmol) were added and the mixture was heated at 85 °C for 6 h. The reaction was concentrated and treated with Et_2_O to obtain a precipitate. Compound **9** was obtained in 38% yield (70 mg) as a white solid upon chromatographic purification (CH_2_Cl_2_: MeOH = 20: 1 with 1% of conc. NH_3_). ^1^H-NMR (400 MHz, DMSO-*d*_6_) δ: 9.42 (1H, s); 7.52–7.45 (2H, m); 7.35–7.23 (4H, m); 6.84 (1H, d, *J* = 8.1 Hz); 3.46 (2H, s); 2.48–2.20 (8H, m); 2.18–2.10 (9H, m); 2.07–2.00 (3H, m); 1.82–1.70 (6H, m). ^13^C-NMR (100 MHz, DMSO-*d*_6_) δ: 155.8; 139.6; 136.2; 135.8; 130.6; 129.3 (×2C); 125.8 (×2C); 124.7 (×2C); 116.8; 61.8; 54.7 (×2C); 52.6 (×2C); 45.8; 39.9 (×3C); 36.6 (×3C); 36.3; 28.4 (×3C).

**(*E*)-3′-((3r,5r,7r)-adamantan-1-yl)-4′-hydroxy-[1,1′-biphenyl]-4-carbaldehyde *O*-(2-morpholinoethyl) oxime (10)**. To a solution of compound **29a** [17] (20 mg, 0.06 mmol) in EtOH (0.5 mL) *O*-(2-morpholinoethyl)hydroxylamine hydrochloride (22 mg, 0.12 mmol) and pyridine (95 mg, 1.2 mmol) were added and the resulting mixture was refluxed for 5 h. The reaction was concentrated and treated with H_2_O to obtain product **10** as a white precipitate in 67% yield (18 mg) without further purification. R*_f_* = 0.14 (TLC hexane: acetone = 7:3). ^1^H-NMR (400 MHz, DMSO-*d*_6_) δ: 9.54 (1H, s); 8.26 (1H, s); 7.68–7.57 (4H, m); 7.39–7.30 (2H, m); 6.87 (1H, d, *J* = 8.0 Hz); 4.24 (2H, t, *J* = 5.8 Hz); 3.63–3.51 (4H, m); 2.63 (2H, t, *J* = 5.8 Hz); 2.48–2.40 (4H, m); 2.20–2.10 (6H, m); 2.09–2.00 (3H, m); 1.83–1.67 (6H, m). ^13^C-NMR (100 MHz, DMSO-*d*_6_) δ: 156.3; 148.5; 142.3; 135.9;129.8; 129.8; 127.3 (×2C); 126.2 (×2C); 124.9; 124.7; 116.9; 71.3; 66.2 (×2C); 57.0; 53.6 (×2C); 39.8 (×3C); 36.6 (×3C); 36.3; 28.4 (×3C).

**(*E*)-3-(3′-((3r,5r,7r)-adamantan-1-yl)-4′-hydroxy-[1,1′-biphenyl]-4-yl)-*N*-(2-morpholinoethoxy)acrylamide (11)**. To a solution of compound **1** (adarotene) [17] (50 mg, 0.13 mmol) in DMF (2 mL) cooled to 0 °C DIPEA (52 mg, 0.40 mmol) and HBTU (51 mg, 0.13 mmol) were added and the resulting mixture was stirred for 10 min. After addition of *O*-(2-morpholinoethyl)hydroxylamine hydrochloride (24 mg, 0.13 mmol) the reaction was stirred for 48 h at room temperature and then concentrated and treated with H_2_O to obtain a precipitate. Product **11** was obtained in 24% yield (16 mg) as a sticky solid upon chromatographic purification (DCM: MeOH = 9:1). R*_f_* = 0.26 (DCM: MeOH = 9:1). ^1^H-NMR (400 MHz, CH_3_OH-*d*_4_) δ: 7.65 (1H, d, *J* = 15.8 Hz); 7.61–7.56 (4H, m); 7.42 (1H, d, *J* = 2.3 Hz); 7.31 (1H, dd, *J* = 2.3 Hz, 8.3 Hz); 6.82 (1H, d, *J* = 8.3 Hz); 6.46 (1H, d, *J* = 15.8 Hz); 4.13 (2H, t, *J* = 5.2 Hz); 3.78–3.71 (4H, m); 2.76 (2H, t, *J* = 5.2 Hz); 2.68–2.57 (4H, m); 2.27–2.18 (6H, m); 2.12–2.03 (3H, m); 1.92–1.80 (6H, m). ^13^C-NMR (100 MHz, CH_3_OH-*d*_4_) δ: 166.4; 157.8; 145.0; 142.5; 137.9; 133.7; 132.2; 129.4 (×2C); 127.8 (×2C); 126.2; 126.1; 117.8; 117.1; 74.4; 67.6 (×2C); 57.5; 54.8 (×2C); 41.5 (×3C); 38.3 (×3C); 38.0; 30.6 (×3C).

**(*E*)-3′-((3r,5r,7r)-adamantan-1-yl)-4′-hydroxy-[1,1′-biphenyl]-4-carbaldehyde *O*-(2-aminoethyl) oxime (12)**. To a solution of compound **29a** [17] (30 mg, 0.09 mmol) in EtOH, (0.5 mL) 2-(aminooxy)ethan-1-amine hydrochloride (27 mg, 0.18 mmol) and pyridine (142 mg, 1.80 mmol) were added and the resulting mixture was refluxed for 5 h. The reaction was concentrated under reduced pressure and the crude was finely shredded and washed with H_2_O to afford product **12** in 51% yield (18 mg) as a white solid without further purification. R*_f_* = 0.16 (reverse phase TLC MeOH: H_2_O = 9:1). ^1^H-NMR (600 MHz, CH_3_OH-*d*_4_) δ: 8.27 (1H, s); 7.22–7.66 (2H, m); 7.64–7.58 (2H, m); 7.43 (1H, d, *J* = 1.8 Hz); 7.32 (1H, dd, *J* = 1.8Hz, 8.4 Hz); 6.82 (1H, d, *J* = 8.4 Hz); 4.39 (2H, t, *J* = 4.9 Hz); signal overlapped with the solvent, 2.28–2.22 (6H, m); 2.12–2.07 (3H, m); 1.90–1.82 (6H, m). ^13^C-NMR (150 MHz, CH_3_OH-*d*_4_) δ: 157.9; 151.9; 145.3; 137.9; 132.2; 130.8; 128.7 (×2C); 127.6 (×2C); 126.2; 126.1; 117.8; 70.9; 41.5 (×3C); 40.3; 38.3 (×3C); 38.0; 30.6 (×3C).

**(*E*)-3-((3r,5r,7r)-adamantan-1-yl)-4-hydroxybenzaldehyde *O*-(2-aminoethyl) oxime (13)**. To a solution of compound **30** [43] (20 mg, 0.078 mmol) in EtOH, (0.5 mL) 2-(aminooxy)ethan-1-amine hydrochloride (23 mg, 0.156 mmol) and pyridine (123 mg, 1.56 mmol) were added and the resulting mixture was refluxed for 3 h. The reaction was concentrated, diluted with ethyl acetate, washed with H_2_O, dried with Na_2_SO_4_ and again concentrated under reduced pressure. Product **13** was obtained in 37% yield (9 mg) as a sticky solid without further purification. R*_f_* = 0.34 (reverse phase TLC MeOH: H_2_O = 9:1). ^1^H-NMR (400 MHz, CH_3_OH-*d*_4_) δ: 8.09 (1H, s); 7.43 (1H, d, *J* = 2.0 Hz); 7.25 (1H, dd, *J* = 2.0 Hz, 8.3 Hz); 6.74 (1H, d, *J* = 8.3 Hz); 4.19 (2H, t, *J* = 5.2 Hz); 3.03 (2H, t, *J* = 5.2 Hz); 2.20–2.13 (6H, m); 2.09–2.03 (3H, m); 1.86–1.79 (6H, m). ^13^C-NMR (100 MHz, CH_3_OH-*d*_4_) δ: 159.8; 151.5: 137.9; 127.1; 126.8; 124.3; 117.5; 74.5; 41.4; 41.4 (×3C); 38.2 (×3C); 37.9; 30.5 (×3C).

**3-((3r,5r,7r)-adamantan-1-yl)-4′-(((2-hydroxyethyl)amino)methyl)-[1,1′-biphenyl]-4-ol (14)**. Ethanolamine (5 mg, 0.09 mmol) was added dropwise to a solution of compound **29a** [17] (30 mg, 0.09 mmol) in MeOH (1.5 mL) at 0 °C and the resulting mixture was stirred for 18 h at room temperature. NaBH_4_ (7 mg, 0.18 mmol) was then gradually added at 0 °C and after stirring for 30 min the reaction was diluted with 1 M HCl. The aqueous phase was washed with CH_2_Cl_2_ in order to remove organic impurities, then added with 1 M NaOH and extracted with ethyl acetate. The organic phase was dried over anhydrous Na_2_SO_4_ and concentrated under reduced pressure to afford product **14** in 97% yield (32 mg) as a sticky yellow solid without further purification. R*_f_* = 0.32 (reverse phase TLC MeOH: H_2_O = 9:1). ^1^H-NMR (600 MHz, CH_3_OH-*d*_4_) δ: 7.54–7.49 (2H, m); 7.41–7.35 (3H, m); 7.26 (1H, dd, *J* = 2.1 Hz, 8.3 Hz); 6.80 (1H, d, *J* = 8.3 Hz); 3.83 (2H, s); 3.71 (2H, *J* = 5.5 Hz); 2.77 (2H, t, *J* = 8.3 Hz); 2.31–2.17 (6H, m); 2.13–2.05 (3H, m); 1.90–1.79 (6H, m). ^13^C-NMR (150 MHz, CH_3_OH-*d*_4_) δ: 157.3; 142.3; 138.1; 137.7; 133.0; 129.9 (×2C); 127.5 (×2C); 126.2; 125.9; 117.7; 61.4; 54.0; 51.6; 41.6 (×2C); 38.3 (×3C); 38.0; 30.6 (×3C).

**3-((3r,5r,7r)-adamantan-1-yl)-4′-(((2-(dimethylamino)ethyl)amino)methyl)-[1,1′-biphenyl]-4-ol (15)**. *N*^1^,*N*^1^-dimethylethane-1,2-diamine (11 mg, 0.12 mmol) was added dropwise to a solution of compound **29a** [17] (40 mg, 0.12 mmol) in MeOH (1.3 mL) at 0 °C and the resulting mixture was stirred for 18 h at room temperature. NaBH_3_CN (15 mg, 0.24 mmol) was then gradually added at 0 °C and after stirring for 40 min the reaction was concentrated under reduced pressure. The residue was treated with H_2_O and the resulting precipitate was filtered, washed with H_2_O and dried to afford product **15** in 84% yield (41 mg) as a white solid without further purification. R*_f_* = 0.14 (reverse phase TLC MeOH: H_2_O = 9:1). ^1^H-NMR (600 MHz, CH_3_OH-*d*_4_) *δ*: 8.42 (1H, s); 7.84–7.75 (2H, m); 7.69–7.61 (2H, m); 7.45 (1H, d, *J* = 2 Hz); 7.34 (1H, dd, *J* = 2 Hz, 8.2 Hz); 6.82 (1H, d, *J* = 8.2 Hz); 3.79 (2H, t, *J* = 6.7 Hz); 2.72 (2H, t, *J* = 6.7 Hz); 2.41–2.32 (3H, s); 2.31–2.20 (6H, m); 2.13–2.06 (3H, m); 1.92–1.80 (6H, m). ^13^C-NMR (150 MHz, CDCl_3_) *δ*: 157.9; 146.0; 137.8; 134.9; 132.2; 129.9 (×2C); 127.5 (×2C); 126.3; 126.2; 117.8; 60.7; 59.7; signal overlapped with the solvent; 45.8 (×2C); 41.5 (×3C); 38.3 (×3C); 38.0; 30.6 (×3C).

**3-((3r,5r,7r)-adamantan-1-yl)-4′-(((2-(2-hydroxyethoxy)ethyl)amino)methyl)-[1,1′-biphenyl]-4-ol (16)**. 2-(2-aminoethoxy)ethan-1-ol (9 mg, 0.09 mmol) was added dropwise to a solution of compound **29a** [17] (30 mg, 0.09 mmol) in MeOH (1.3 mL) at 0 °C and the resulting mixture was stirred for 18 h at room temperature. NaBH_3_CN (11 mg, 0.18 mmol) was then gradually added at 0 °C and after stirring for 30 min the reaction was concentrated under reduced pressure. The residue was treated with H_2_O and resulting precipitate was filtered, washed with H_2_O and dried to afford product **16** in 54% yield (24 mg) as a white solid without further purification. R*_f_* = 0.40 (reverse phase TLC MeOH: H_2_O = 9:1). ^1^H-NMR (400 MHz, CH_3_OH-*d*_4_) δ: 7.65–7.55 (2H, m); 7.51–7.43 (2H, m); 7.39 (1H, d, *J* = 2.1 Hz); 7.28 (1H, dd, *J* = 2.1 Hz, *J* = 8.2 Hz); 6.81 (1H, d, *J* = 8.2 Hz); 4.09 (2H, s); 3.77–3.67 (4H, m); 3.63–3.55 (2H, m); 3.15–3.05 (2H, m). ^13^C-NMR (100 MHz, CH_3_OH-*d*_4_) δ: 157.6; 143.7; 137.8; 133.0; 132.4; 130.9 (×2C); 127.9 (×2C); 126.2; 126.1; 117.7; 73.4; 68.1; 62.1; 52.7.0; 48.5; 41.5 (×3C); 38.3 (×3C); 38.0; 30.6 (×3C).

**3-((3r,5r,7r)-adamantan-1-yl)-4′-(((2-((2-aminoethyl)amino)ethyl)amino)methyl)-[1,1′-biphenyl]-4-ol (17)**. *N*^1^-(2-aminoethyl)ethane-1,2-diamine (24 mg, 0.23 mmol) was added dropwise to a solution of compound **29a** [17] (26 mg, 0.07 mmol) in MeOH (1.3 mL) at 0 °C and the resulting mixture was stirred for 18h at room temperature. NaBH_4_ (8 mg, 0.23 mmol) was then gradually added at 0 °C and after stirring for 30 min the reaction was diluted with 1 M HCl. The aqueous phase was washed with CH_2_Cl_2_ to remove organic impurities and added with 1 M NaOH. The resulting precipitate was filtered, washed with H_2_O and dried. Product **17** was obtained in 49% yield (14 mg) as a white solid upon crystallization from Et_2_O. R*_f_* = 0.08 (reverse phase TLC MeOH: H_2_O = 9:1). ^1^H-NMR (400 MHz, CH_3_OH-*d*_4_) *δ*: 7.54–7.46 (2H, m); 7.41–7.32 (3H, m); 7.25 (1H, dd, *J* = 2.2 Hz, 8.2 Hz); 6.79 (1H, d, *J* = 8.2 Hz); 3.79 (2H, s); 2.81–2.71 (6H, m); 2.71–2.63 (2H, m); 2.27–2.17 (6H, m); 2.11–2.03 (3H, m); 1.89–1.77 (6H, m). ^13^C-NMR (100 MHz, CH_3_OH-*d*_4_) *δ*: 157.3; 142.3; 138.4; 137.7; 133.0; 129.9 (× 2C); 127.5 (× 2C); 126.1; 125.9; 117.7; 54.2; 54.0; 52.3; 49.5; 41.7 (× 3C); 38.3 (× 3C); 38.0; 30.6 (× 3C).

**(*E*)-3-(3′-((3r,5r,7r)-adamantan-1-yl)-4′-hydroxy-[1,1′-biphenyl]-4-yl)-*N*,*N*-bis(3-(dimethylamino)propyl)acrylamide (18)**. To a solution of compound **1** (50 mg, 0.134 mmol) in dry CH_2_Cl_2_ (2 mL), DIPEA (163 mg, 1.3 mmol), *N*^1^-(3-(dimethylamino)propyl)-*N*^3^,*N*^3^-dimethylpropane-1,3-diamine (32 mg, 0.173 mmol) and BOP (77 mg, 0.173) were added and the resulting mixture was stirred for 48 h at room temperature. The reaction was diluted with CH_2_Cl_2_, washed with H_2_O, dried with Na_2_SO_4_ and concentrated under reduced pressure. The resulting precipitate was filtered, washed with H_2_O and dried. The crude was purified by column chromatography (CH_2_Cl_2_: MeOH = 93:7 with 1% of conc. NH_3_ aqueous solution) to afford product **18** in 54% yield (39 mg) as a sticky solid. R*_f_* = 0.20 (CH_2_Cl_2_: MeOH = 95:5 with 1% of conc. NH_3_ aqueous solution). ^1^H-NMR (600 MHz, CH_3_OH-*d*_4_) δ: 7.70–7.65 (2H, m); 7.63–7.58 (3H, m); 7.44 (1H, d, *J* = 2.0 Hz); 7.32 (1H, dd, *J* = 2.0 Hz, *J* = 8.1 Hz) 7.15 (1H, d, *J* = 15.2 Hz); 6.82 (1H, d, *J* = 8.1 hz); 3.62 (2H, t, *J* = 7.7 Hz); 3.52 (2H, t, *J* = 7.2 Hz); 2.54 (2H, m); 2.47–2.42 82H, m); 2.41 (6H, s); 2.34–2.28 (6H, s); 2.27–2.22 (6H, m); 2.12–2.05 83H, m); 1.93–1.87 (4H, m); 1.86–1.83 (6H, m). ^13^C-NMR (600 MHz, CH_3_OH-*d*_4_) δ: 169.1; 157.8; 144.8; 144.1; 137.9; 134.2; 132.2; 129.6 (×2C); 127.7 (×2C); 126.2; 126.0; 117.8; 117.6; 57.7; 57.2; 47.2; 45.8; 45.5 (×2C); 45.1 (×2C); 41.5 (×3C); 38.3 (×3C); 38.0; 30.6 (×3C); 28.2; 26.2.

***tert*-butyl (*E*)-3-(3′-((3r,5r,7r)-adamantan-1-yl)-4′-(4-(bis(3-(dimethylamino)propyl)amino)butoxy)-[1,1′-biphenyl]-4-yl)acrylate (20)**. To a solution of compound 1 (140 mg, 0.32 mmol) in acetone (5 mL), K_2_CO_3_ (225 mg, 1.62 mmol) and 1,4-dibromobuthane (526 mg, 2.43 mmol) were added and the resulting mixture was heated to reflux for 6 h. The reaction was concentrated, diluted with ethyl acetate, washed with H_2_O, dried over anhydrous Na_2_SO_4_ and concentrated again under reduced pressure. Product 33 was obtained in 66% yield (122 mg) as a white solid upon crystallization from hexane. R*_f_* = 0.75 (Hex: AcOEt = 8:2). ^1^H-NMR (600 MHz, CDCl_3_) δ: 7.64 (1H, d, *J* = 16.0 Hz); 7.62–7.55 (4H, m); 7.51 (1H, d, *J* = 2.1 Hz); 7.43 (1H, dd, *J* = 2.1 Hz, *J* = 8.6 Hz); 6.96 ( 1H, d, *J* = 8.6 Hz); 6.41 (1H, d, *J* = 16.2 Hz); 4.10 (2H, t, *J* = 6.2 Hz); 3.56 (2H, t, *J* = 6.6 Hz); 2.24–2.17 (8H, m); 2.16–2.07 (5H, m); 1.86–1.787 (6H, m); 1.58 (9H, s).

To a solution of the above intermediate **33** (50 mg, 0.09 mmol) in dry DMF (2.5 mL), *N*^1^-(3-(dimethylamino)propyl)-*N*^3^,*N*^3^-dimethylpropane-1,3-diamine (165 mg, 0.90 mmol) was added and the mixture was stirred for 24h. The reaction was diluted with ethyl acetate, washed with H_2_O, dried with Na_2_SO_4_ and concentrated under reduced pressure. Impurities were removed from the crude by dissolving them in Et_2_O to obtain product **20** in 47% yield (25 mg) as a sticky solid. R*_f_* = 0.32 (reverse phase TLC MeOH: H_2_O = 9:1). ^1^H-NMR (600 MHz, CDCl_3_) δ: 7.68–7.58 (5H, m); 7.50 (1H, d; *J* = 2.2 Hz); 7.47 (1H, dd, *J* = 2.2 Hz, *J* = 8.2 Hz); 7.06 (1H, d, *J* = 8.2 Hz); 6.45 (1H, d, *J* = 16.0 Hz); 4.16 (2H, t, *J* = 5.9 Hz); 3.52–3.43 (4H, m); 3.13 (6H, s); 2.72–2.60 (4H, m); 2.49–2.40 (2H, m); 2.30 (6H, m); 2.25–2.19 (6H, m); 2.16–2.05 (5H, m); 2.04–1.98 (4H, m); 1.90–1.79 (6H, m); 1.72–1.64 (2H, m); 1.54 (9H, s). ^13^C-NMR (150 MHz, CDCl_3_) δ: 168.2; 159.2; 144.8; 142.6; 139.6; 134.1; 133.7; 129.7 (×2C); 127.9 (×2C); 126.4; 126.3; 120.5; 113.9; 81.7; 68.1; 65.2; 63.7; 58.5; 51.3; 47.1; 45.3 (×4C); 42.0 (×3C); 38.3 (×4C); 30.6 (×3C); 28.5 (×3C); 27.9; 27.5; 23.7; 21.0.

(***E*)-3-(3′-((3r,5r,7r)-adamantan-1-yl)-4′-(4-(bis(3-(dimethylamino)propyl)amino)butoxy)-[1,1′-biphenyl]-4-yl)acrylic acid (19)**. To a solution of compound **20** (20 mg, 0.03 mmol) in dry CH_2_Cl_2_ (0.47 mL), trifluoroacetic acid (0.21 mL) was added at 0 °C and the resulting mixture was stirred at 0 °C for 2 h. The reaction was concentrated under reduced pressure. After the azeotropic removal of TFA with toluene, product **19** was obtained in quantitative yield (28 mg) as a sticky solid. R*_f_* = 0.57 (reverse phase TLC MeOH: H_2_O = 9:1). ^1^H-NMR (600 MHz, CH_3_OH-*d*_4_) δ: 7.73 (1H, d, *J* = 16.2 Hz); 7.70–7.61 (4H, m); 7.52 (1H, d, *J* = 2.1 Hz); 7.49 (1H, dd, *J* = 2.1 Hz, 8.6 Hz); 7.08 (1H, d, *J* = 8.6 Hz); 6.52 (1H, d, *J* = 16.2 Hz); 4.18 (2H, t, *J* = 6.1 Hz); 3.57–3.49 (4H, m); 3.31–3.26 (2H, m); 3.24–3.15 (10H, m); 2.99–2.92 (6H, m); 2.34–2.28 (2H, m); 2.27–2.18 (8H, m); 2.16–2.08 (5H, m); 2.07–1.99 (2H, m); 1.91–1.84 (6H, m). ^13^C-NMR (150 MHz, CH_3_OH-*d*_4_) δ: 170.6; 162.6; 146.1; 144.8; 139.8; 134.2; 133.8; 129.9 (×2C); 128.1 (×2C); 126.6; 126.4; 118.8; 114.0; 68.2; 65.8; 62.3; 55.7; 51.4; 49.5; 46.0; 45.8; 43.7 (×3C); 42.1 (×3C); 38.4 (×4C); 30.5 (×3C); 22.8; 21.1; 21.0.

**Methyl 3′-((3r,5r,7r)-adamantan-1-yl)-4′-(4-(bis(3-(dimethylamino)propyl)amino)butoxy)-[1,1′-biphenyl]-4-carboxylate (21)**. To a 0 °C cooled solution of methyl 4′-hydroxy(1,1′-biphenyl)-4-carboxylate **37** (100 mg, 0.438 mmol) and 1-adamantanol (73 mg, 0.48 mmol) in CH_2_Cl_2_ (3.5 mL), H_2_SO_4_ (0.05 mL) was added and the resulting mixture was stirred for 2 h. The reaction was diluted with cold H_2_O, extracted with CH_2_Cl_2_, dried over anhydrous Na_2_SO_4_ and concentrated under reduced pressure. The crude was purified by column chromatography (Hex: AcOEt = 8:2) to afford product **38** in 42% yield (67 mg). R*_f_* = 0.30 (Hex: AcOEt = 9:1). ^1^H-NMR (600 MHz, CDCl_3_) δ: 8.14–8.06 (2H, m); 7.68–7.61 (2H, m); 7.52 (1H, d, *J* = 2.2 Hz); 7.37 (1H, dd, *J* = 2.2 Hz, 8.2 Hz); 6.77 (1H, d, *J* = 8.2 Hz); 4.98 (1H, s); 3.97 (3H, s); 2.25–2.20 (6H, m); 2.18–2.11 (3H, m); 1.86–1.81 (6H, m).

To a solution of above intermediate **38** (33 mg, 0.09 mmol) in acetone (1.5 mL), K_2_CO_3_ (63 mg, 0.46 mmol) and 1,4-dibromobuthane (136 mg, 0.63 mmol) were added and the resulting mixture was refluxed for 4 h. The reaction was concentrated, diluted with ethyl acetate, washed with H_2_O, dried with Na_2_SO_4_ and concentrated again under reduced pressure. Product **41a** was obtained in 53% yield (23 mg) as a white solid upon crystallization from hexane. R*_f_* = 0.36 (Hex: AcOEt = 9:1). ^1^H-NMR (600 MHz, CDCl_3_) δ: 8.14–8.05 (2H, m); 7.70–7.60 (2H, m); 7.53 (1H, d, *J* = 2.2 Hz); 7.46 (1H, dd, *J* = 2.2 Hz, 8.4 Hz); 6.97 (1H, d, *J* = 8.4 Hz); 4.11 (2H, t, *J* = 6.0 Hz); 3.96 (3H, s); 3.56 (2H, t, *J* = 6.4 Hz); 2.26–2.17 (8H, m); 2.16–2.05 (5H, m); 1.87–1.78 (6H, m).

To a solution of compound **41a** (60 mg, 0.12 mmol) in THF (2 mL), *N*^1^-(3-(dimethylamino)propyl)-*N*^3^,*N*^3^-dimethylpropane-1,3-diamine (0.27 mL, 0.4 mmol) was added and the mixture was refluxed for 6 h. The reaction was diluted with ethyl acetate, washed with H_2_O, dried with Na_2_SO_4_ and concentrated under reduced pressure. Product **21** was obtained in 39% yield (4 mg) as a sticky solid upon chromatographic purification (CH_2_Cl_2_: MeOH: toluene = 4.5: 1.5: 4 with 1% of conc. NH_3_). R*_f_* = 0.23 (DCM: MeOH: toluene = 4.5: 1.5: 4 with 1% of conc. NH_3_). ^1^H-NMR (400 MHz, CDCl_3_) δ: 8.13–8.03 (2H, m); 7.68–7.62 (2H, m); 7.52 (1H, d, *J* = 2.0 Hz); 7.45 (1H, dd, *J* = 2.0 Hz, 8.3 Hz); 6.95 (1H, d, *J* = 8.3 Hz); 4.06 (2H, t, *J* = 6.1 Hz); 3.94 (3H, s); 2.60–2.46 (6H,.m); 2.41–2.33 (2H, m); 2.29 (12H, s); 2.23–2.19 (6H, m); 2.16–2.10 (3H, m); 1.97–1.85 (2H, m); 1.83–1.77 (6H, m); 1.76–1.62 (6H, m). ^13^C-NMR (100 MHz, CDCl_3_) δ: 167.3; 158.5; 146.1; 138.7; 131.8; 130.1 (×2C); 128.1; 126.6 (×2C); 125.9; 125.7; 112.4; 67.9; 57.9 (×2C); 53.9; 52.1; 52.0 (×2C); 45.4 (× 4C); 40.7 (×3C); 37.3 (×3C); 29.8; 29.2 (×3C); 27.6; 25.1 (×2C); 24.0.

***N*^1^-(4-((3-(adamantan-1-yl)-4′-chloro-[1,1′-biphenyl]-4-yl)oxy)butyl)-*N*^1^-(3-(dimethylamino)propyl)-*N*^3^,*N*^3^-dimethylpropane-1,3-diamine (22)**. To a solution of compound 39 [18] (463 mg, 1.30 mmol) in dry acetone (12 mL), K_2_CO_3_ (898 mg, 6.50 mmol) and 1,4-dibromobuthane (1.10 mL, 9.15 mmol) were added and the resulting mixture was refluxed for 4 h. The reaction was concentrated, diluted with ethyl acetate, washed with H_2_O, dried over anhydrous Na_2_SO_4_. In vacuo concentration followed by flash column chromatography in Hex: AcOEt 9: 1 furnished the intermediate 2-(3-((3r,5r,7r)-adamantan-1-yl)-4-(4-bromobutoxy)phenyl)-4,4,5,5-tetramethyl-1,3,2-dioxaborolane (40) in 76% yield (484 mg). ^1^H-NMR (600 MHz, CDCl_3_) δ: 7.32 (1H, d, *J* = 2.4 Hz); 7.27 (1H, dd, *J* = 2.4 Hz, 8.9 Hz); 6.74 (1H, d, *J* = 8.9 Hz); 4.01 (2H, t, *J* = 5.9 Hz); 3.54 (2H, t, *J* = 6.6 Hz); 2.23–2.15 (2H, m); 2.14–2.02 (11H, m); 1.85–1.72 (6H, m).

To a suspension of intermediate (**40**, 100 mg, 0.20 mmol) in THF: H_2_O 2: 1 (2.4 mL), 1-bromo-4-chlorobenzene (38 mg, 0.20 mmol), K_2_CO_3_ (69 mg, 0.50 mmol) and Pd(PPh_3_)_4_ (7 mg, 0.006 mmol) were sequentially added under N_2_ atmosphere, and the mixture was heated 4 h at 80 °C. The reaction was diluted with ethyl acetate, washed with H_2_O, dried over anhydrous Na_2_SO_4_ and concentrated under reduced pressure. The intermediate **41b** was obtained in 39% yield (37 mg) as a white solid upon crystallization from hexane. ^1^H-NMR (600 MHz, CDCl_3_) δ: 7.53–7.47 (2H, m); 7.44 (1H, d, *J* = 2.0 Hz); 7.41–7.34 (3H, m); 6.94 (1H, d, *J* = 8.3 Hz); 4.09 (2H, t*, J* = 5.9 Hz); 3.55 (2H, t, *J* = 6.5 Hz); 2.26–2.15 (8H; m); 2.15–2.08 (5H, m); 1.86–1.75 (6H, m).

To a solution of compound **41b** (37 mg, 0.09 mmol) in THF (0.8 mL), *N*^1^-(3-(dimethylamino)propyl)-*N*^3^,*N*^3^-dimethylpropane-1,3-diamine (174 µL, 0.78 mmol) was added and the mixture was refluxed for 4 h. The reaction was diluted with ethyl acetate, washed with H_2_O, dried with Na_2_SO_4_ and concentrated under reduced pressure. Product **22** was obtained in 24% yield (11 mg) as a sticky solid upon chromatographic purification (CH_2_Cl_2_: MeOH: toluene = 4.5: 1.5: 4 with 1% of conc. NH_3_). ^1^H-NMR (400 MHz, CDCl_3_) δ: 7.54–7.46 (2H, m); 7.43 (1H, d, *J* = 2.3 Hz); 7.40–7.33 (3H, m); 6.94 (1H, d, *J* = 8.7 Hz); 4.05 (2H, t, *J* = 5.9 Hz); 2.59–2.52 (2H, m); 2.52 −2.44 (4H, m); 2.25 (12H, s); 2.22–2.16 (6H, m); 2.15–2.07 (3H, m); 1.95–1.85 (2H, m); 1.84–1.78 (6H, m); 1.77–1.59 (6H, m). ^13^C-NMR (100 MHz, CDCl_3_) δ: 158.0; 140.2; 138.7; 132.5; 131.9; 128.8 (×2C); 128.1 (×2C); 125.6; 125.3; 112.3; 67.9; 58.1 (×2C); 54.0; 52.1 (×2C); 45.6 (×4C); 40.7 (×3C); 37.3 (×3C); 29.8; 29.2 (×3C); 27.7; 25.5 (×2C); 24.1.

***N*^1^-(4-((3-((3r,5r,7r)-adamantan-1-yl)-4′-methyl-[1,1′-biphenyl]-4-yl)oxy)butyl)-*N*^1^-(3-(dimethylamino)propyl)-*N*^3^,*N*^3^-dimethylpropane-1,3-diamine (24)**. To a suspension of intermediate (40) in THF: H_2_O 2: 1 (2.4 mL), 1-bromo-4-methylbenzene (34 mg, 0.20 mmol), K_2_CO_3_ (69 mg, 0.50 mmol) and Pd(PPh_3_)_4_ (7 mg, 0.006 mmol) were sequentially added under N_2_ atmosphere, and the mixture was heated 4 h at 80 °C. The reaction was diluted with ethyl acetate, washed with H_2_O, dried over anhydrous Na_2_SO_4_ and concentrated under reduced pressure. The intermediate (3r,5r,7r)-1-(4-(4-bromobutoxy)-4′-methyl-[1,1′-biphenyl]-3-yl)adamantane 41c was obtained in 47% yield (42 mg) as a white solid upon crystallization from hexane. ^1^H-NMR (400 MHz, CDCl_3_) δ: 7.51–7.44 (3H, m); 7.38 (1H, d, *J* = 2.3 Hz, 8.4 Hz); 7.27–7.21 (2H, m); 6.93 (1H, d, *J* = 8.4 Hz); 4.09 (2H, t, *J* = 6.0 Hz); 3.55 (2H, t, *J* = 6.5 Hz); 2.41 (3H, s); 2.23–2.16 (8H, m); 2.15–2.05 (5H, m); 1.88–1.77 (6H, m).

To a solution of compound **41c** (42 mg, 0.09 mmol) in THF (0.8 mL) *N*^1^-(3-(dimethylamino)propyl)-*N*^3^,*N*^3^-dimethylpropane-1,3-diamine (206 µL, 0.92 mmol) was added and the mixture was refluxed for 4 h. The reaction was diluted with ethyl acetate, washed with H_2_O, dried with Na_2_SO_4_ and concentrated under reduced pressure. Product **24** was obtained in 25% yield (13 mg) as a sticky solid upon chromatographic purification (CH_2_Cl_2_: MeOH: toluene = 4.5: 1.5: 4 with 1% of conc. NH_3_). ^1^H-NMR (600 MHz, CDCl_3_) δ: 7.48–7.41 (3H, m); 7.38–7.33 (1H, m); 7.24–7.18 (2H, m); 6.91 (1H, d, *J* = 8.5 Hz); 4.02 (2H, t, *J* = 6.2 Hz); 2.55–2.49 (2H, m); 2.49–2.42 (4H, m); 2.38 (3H, s); 2.31–2.25 (4H, m); 2.22 (12H, s); 2.20–2.14 (6H, m); 2.12–2.04 (3H, m); 1.90–1.83 (2H, m); 1.82–1.75 (6H, m); 1.74–1.56 (6H, m). ^13^C NMR (101 MHz, CDCl_3_) δ 157.51, 138.81, 138.32, 136.03, 133.09, 129.34 (×2C), 126.70 (×2C), 125.53, 125.05, 112.17, 67.78, 57.98 (×2C), 53.93, 52.06 (×2C), 45.51 (×4C), 40.66 (×3C), 37.24 (×3C), 37.16, 29.17 (×3C), 27.65, 25.44 (×2C), 24.06, 21.06.

***N*^1^-(4-((3-((3r,5r,7r)-adamantan-1-yl)-4′-bromo-[1,1′-biphenyl]-4-yl)oxy)butyl)-*N*^1^-(3-(dimethylamino)propyl)-*N*^3^,*N*^3^-dimethylpropane-1,3-diamine (23)**. To a solution of 3-((3r,5r,7r)-adamantan-1-yl)-4′-bromo-[1,1′-biphenyl]-4-ol 31 [17,44] (100 mg, 0.26 mmol) in dry acetone (3 mL), K_2_CO_3_ (180 mg, 1.3 mmol) and 1,4-dibromobuthane (392 mg, 1.82 mmol) were added and the resulting mixture was refluxed for 6 h. The reaction was concentrated, diluted with ethyl acetate, washed with H_2_O, dried with Na_2_SO_4_ and concentrated again under reduced pressure. Product 35 was obtained in 65% yield (87 mg) as a white solid upon crystallization from hexane. R*_f_* = 0.77 (Hex: AcOEt = 9:1). ^1^H-NMR (600 MHz, CDCl_3_) δ: 7.57–7.52 (2H, m); 7.48–7.42 (3H, m); 7.37 (1H, dd, *J* = 2.6 Hz, 8.7 Hz); 6.95 (1H, d, *J* = 8.7 Hz); 4.10 (2H, t, *J* = 6.2 Hz); 3.56 (2H, t, *J* = 6.5 Hz); 2.25–2.16 (8H, m); 2.15–2.04 (5H, m); 1.86–1.77 (6H, m).

To a solution of compound **35** (40 mg, 0.077 mmol) in dry DMF (1.5 mL), *N*^1^-(3-(dimethylamino)propyl)-*N*^3^,*N*^3^-dimethylpropane-1,3-diamine (144 mg, 0.77 mmol) was added and the mixture was stirred for 18 h. The reaction was diluted with ethyl acetate, washed with H_2_O, dried with Na_2_SO_4_ and concentrated under reduced pressure. Impurities were removed from the crude by dissolving them in Et_2_O: Hex= 1: 1 to obtain product **23** in 42% yield (20 mg) as a sticky solid. R*_f_* = 0.30 (CH_2_Cl_2_: MeOH = 8:2 with 1% of conc. NH_3_ aqueous solution). ^1^H-NMR (600 MHz, CH_3_OH-*d*_4_) δ: 7.59–7.53 (2H, m); 7.51–7.47 (2H, m); 7.43 (1H, d, *J* = 2.3 Hz); 7.41 (1H, dd, *J* = 2.3 Hz, 8.4 Hz); 7.03 (1H, d, *J* = 8.4 Hz); 4.10 (2H, t, *J* = 6.1 Hz); 2.64 (2H, t, *J* = 7.2 Hz); 2.60–2.54 (4H, m); 2.49–2.43 (4H, m); 2.33 (12H, s); 2.28–2.20 (6H, m); 2.15–2.08 (3H, m); 1.97–1.90 (2H, m); 1.90–1.85 (6H, m); 1.85–1.78 (2H, m); 1.77–1.69 (4H, m). ^13^C-NMR (150 MHz, CH_3_OH-*d*_4_) δ: 159.3; 142.0; 139.6; 133.1; 132.8 (×2C); 129.3 (×2C); 126.3; 126.1; 121.4; 113.6; 68.8; 58.8 (×2C); 54.7; 53.0 (×2C); 45.3 (×4C); 42.0 (×3C); 38.3 (×3C); 30.8; 30.6 (×3C); 28.7; 25.2 (×2C); 24.8.

***N*^1^-(4-((3-((3r,5r,7r)-adamantan-1-yl)-[1,1′:4′,1″-terphenyl]-4-yl)oxy)butyl)-*N*^1^-(3-(dimethylamino)propyl)-*N*^3^,*N*^3^-dimethylpropane-1,3-diamine (25)**. Compound 31 (100 mg, 0.26 mmol), phenylboronic acid (32 mg, 0.26 mmol), Pd(PPh_3_)_4_ (9 mg, 0.008 mmol) and K_2_CO_3_ (90 mg, 0.65 mmol) were placed under N_2_ atmosphere and then H_2_O (1 mL) and THF (2 mL) were added. The resulting mixture was heated at 80 °C for 4 h. The reaction was diluted with cold water, acidified with 1 M HCl and extracted with ethyl acetate. The organic phase was dried over anhydrous Na_2_SO_4_ and concentrated under reduced pressure. The residue was purified by flash column chromatography (petroleum ether: AcOEt = 95: 5) to obtain product 36 in 46% yield (46 mg) as a white solid. R*_f_* = 0.29 (Hex: AcOEt = 9:1). ^1^H-NMR (600 MHz, CDCl_3_) δ: 7.70–7.72 (6H, m); 7.55–7.52 (1H, m); 7.51–7.46 (2H, m); 7.40−7.35 (2H, m); 6.77 (1H, d, *J* = 8.0 Hz); 2.25–2.21 (6H, m); 2.18–2.11 (3H, m); 1.87–1.80 (6H, m).

To a solution of intermediate **36** (46 mg, 0.12 mmol) in acetone (1 mL), K_2_CO_3_ (83 mg, 0.6 mmol) and 1,4-dibromobuthane (181 mg, 0.84 mmol) were added and the resulting mixture was refluxed for 6 h. The reaction was concentrated, diluted with ethyl acetate, washed with H_2_O, dried over anhydrous Na_2_SO_4_ and concentrated again under reduced pressure. (3r,5r,7r)-1-(4-(4-bromobutoxy)-[1,1′:4′,1″-terphenyl]-3-yl)adamantane was obtained in 40% yield (25 mg) as a white solid upon crystallization from petroleum ether. R*_f_* = 0.53 (Hex: AcOEt = 9:1). ^1^H-NMR (400 MHz, CDCl_3_) δ: 7.72–7.61 (6H, m); 7.54 (1H, d, *J* = 2.5 Hz); 7.51–7.43 (3H, m); 7.41–7.34 (1H, m); 6.97 (1H, d, *J* = 8.4 Hz); 4.10 (2H, t, *J* = 6.0 Hz); 3.56 (2H, t, *J* = 6.0 Hz); 2.30–2.17 (8H, m); 2.16–2.03 (5H, m); 1.90–1.77 (6H, m).

To a solution of the above intermediate (17 mg, 0.033 mmol) in THF (1 mL), *N*^1^-(3-(dimethylamino)propyl)-*N*^3^,*N*^3^-dimethylpropane-1,3-diamine (62 mg, 0.33 mmol) was added and the mixture was refluxed for 4 h. The reaction was diluted with ethyl acetate, washed with H_2_O, dried over anhydrous Na_2_SO_4_ and concentrated under reduced pressure. Product **25** was obtained in 20% yield (4 mg) as a sticky solid upon chromatographic purification (CH_2_Cl_2_: MeOH: toluene = 4.5: 1.5: 4 with 1% of conc. NH_3_ aqueous solution). ^1^H-NMR (400 MHz, CDCl_3_) δ: 7.72–7.60 (6H, m); 7.53 (1H, d, *J* = 2.4 Hz); 7.51–7.42 (3H, m); 7.41–7.34 (1H, m); 6.97 (1H, d, *J* = 8.5 Hz); 4.07 (2H, t, *J* = 6.0 Hz); 2.60–2.53 (2H, m); 2.53–2.46 (4H, m); 2.37–2.30 (4H, m); 2.26 (12H, s); 2.24–2.20 (6H, m); 2.16–2.07 (3H, m); 1.96–1.86 (2H, m); 1.85–1.79 (6H, m); 1.77–1.61 (6H, m). ^13^C-NMR (600 MHz, CDCl_3_) δ: 157.8; 141.0; 140.6; 139.2; 132.6; 128.8 (×2C); 127.4 (×2C); 127.2 (×3C); 127.0 (×2C); 125.6; 125.2; 112.2; 67.8, 57.9 (×2C); 53.9; 52.0 (×2C); 45.5 (×4C); 40.6 (×3C); 37.2 (×3C); 29.7; 29.1 (×3C); 27.6; 25.4 (×2C); 24.0.

***N*^1^-(4-([1,1′-biphenyl]-4-yloxy)butyl)-*N*^1^-(3-(dimethylamino)propyl)-*N*^3^,*N*^3^-dimethylpropane-1,3-diamine (26)**. To a solution of 4-hydroxybiphenyl **42a** (200 mg, 0.18 mmol) in acetone (6 mL), K_2_CO_3_ (815 mg, 5.9 mmol) and 1,4-dibromobuthane (1902 mg, 8.8 mmol) were added and the resulting mixture was refluxed for 3 h. The reaction was concentrated, diluted with ethyl acetate, washed with H_2_O, dried over anhydrous Na_2_SO_4_ and concentrated again under reduced pressure. Product **43a** 4-(4-bromobutoxy)-1,1′-biphenyl was obtained in 60% yield (216 mg) as a white solid upon crystallization from hexane. R*_f_* = 0.71 (Hex: AcOEt = 9:1). ^1^H-NMR (600 MHz, CDCl_3_) δ: 7.60–7.53 (4H, m); 7.47–7.42 (2H, m); 7.35–7.31 (1H, m); 4.08 (1H, t, *J* = 6.3 Hz); 3.54 (1H, t, *J* = 6.6 Hz); 2.16–2.09 (2H, m); 2.04–1.97 (2H, m).

To a solution of intermediate **43a** (40 mg, 0.131 mmol) in dry DMF (2 mL), *N*^1^-(3-(dimethylamino)propyl)-*N*^3^,*N*^3^-dimethylpropane-1,3-diamine (245 mg, 1.31 mmol) was added and the mixture was stirred for 48 h. The reaction was diluted with ethyl acetate, washed with H_2_O, dried with Na_2_SO_4_ and concentrated under reduced pressure. Product **26** was obtained in 24% yield (13 mg) as a sticky solid without further purification. R*_f_* = 0.16 (CH_2_Cl_2_: MeOH = 9:1 with 1% of conc. NH_3_ aqueous solution).^1^H-NMR (600 MHz, CDCl_3_) δ: 7.60–7.58 (2H, m); 7.56–7.52 (2H, m); 7.46–7.41 (2H, m); 7.35–7.30 (1H, m); 7.01–6.97 (2H, m); 4.04 (2H, t, *J* = 6.2 Hz); 2.52 (2H, t, *J* = 7.4 Hz); 2.50–2.34 (4H, m); 2.32–2.28 (4H, m); 2.33 (12H, s); 1.88–1.77 (6H, m); 1.69–1.60 (2H, m). ^13^C-NMR (150 MHz, CDCl_3_) δ: 158.8; 141.0; 133.7; 128.8 (×2C); 128.2 (×2C); 126.8 (×2C); 126.7; 114.9 (×2C); 68.10, 58.2 (×2C); 52.3 (×2C); 45.7 (×4C); 27.5; 25.6 (×2C); 23.8.

***N*^1^-(4-((4′-bromo-[1,1′-biphenyl]-4-yl)oxy)butyl)-*N*^1^-(3-(dimethylamino)propyl)-*N*^3^,*N*^3^-dimethylpropane-1,3-diamine (27)**. To a solution of 4′-bromo-(1,1′-biphenyl)-4-ol **42b** (100 mg, 0.40 mmol) in acetone (3 mL), K_2_CO_3_ (276 mg, 2 mmol) and 1,4-dibromobuthane (605 mg, 2.8 mmol) were added and the resulting mixture was refluxed for 3 h. The reaction was concentrated, diluted with ethyl acetate, washed with H_2_O, dried with Na_2_SO_4_ and concentrated again under reduced pressure. Product **43b** was obtained in 65% yield (90 mg) as a white solid upon crystallization from petroleum ether. ^1^H-NMR (600 MHz, CDCl_3_) δ: 7.58–7.53 (2H, m); 7.52–7.47 82H, m); 7.46–7.40 (2H, m); 7.01–6.95 (2H, m); 4.06 (2H, t, *J* = 6.0 Hz); 3.53 (2H, t, *J* = 6.5 Hz); 2.16–2.07 (2H, m); 2.05–1.97 (2H, m).

To a solution of compound **43b** (56 mg, 0.16 mmol) in THF (3 mL), *N*^1^-(3-(dimethylamino)propyl)-*N*^3^,*N*^3^-dimethylpropane-1,3-diamine (300 mg, 1.6 mmol) was added and the mixture was refluxed for 3 h. The reaction was diluted with ethyl acetate, washed with H_2_O, dried over anhydrous Na_2_SO_4_ and concentrated under reduced pressure. Product **27** was obtained in 26% yield (20 mg) as a sticky solid upon chromatographic purification (CH_2_Cl_2_: MeOH: toluene = 4.5: 1.5: 4 with 1% of conc. NH_3_ aqueous solution). ^1^H-NMR (600 MHz, CDCl_3_) δ: 7.57–7.54 (2H, m); 7.53–7.48 (2H, m); 7.46–7.42 (2H, m); 7.01–6.95 (2H, m); 4.03 (2H, t, *J* = 6.5 Hz); 2.52 (2H, t, *J* = 7.4 Hz); 2.50–2.46 (4H, m); 2.33–2.27 (4H, m); 2.25 (12H, s); 1.87–1.80 (2H, m); 1.70–1.61 (6H, m). ^13^C-NMR (600 MHz, CDCl_3_) δ: 158.9; 139.8; 132.3; 131.8 (×2C); 128.3 (×2C); 127.9 (×2C); 120.7; 114.9 (×2C); 67.9; 57.9 (×2C); 53.8; 52.0 (×2C); 45.5 (×4C); 27.8; 25.3 (×2C); 23.6.

***N*^1^-(4-(2-((3r,5r,7r)-adamantan-1-yl)-4-bromophenoxy)butyl)-N1-(3-(dimethylamino)propyl)-*N*^3^,*N*^3^-dimethylpropane-1,3-diamine (28)**. To a solution of 2-((3r,5r,7r)-adamantan-1-yl)-4-bromophenol **42c** [46] (200 mg, 0.65 mmol) in acetone (5 mL), K_2_CO_3_ (450 mg, 3.25 mmol) and 1,4-dibromobuthane (983 mg, 4.5 mmol) were added and the resulting mixture was refluxed for 5h. The reaction was concentrated, diluted with ethyl acetate, washed with H_2_O, dried with Na_2_SO_4_ and concentrated again under reduced pressure. The intermediate (3r,5r,7r)-1-(5-bromo-2-(4-bromobutoxy)phenyl)adamantane **43c** was obtained in 69% yield (198 mg) as a white solid upon crystallization from hexane. Rf = 0.78 (hex: AcOEt = 9:1). ^1^H-NMR (600 MHz, CDCl_3_) δ: 7.32 (1H, d, *J* = 2.4 Hz); 7.28 (1H, dd, *J* = 2.4 Hz, *J* = 8.7 Hz); 6.74 (1H, d, *J* = 8.7 Hz); 4.01 (2H, t, *J* = 6.0 Hz); 3.54 (2H, t, *J* = 6.5 Hz); 2.21–2.14 (2H, m); 2.12–2.09 (9H, m); 1.84–1.74 (6H, m).

To a solution of above compound **43c** (126 mg, 0.285 mmol) in THF (5 mL), *N*^1^-(3-(dimethylamino)propyl)-*N*^3^,*N*^3^-dimethylpropane-1,3-diamine (534 mg, 0.4 mmol) was added and the mixture was refluxed for 3h. The reaction was diluted with ethyl acetate, washed with H_2_O, dried over anhydrous Na_2_SO_4_ and concentrated under reduced pressure. Product **28** was obtained in 24% yield (38 mg) as a sticky solid upon chromatographic purification (CH_2_Cl_2_: MeOH: toluene = 4.5: 1.5: 4 with 1% of conc. NH_3_ aqueous solution). ^1^H-NMR (600 MHz, CDCl_3_) δ: 7.321(1H, d, *J* = 2.5 Hz); 7.26 (1H, dd, *J* = 2.5 Hz, *J* = 8.6 Hz); 6.74 (1H, d, *J* = 8.6 Hz); 3.97 (2H, t, *J* = 6.2 Hz); 2.53 (2H, t, *J* = 7.2 Hz); 2.48 (4H, t, *J* = 7.0 Hz); 2.34–2.28 (4H, m); 2.25 (12 H, s); 2.14–2.06 (9H, m); 1.91–1.84 (2H, m); 1.83–1.75 (6H, m); 1.75–1.60 (6H, m). ^13^C-NMR (150 MHz, CDCl_3_) δ: 157.3; 140.6; 129.9; 129.4; 113.7; 68.1; 58.1 (×2C); 54.0; 52.2 (×2C); 45.6 (×4C); 40.5 (×3C); 37.4; 37.2 (×3C); 29.1 (×3C); 27.6; 25.6 (×2C); 24.1.

### 3.2. Bacterial Strains and Growth Conditions

The bacterial strains used in this study are listed in Table S2. All strains were routinely grown at 37 °C in Muller Hinton II broth cation-adjusted (MHB-II) in shaking conditions (200 rpm), or Lysogeny Broth medium (LB) supplemented with 1.5% (*w*/*v*) agar. When specified, growth media were supplemented with L-arabinose or EDTA at the indicated concentrations.

Stock solutions of the adarotene derivatives were prepared in dimethyl sulfoxide (DMSO) at concentrations of 12.8 mM; stock solutions of 25% (*w*/*v*) L-arabinose, 0.5 M EDTA (pH 8.0), 10 mg/mL colistin, 1 mg/mL tobramycin were prepared in water; the stock solution of 1 mg/mL ciprofloxacin was prepared in 0.1 M HCl.

Growth assays in liquid cultures were performed as follows. Bacterial strains were grown at 37 °C in MHB-II with shaking (for the PAO1 *lptE* and *lptH* strains, the medium was supplemented with 0.5% (*w*/*v*) L-arabinose). After 16 h, cultures were diluted in fresh medium to an optical density at 600 nm wavelength (OD_600_) of ≈0.001. 100 µL aliquots were dispensed into each well of 96-well microtiter plates, to which 100 µL of **SPL207**, colistin, ciprofloxacin, L-arabinose or EDTA at increasing concentrations were added. The OD_600_ of the cultures was recorded every 2 h using an automated luminometer-spectrophotometer plate reader Spark10M (Tecan). Results were obtained from at least three independent experiments.

### 3.3. Antimicrobial Assays

The Minimal Inhibitory Concentration (MIC) of the compounds tested in this study was determined with the standard microdilution method, according to Clinical and Laboratory Standards Institute guidelines [47]. Bacterial strains were grown in MHB-II at 37 °C in shaking conditions. After 8 h of growth, the cultures were diluted in fresh medium at an OD_600_ of ≈0.0005 (ca. 5 × 10^5^ CFU/mL) in 96-well microtiter plates in presence of increasing concentrations of each compound (or the solvent in which the compound was dissolved, as control). When required, L-arabinose or EDTA were added at the concentrations indicated in the text. The MIC values were visually evaluated after 24 h of incubation at 37 °C in static conditions. Results were obtained from at least three independent experiments.

Time–kill assays were performed as reported in [48]. Briefly, bacterial strains were inoculated in MHB-II and incubated at 37 °C with shaking. Overnight cultures were adjusted to an OD_600_ of ≈0.0005 (ca. 5 × 10^5^ CFU/mL) in fresh medium supplemented or not with increasing concentrations of SPL207. Bacterial cultures were incubated at 37 °C in shaking conditions and 100 µL aliquots were harvest at different time points. The number of CFU/mL was determined by serially diluting the cultures in MHB-II and plating them on MHB-II agar plates for CFU count.

### 3.4. Cell Viability Assay

The human type II alveolar epithelial cell line A549 and the human immortalized keratinocyte cell line HaCaT were obtained from the American Type Culture Collection (ATCC, Manassas, VA, USA) and AddexBio (San Diego, CA, USA), respectively. Both cell lines were maintained in Dulbecco’s modified Eagle’s medium (DMEM) supplemented with L-glutamine (2mM for A549 or 4mM for HaCaT cells), 10% fetal bovine serum (FBS) and 0.1 mg/mL penicillin-streptomycin.

The effect of compounds **7**, **12**, **SPL207** and **18** on the viability of mammalian cells was determined by the inhibition of 3(4,5-dimethylthiazol-2yl)2,5-diphenyltetrazolium bromide (MTT) reduction to insoluble formazan. Cells suspended in the corresponding culture medium supplemented with glutamine and 2% FBS without antibiotics were plated in triplicate wells of a microtiter plate (4 × 10^4^ cells/well). After overnight incubation at 37 °C in a 5% CO_2_ atmosphere, the medium was replaced with 100 μL fresh serum-free medium containing the compounds at different concentrations. The plate was incubated for 24 h at 37 °C in a 5% CO_2_ atmosphere and the culture medium was removed and replaced with Hank’s buffer (136 mM NaCl; 4.2 mM Na_2_HPO_4_; 4.4 mM KH_2_PO_4_; 5.4 mM KCl; 4.1 mM NaHCO_3_, pH 7.2, supplemented with 20 mM d-glucose) containing 0.5 mg/mL MTT. After 4 h incubation, the formazan crystals were dissolved by adding 100 μL of acidified isopropanol and viability was determined by absorbance measurements at 570 nm using a microplate reader (Infinite M200; Tecan, Salzburg, Austria). Cell viability was calculated with respect to untreated cells (in DMEM containing 1% DMSO).

### 3.5. Fluorescent Probe-Permeability Assays

Permeability assays were performed as previously described [49], with minor modifications. Briefly, overnight cultures were diluted in 5 mL of fresh MHB-II and grown for 6 h at 37 °C in shaking conditions. Then, bacteria were harvested by centrifugation, resuspended in 5 mM HEPES (pH 7.2) at an OD_600_ of ≈1.0 and dispensed in 96-well black microtiter plates in the presence of increasing concentration of **SPL207** and NPN or PI at final concentrations of 10 µM or 20 µg/mL, respectively. Fluorescence was measured in an automated luminometer-spectrophotometer plate reader Spark10M (Tecan) after 5 min or 60 min at room temperature (λ_ex_ 350 nm and λ_em_ 420 nm, for NPN; λ_ex_ 580 nm and λ_em_ 620 nm, for PI).

Membrane permeabilization was also visualized by confocal laser scanning microscopy, as previously described [30], with minor modifications. Overnight cultures were diluted in 5 mL of fresh MHB-II and grown for 6 h at 37 °C in shaking conditions. Then, bacteria were harvested by centrifugation, washed with Phosphate-Buffered Saline (PBS, 1X), resuspended in PBS at an OD_600_ of ≈1.0, and dispensed in 96-well black microtiter plates in the presence of SYTO 9 and PI at final concentrations of 6 µM or 30 µM, respectively, in the presence or in the absence of 64 µM **SPL207**. After 15 min of incubation at room temperature in the dark, bacteria were washed with PBS and 10 µL aliquots of each sample were spotted on a microscope glass slide covered with 0.5% (*w*/*v*) agarose. Imaging was performed with a laser scanning confocal microscope Nikon A1R+, using 40× oil immersion objective. Images were acquired both in bright field and fluorescence channels using the following parameters: λ_ex_ 488 nm and λ_em_ ranging from 495 nm to 560 nm for SYTO 9; λ_ex_ 561 nm and λ_em_ ranging from 580 nm to 720 nm for PI. The NIS-elements software 6.1 was used to acquire and pre-process the images.

### 3.6. Checkerboard Assays

Checkerboard assays were performed as reported in [48]. Briefly, the antibiotic (i.e., colistin, ciprofloxacin, or tobramycin) was 2-fold diluted in MHB-II along the abscissa (x-axis), while **SPL207** (or DMSO as control) was 2-fold diluted in MHB-II along the ordinate (y-axis), allowing the testing of all possible combinations of the two compounds. The strains were grown in MHB-II at 37 °C in shaking conditions. After 8 h of growth, 100 µL of cultures diluted in fresh medium at an OD_600_ of ≈0.001 (ca. 1 × 10^6^ CFU/mL) was added to each well, previously filled with 100 µL containing the two compounds alone or in combination. After 20 h of incubation in static conditions at 37 °C, the MIC was determined as the lowest concentration of the antibiotic-**SPL207** combination that showed no visible bacterial growth. Results were obtained from at least three independent experiments.

### 3.7. System Preparation for MD Simulations

The asymmetric OM model of *P. aeruginosa* was built with the CHARMM-GUI Membrane Builder Tool [50,51]. The outer leaflet of the OM consists of 60 molecules of *P. aeruginosa* type 1 lipid A, core 1a lipopolysaccharide (PA-LPS, 100%) from CHARMM-GUI, while the inner leaflet contains 164 molecules of 1-palmitoyl-2-oleoyl-phosphatidylethanolamine (POPE, 90%), 9 molecules of 1-palmitoyl-2-oleoyl-phosphatidylglycerol (POPG, 5%) and 9 molecules of cardiolipin (POCL1, 5%) for a total of 60 PA-LPS and 182 phospholipid molecules, in agreement with the literature [52,53,54,55,56,57,58,59,60,61,62,63]. The system has a box length of 106.70 Å in the *xy* dimension and it is surrounded by a water layer with a thickness of 50 Å. 300 Ca^2+^ ions were added to each system, while Na^+^ and Cl^-^ ions were further added at a concentration of 0.15 M by the CHARMM-GUI tool, up to charge neutrality. In total, there were approximately 180,349 atoms in each simulation system.

Adarotene **1** and **SPL207** were sketched in 2D with the Picto software (version 4.5.4.1, OpenEye Cadence Molecular Sciences, Santa Fe, NM, USA) [64] and converted into a 3D structure with OMEGA (version 4.2.0.1, OpenEye Cadence Molecular Sciences, Santa Fe, NM) [65,66]. The protonation state of the molecule was predicted at pH 7.4 using the p*K*_a_ prediction software MoKa (Molecular Discovery, version 4.0.12) [39,67,68]; ligand energy minimization was performed with SZYBKI (version 2.5.0.1, OpenEye Cadence Molecular Sciences, Santa Fe, NM, USA) [69]. The two molecules were parameterized by the ligand reader and modeler module of CHARMM-GUI using the standard CHARMM force field (FF) [70]. The Multicomponent Assembler tool was used to randomly distribute the small molecules under investigation within the solvent area [71,72]. Following system construction with CHARMM-GUI, the topology and coordinate files were generated for AMBER [73,74].

### 3.8. MD Simulations

All-atom MD simulations were run with AMBER22, using a 2 fs time-step and a 10 Å non-bonded cut-off [75,76]. Each system underwent energy minimization for 50.000 steps (1.500 steps using the steepest descent algorithm, followed by 48.500 steps with the conjugate gradient algorithm). Subsequently, heating from 0 to 300 K was achieved over 900 ps at constant volume using the Langevin thermostat with a collision frequency of 2 ps^−1^, further keeping the temperature at 300 K at constant volume for 100 ps. Box density was equilibrated at constant pressure and constant temperature (300 K) over 1 ns using the Berendsen barostat. Following density equilibration, a preliminary 50 ns MD simulation was conducted at constant pressure. Subsequently, MD trajectories were generated for 500 ns. Analysis of MD trajectories was performed using the CPPTRAJ [77] software (version 6.18.1) and python packages like MDanalysis and LiPyphilic [78,79,80]. Small molecules interactions with the membrane were visually inspected with PyMol [81].

### 3.9. Statistical Analysis

Statistical analysis was performed with the software GraphPad Prism 5, using one-way analysis of variance (ANOVA) followed by Tukey–Kramer multiple comparison tests. Differences having a *p* value < 0.05 were considered statistically significant.

## 4. Conclusions

The search for novel antimicrobials is critical to combating the escalating threat of antibiotic resistance. In this study, we conjugated a retinoid-like scaffold—distinct from any currently used antibiotic class and originally active only against Gram-positive bacteria—with protonatable residues to promote penetration of the Gram-negative OM. We investigated the influence of various functional groups on enhancing permeation through the Gram-negative OM and identified **SPL207** as the most promising compound in the series. **SPL207** was shown to compromise membrane integrity across all tested Gram-negative species. Notably, it exhibited strong synergistic activity in combination with colistin against colistin-resistant strains. MD simulations provided atomistic insights into **SPL207**–membrane interactions, supporting the proposed mechanism of OM destabilization. Further studies are warranted to elucidate the structural determinants required to optimize the activity of these adarotene analogs against Gram-negative pathogens.

## Figures and Tables

**Table 1:** In vitro antimicrobial activity of adarotene derivatives against representative Gram-positive (*S. aureus*) and Gram-negative (*E. coli*) bacteria.

Compound	MIC (µM) *	
	*S. aureus* ATCC 25923	*E. coli* ATCC 25922
**2**	4 **	>128 **
**3**	>128	>128
**4**	64	>128
**5**	32	>128
**6**	32	>128
**7**	4	>128
**8**	8	>128
**9**	>128	>128
**10**	>128	>128
**11**	32	>128
**12**	4	>128
**13**	64	>128
**14**	8	>128
**15**	64	>128
**16**	64	>128
**17**	16	32
**18**	8	32
**19**	8	16
**20**	8	64
**21**	4	8
**22**	4	4
**23**	4	4
**24**	4	8
**25**	4	128
**26**	32	128
**27**	64	64
**28**	8	8

* Values represent the mode of at least three independent experiments; ** values taken from [14].

**Table 2:** MICs of active adarotene derivatives against *P. aeruginosa* ATCC 27853.

MIC (µM) *	
Compound	*P. aeruginosa* ATCC 27853
**18**	>128
**19**	128
**20**	128
**21**	64
**22**	128
**23**	32
**24**	128
**25**	>128
**26**	>128
**27**	>128
**28**	64

* Values represent the mode of at least three independent experiments.

**Table 3:** In vitro antimicrobial activity of the adarotene-derivative compound **SPL207**.

Strain	MIC SPL207 (µM) *
*A. baumannii* ATCC 19606	8
*A. baumannii* ACICU	8
*E. cloacae* ATCC 13047	16
*E. coli* MG1655	8
*K. pneumoniae* ATCC 27736	8
*P. aeruginosa* PAO1	64

* Values represent the mode of at least three independent experiments.

**Table 4:** In vitro antimicrobial activity of **SPL207** against *P. aeruginosa* PAO1 strains grown in MHB-II without or with EDTA (mM) or arabinose (%).

Strain	Growth Medium Supplemented with	MIC SPL207 (µM) *
PAO1-KP	-	64
PAO1-KP ∆*efflux*	-	64
PAO1 *lptE*	0.002% arabinose	16
	0.5% arabinose	64
PAO1 *lptH*	0.125% arabinose	16
	0.5% arabinose	64
PAO1	-	64
	0.4 mM EDTA	32
	0.8 mM EDTA	16

* Values represent the mode of at least three independent experiments.

**Table 5:** Effect of the **SPL207**-colistin combination on the MIC of the indicated *P. aeruginosa* strains.

Strain	Colistin MIC (µg/mL) at SPL207 conc. (µM) of:							Maximum Fold Change ^a^	Col_SPL207_ ^b^ MIC	SPL207_Col_^c^ MIC	FICI ^d^
	0	2	4	8	16	32	64				
PAO1	1	1	0.5	0.5	0.25	0.25	0.25	4	0.25	16	0.5
PAO1 col^R^1	64	8	8	4	2	2	2	32	2	16	0.094
PAO1 col^R^3	64	4	2	2	2	2	2	32	2	4	0.047
PAO1 col^R^5	8	8	1	0.5	0.5	0.5	0.5	16	0.5	8	0.094

^a^ Ratio between the MIC of colistin and the MIC of Col_SPL207_. ^b^ The MIC value of colistin in combination with **SPL207** used to calculate the FICI (µg/mL). ^c^ The MIC value of **SPL207** in combination with colistin used to calculate the FICI (µM). ^d^ Fractional inhibitory concentration index. For the PAO1 strain, the MIC of **SPL207** was 64 µM; for PAO1 col^R^1, PAO1 col^R^3 and PAO1 col^R^5, the MIC of **SPL207** was >128 µM, hence it was considered 256 µM for calculation of the FICI [FICI = (MIC Col_SPL207_/MIC Col) + (MIC **SPL207_Col_**/MIC **SPL207**)] [32].

**Table 6:** Diffusion coefficient for Ca^2+^ ions in PA OM model in the absence and in the presence of **SPL207** and **1**.

	Diffusion Coefficient [10^−7^ cm^2^/s]		
*System*	xy Plane	z Axis	Total
PA free OM	1.36 ± 0.032	0.38 ± 0.025	1.03 ± 0.051
PA–OM + **1**	2.90 ± 0.005	0.65 ± 0.049	2.15 ± 0.091
PA-OM + **SPL207**	4.74 ± 1.132	0.84 ± 0.057	4.73 ± 0.474

## Data Availability

Data are contained within the article and supplementary materials.

## Supplementary Materials

The following supporting information can be downloaded at: https://www.mdpi.com/article/10.3390/antibiotics14090956/s1, Table S1: In vitro antimicrobial activity of derivatives **7**, **12**, **17** against Gram-negative bacteria; Table S2: Bacterial strains used in this study; Figure S1: Growth curves of *P*. *aeruginosa* strains with arabinose or EDTA; Figure S2: Checkerboard assays with SPL207 and colistin; Figure S3: Cytotoxicity assays on A549 cells; Figure S4: Cytotoxicity assays on HaCaT cells; Figure S5: ^1^H-NMR and ^13^C-NMR of tested compounds.

## Abbreviations

BOP	Benzotriazole-1-yl-oxy-tris-(dimethylamino)-phosphoniumhexafluorophosphate
CFU	colony forming units
DIPEA	diisopropylethylamine
DMF	*N*,*N*-dimethylformamide
EDTA	ethylenediaminetetraacetic acid
EtOH	ethanol
FICI	fractional inhibitory concentration index
GPO	gram-positive only
HBTU	*N,N,N′,N′*-Tetramethyl-*O-*(1*H*-benzotriazol-1-yl)uronium hexafluorophosphate
IM	inner membrane
LPS	lipopolysaccharide
MeOH	methanol
MD	molecular dynamics
MDR	multidrug-resistant
MIC	minimum inhibitory concentration
MHB-II	Muller-Hinton II (broth)
NPN	*N*-phenyl-1-naphthylamine
OD	optical density
OM	outer membrane
PAO1	*Pseudomonas aeruginosa* (strain)
PI	propidium iodide
POPE	3-palmitoyl-2-oleoyl-D-glycero-1-Phosphatidylethanolamine
POPG	3-palmitoyl-2-oleoyl-D-glycero-1-Phosphatidylglycerol
POCL1	POPG + POPG cardiolipin with head group charge = −1
RMSD	root mean square deviation
rt	room temperature
SAR	structure-activity relationship
SPU	self-promoted uptake
TEA	triethylamine
TFA	trifluoroacetic acid
THF	tetrahydrofuran
