# Supplementary material for: Retargeting Gram-Positive-Only Adarotene-Derived Antibacterials to Broad-Spectrum Antibiotics
Source: Antibiotics (Basel). 2025 Sep 21;14(9):956. doi: 10.3390/antibiotics14090956 (PMC12466880; doi:10.3390/antibiotics14090956)

Retargeting Gram-positive-only adarotene-derived antibacterials to broad-spectrum antibiotics

Salvatore Princiotto [1,†], Luigi Cutarella[2,†], Alessandra Fortuna[3,†], Marta Mellini[3], Bruno Casciaro[4], Maria Rosa Loffredo[4], Alvaro G. Temprano[2,5], Floriana Cappiello[4], Livia Leoni[3], Maria Luisa Mangoni[4], Mattia Mori[2], Loana Musso [1], Francesca Sacchi [1], Cecilia Pinna[1], Giordano Rampioni[3,6,*], Sabrina Dallavalle[1,*] and Claudio Pisano[7]

[1] Department of Food, Environmental and Nutritional Sciences (DeFENS), University of Milan, via Celoria 2, 20133, Milan, Italy; salvatore.princiotto@unimi.it; loana.musso@unimi.it; francesca.sacchi@unimi.it; cecilia.pinna@unimi.it
[2] Department of Biotechnology, Chemistry and Pharmacy, University of Siena, via Aldo Moro 2, 53100 Siena, Italy. l.cutarella@student.unisi.it; mattia.mori@unisi.it
[3] Department of Science, University Roma Tre, Viale G. Marconi 446, 00146, Rome, Italy; alessandra.fortuna@uniroma3.it; marta.mellini@uniroma3.it; livia.leoni@uniroma3.it
[4] Pasteur Italia-Fondazione Cenci Bolognetti, Department of Biochemical Sciences, Sapienza University of Rome, 00185 Rome, Italy; bruno.casciaro@uniroma1.it; mariarosa.loffredo@gmail.com; floriana.cappiello@uniroma1.it; marialuisa.mangoni@uniroma1.it
[5] Experimental Hepatology and Drug Targeting (HEVEPHARM), University of Salamanca, IBSAL, Salamanca, Spain; Center for the Study of Liver and Gastrointestinal Diseases (CIBEREHD), Carlos III National Institute of Health, Madrid, Spain. alvarogacho@usal.es
[6] IRCCS Fondazione Santa Lucia, Via Ardeatina 306/354, 00179, Rome, Italy
[7] Special Products Line, 03012 Anagni, FR, Italy; c.pisano@specialspa.it

† These authors contributed equally to this work.
* Correspondence: sabrina.dallavalle@unimi.it; giordano.rampioni@uniroma3.it

**Table S1**. *In vitro* antimicrobial activity of derivatives **7**, **12**, **17** against Gram-negative bacteria	Pag. S1
**Table S2.** Bacterial strains used in this study	Pag. S1-S2
**Figure S1**. Growth curves of *P. aeruginosa* strains with arabinose or EDTA	Pag. S3
**Figure S2**. Checkerboard assays with **SPL207** and colistin	Pag. S4
**Figure S3.** Cytotoxicity assays on A549 cells	Pag. S5
**Figure S4.** Cytotoxicity assays on HaCaT cells	Pag. S6
**Figure S5.** ^{1}H-NMR and ^{13}C-NMR of tested compounds	Pag. S7-S28

Table S1. *In vitro* antimicrobial activity of derivatives 7, 12, 17 against Gram-negative bacteria

Strain	MIC (μM)		
	**7**	**12**	**17**
A. baumannii ATCC 19606	>128	>128	128
E. coli MG1655	>128	>128	64
K. pneumoniae ATCC 27736	>128	>128	64
P. aeruginosa PAO1	>128	>128	128

Table S2. Bacterial strains used in this study

Strains	Relevant characteristics	Reference/source
A. baumannii		
ATCC 19606^T	Type strain.	Janssen *et al.*, 1997
ACICU	MDR clinical isolate.	Iacono *et al.*, 2008
E. cloacae		
ATCC 13047	Type strain.	Hormaeche and Edwards, 1960
E. coli		
ATCC 25922	Reference strain.	ATCC
MG1655	K-12 ATCC 47076, type strain.	Bachman, 1996
K. pneumoniae		
ATCC 27736	Type strain.	ATCC
P. aeruginosa		
ATCC 27853	Reference strain.	ATCC
PAO1	ATCC 15692 type strain.	ATCC
PAO1 col^{R}1	*In vitro* evolved colistin-resistant PAO1 derivative.	Lo Sciuto and Imperi, 2018
PAO1 col^{R}3	*In vitro* evolved colistin-resistant PAO1 derivative.	Lo Sciuto and Imperi, 2018
PAO1 col^{R}5	*In vitro* evolved colistin-resistant PAO1 derivative.	Lo Sciuto and Imperi, 2018
PAO1 *lptE*	*lptE* conditional mutant of PAO1, carrying a deletion of the *lptE* gene and an arabinose-inducible copy of *lptE* inserted in the *attB* site of the chromosome.	Lo Sciuto *et al.*, 2018

PAO1 *lptH*	*lptH* conditional mutant of PAO1, carrying a deletion of the *lptH* gene and an arabinose-inducible copy of *lptH* inserted in the *attB* site of the chromosome.	Fernández-Piñar *et al.*, 2015
PAO1-KP	PAO1 wild type strain gently provided by Prof. K. Poole (Queen's University, Kingston, Canada).	Morita *et al.*, 2006
PAO1-KP Δ*efflux*	PAO1-KP mutant strain carrying mutations in the efflux pumps MexAB-OprM, MexCD-OprJ, MexEF-OprN and MexXY. Gently provided by Prof. K. Poole (Queen's University, Kingston, Canada).	Morita *et al.*, 2006
S. aureus		
ATCC 25923	Reference clinical strain.	ATCC

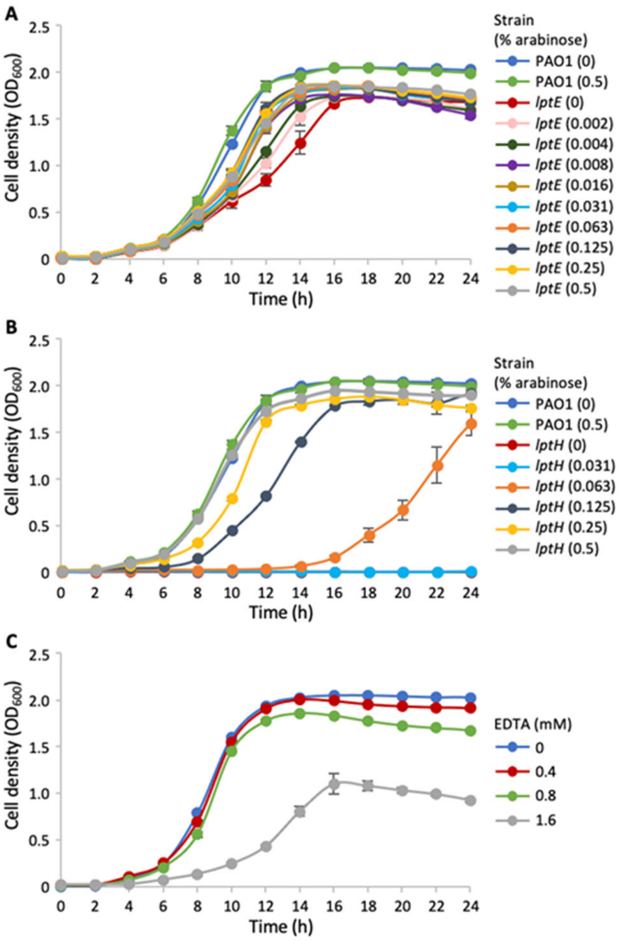

Figure S1. Growth curves of *P. aeruginosa* strains with arabinose or EDTA. **(A-B**) Growth of PAO1 and (**A**) the PAO1 *lptE* conditional mutant or (**B**) the PAO1 *lptH* conditional mutant in MHB-II supplemented with increasing concentrations of arabinose (0-0.5%). (**C**) Growth of PAO1 in MHB-II supplemented with increasing concentrations of EDTA (0-1.6 mM). Data are mean values from three independent biological replicates with standard deviations.

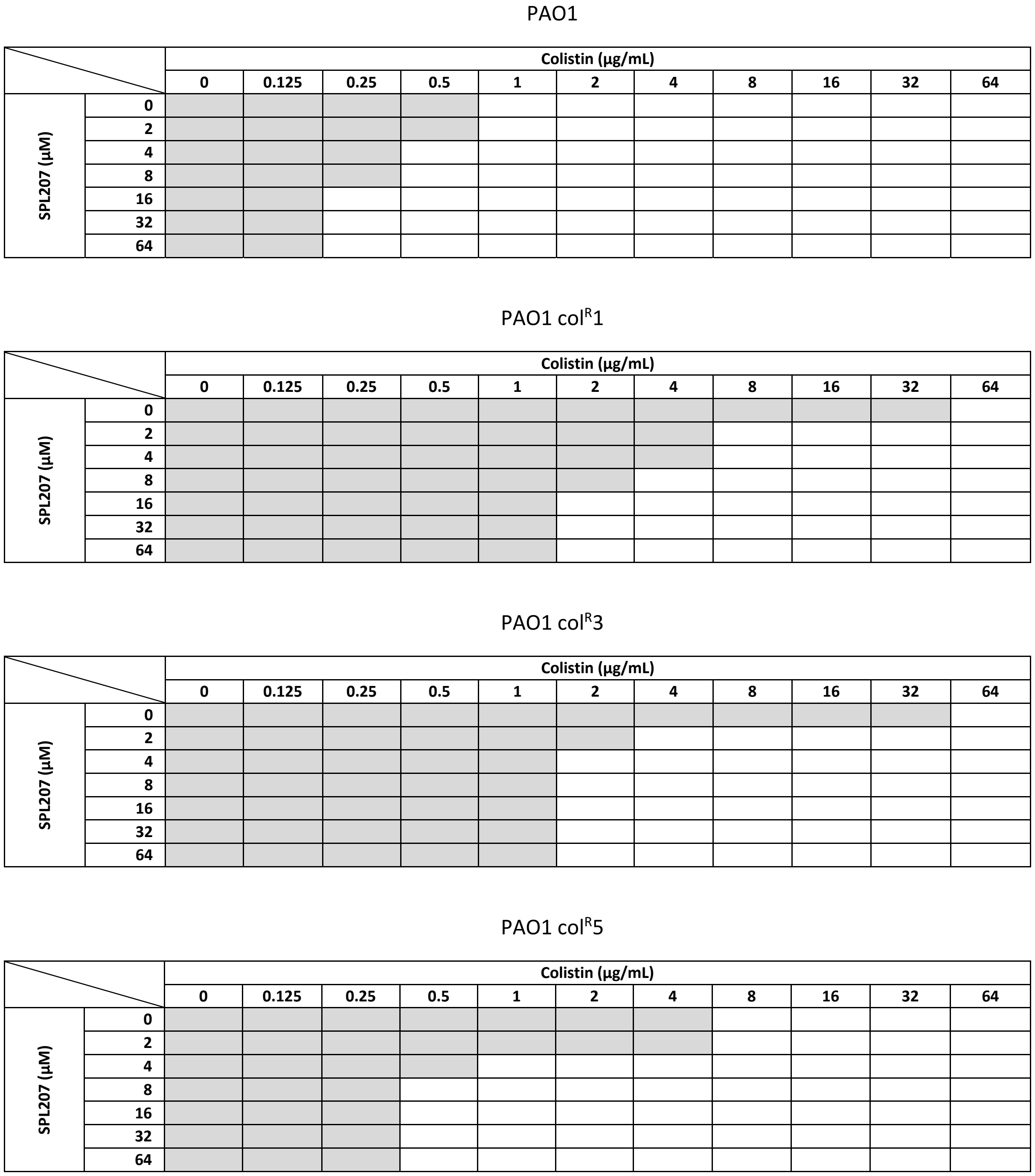

Figure S2. Checkerboard assays with SPL207 and colistin. Schematic representation of the checkerboard plates containing cultures of the indicated *P. aeruginosa* strains and different concentrations of SPL207 and/or colistin. Grey boxes indicate bacterial growth. White boxes indicate growth inhibition. Representative data from at least three independent experiments are shown.

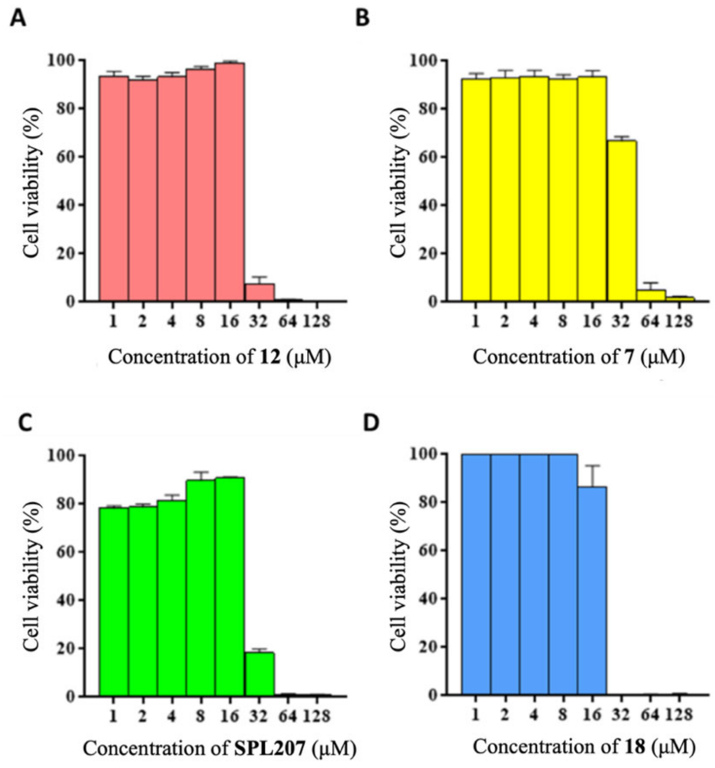

Figure S3. Cytotoxicity assays on A549 cells. Effect of compounds **12** (A), **7** (B), **SPL207** (C), **18** (D) on the viability of A549 cells, expressed as a percentage relative to control cells maintained in DMEM medium containing 1% DMSO. Each concentration was analyzed in triplicate and data are presented as the mean of three independent experiments ± standard error of the mean (SEM).

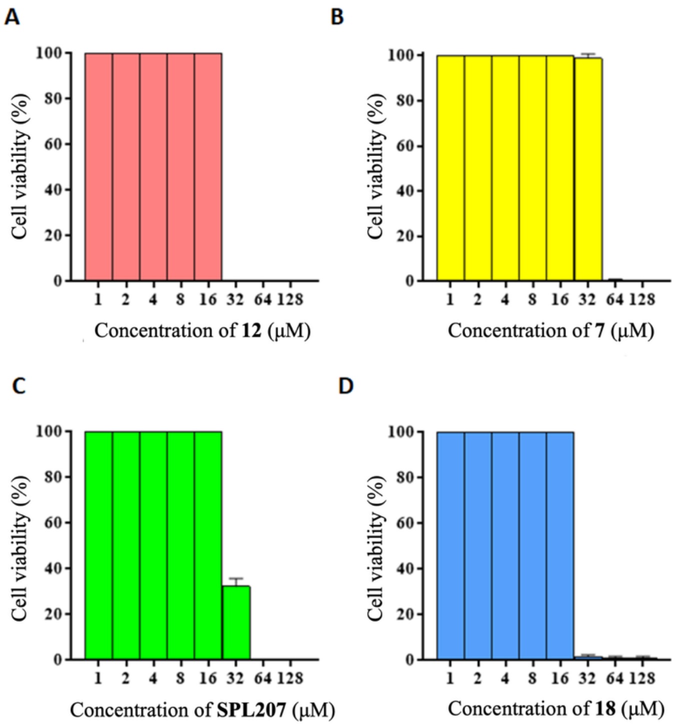

Figure S4. Cytotoxicity assays on HaCaT cells. Effect of compounds **12** (A), **7** (B), **SPL207** (C), and **18** (D) on the viability of HaCaT cells, expressed as a percentage relative to control cells maintained in DMEM medium containing 1% DMSO. Each concentration was analyzed in triplicate and data are presented as the mean of three independent experiments ± standard error of the mean (SEM).

^{1}H-NMR (400 MHz, DMSO-d_6) compound **7**.

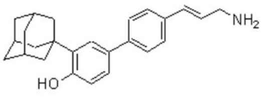

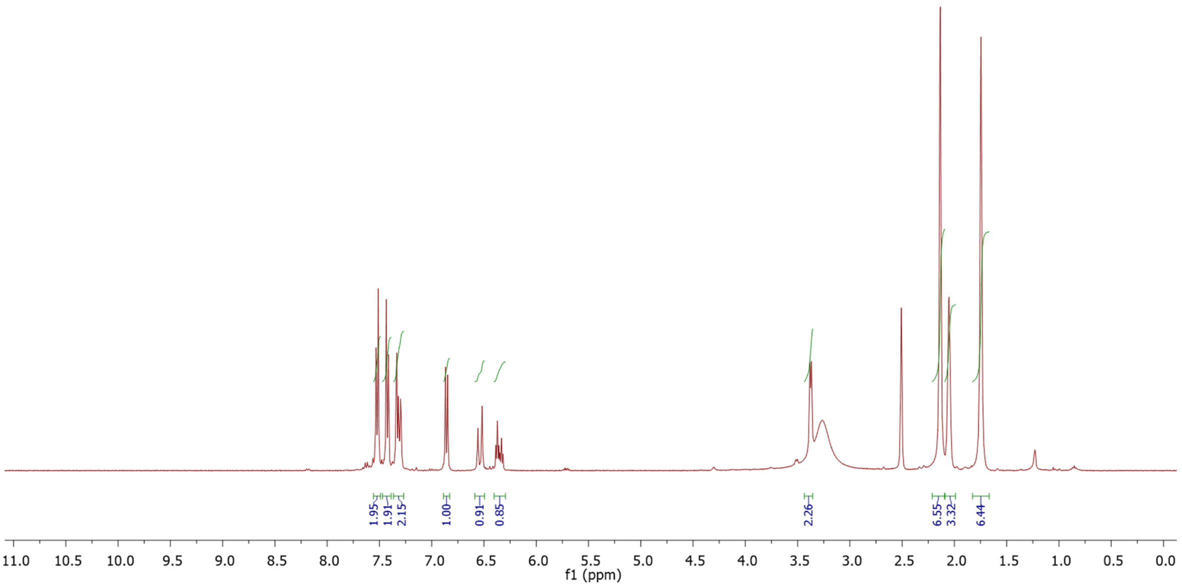

^{13}C-NMR (100 MHz, DMSO-d_6) compound **7**.

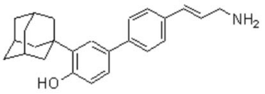

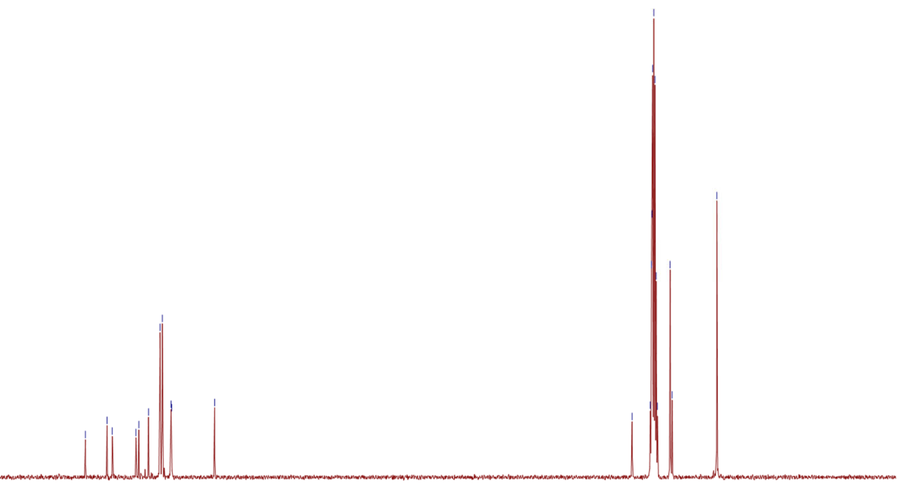

^{1}H-NMR (400 MHz, CH_3OH-*d*$_4$) compound **8**.

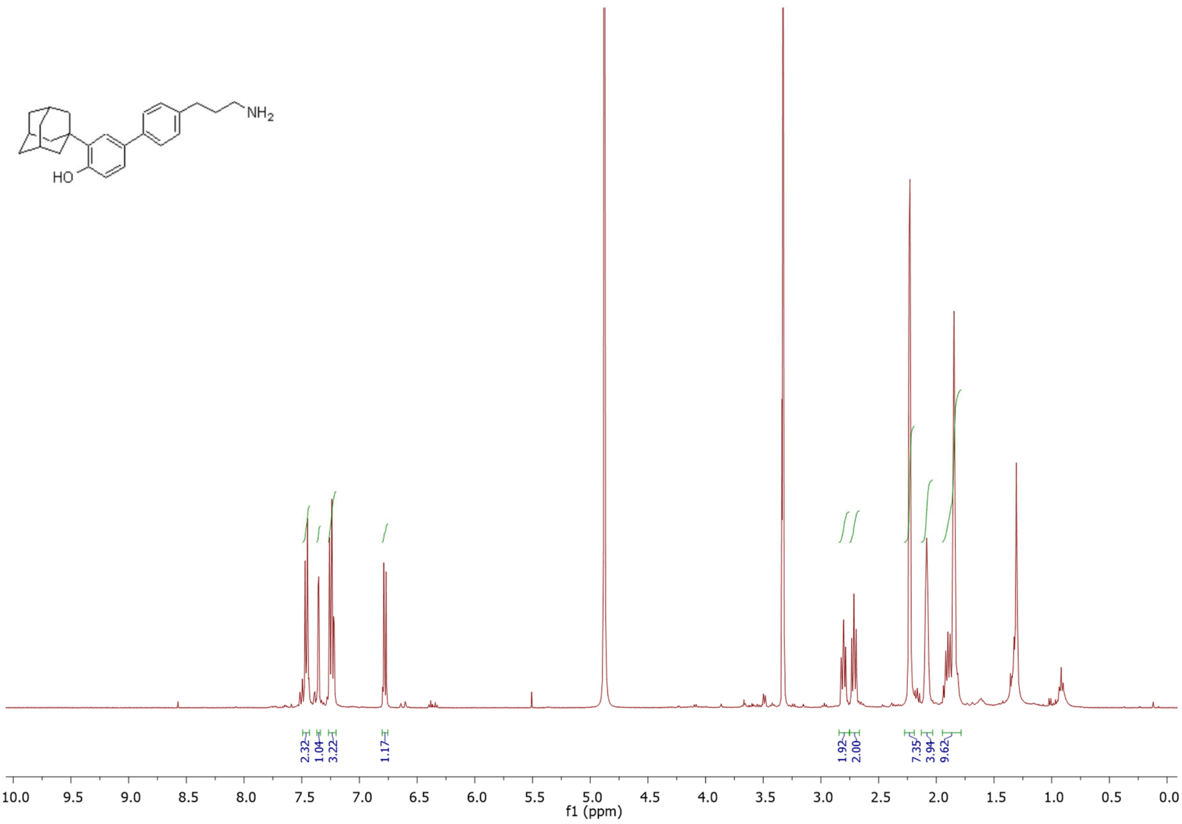

^{13}C-NMR (100 MHz, CH_3OH-*d*$_4$) compound **8**.

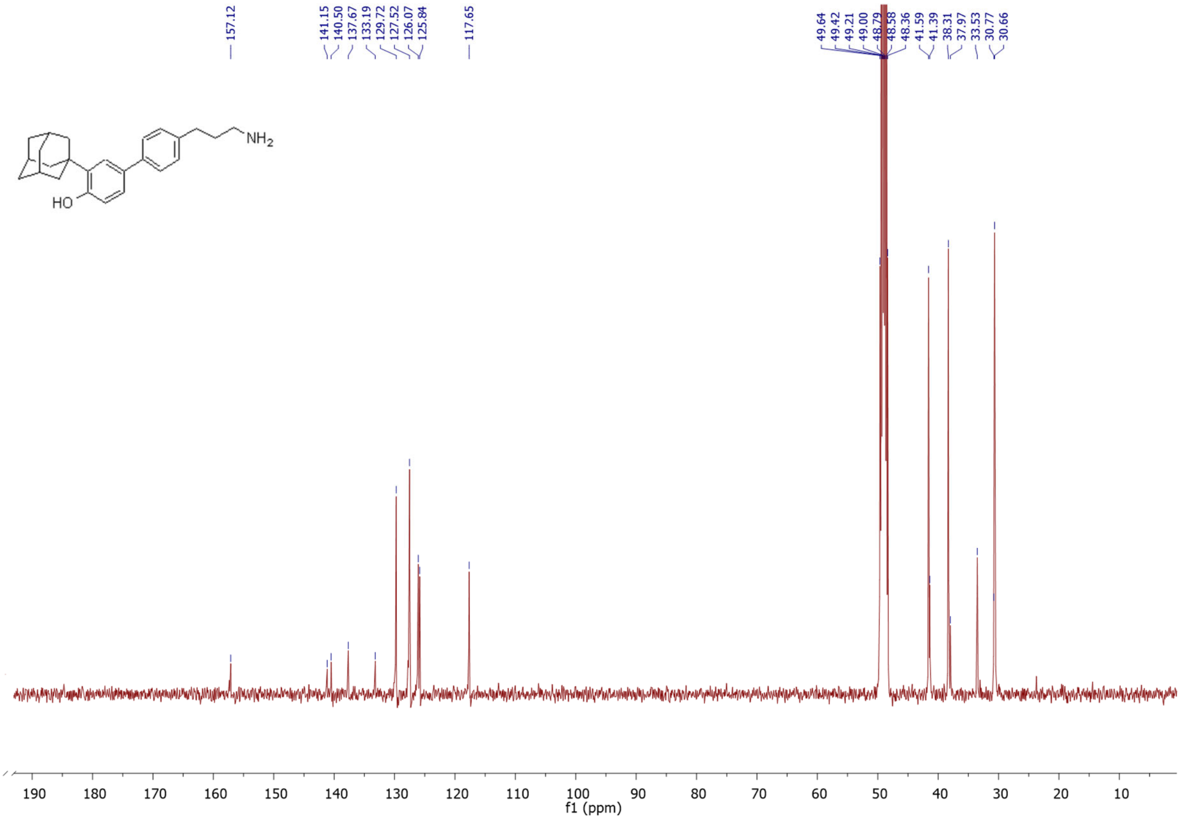

^{1}H-NMR (400 MHz, DMSO-d_6) compound **9**.

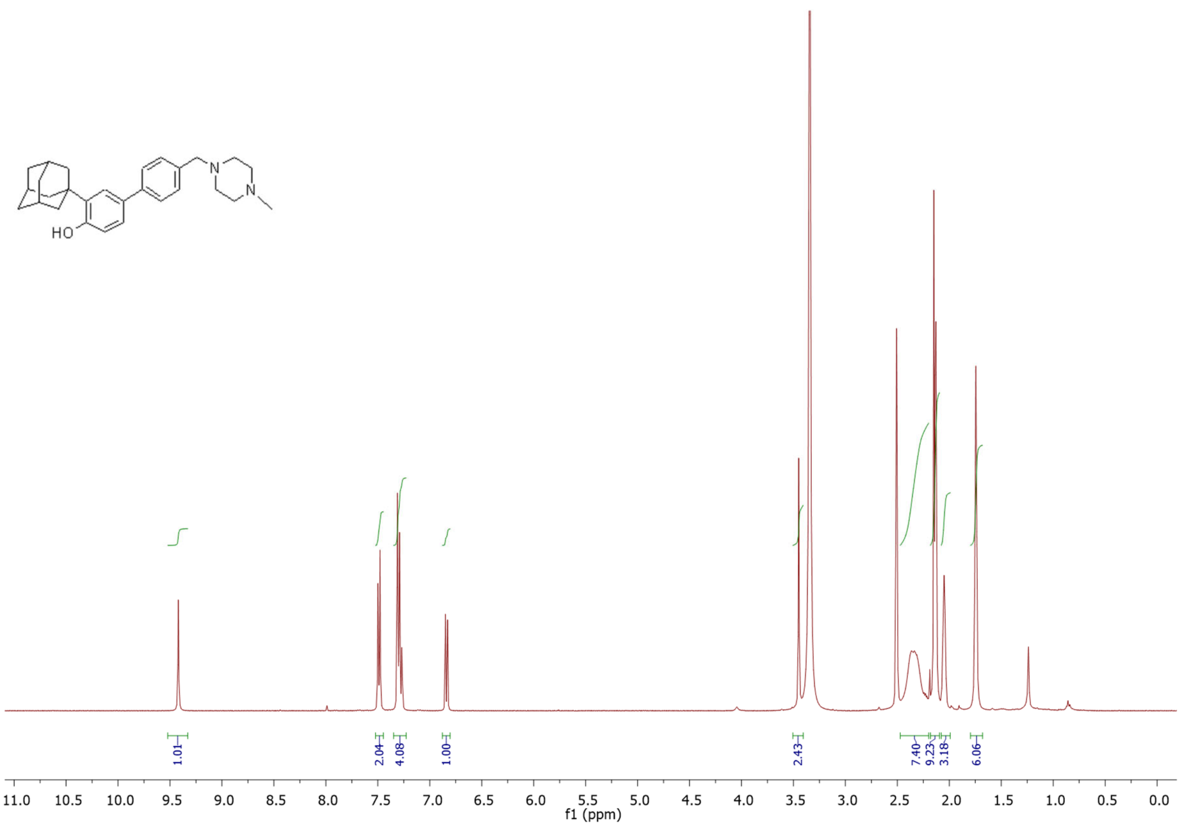

^{13}C-NMR (100 MHz, DMSO-d_6) compound **9**.

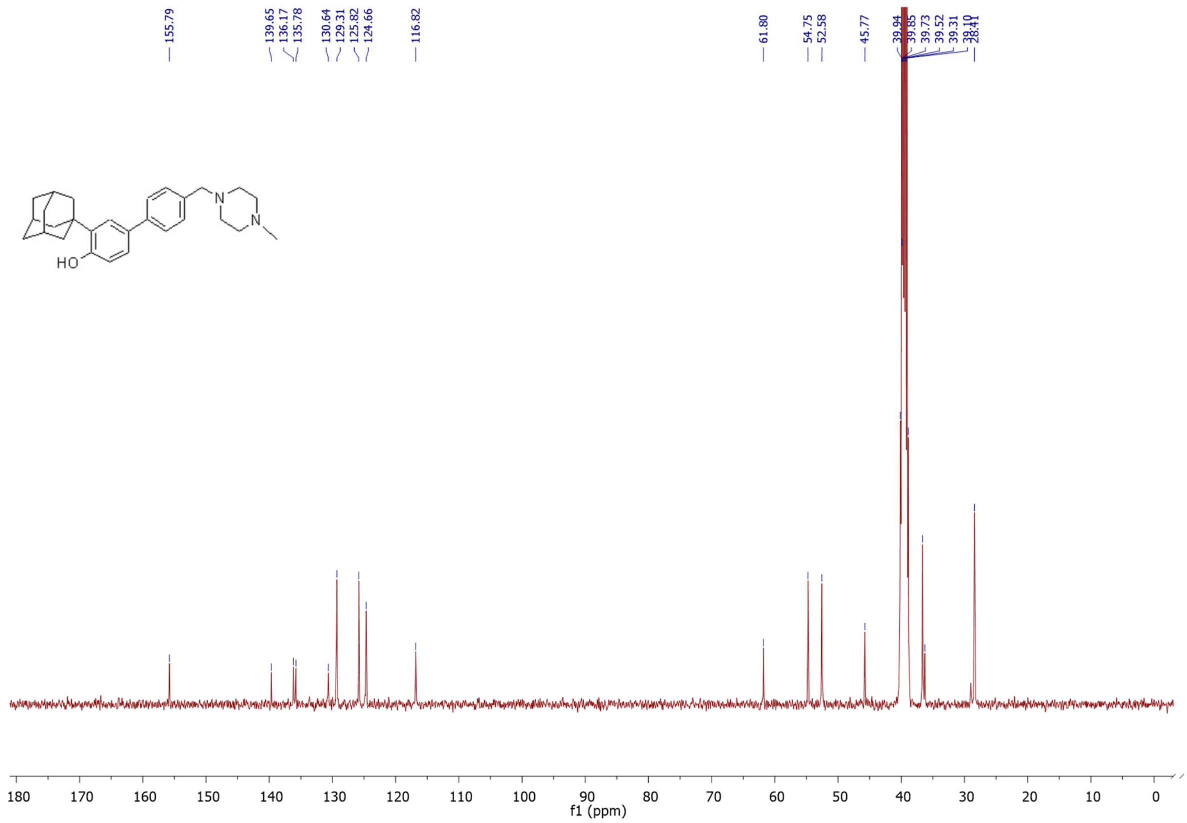

^{1}H-NMR (400 MHz, DMSO-d_6) compound **10**.

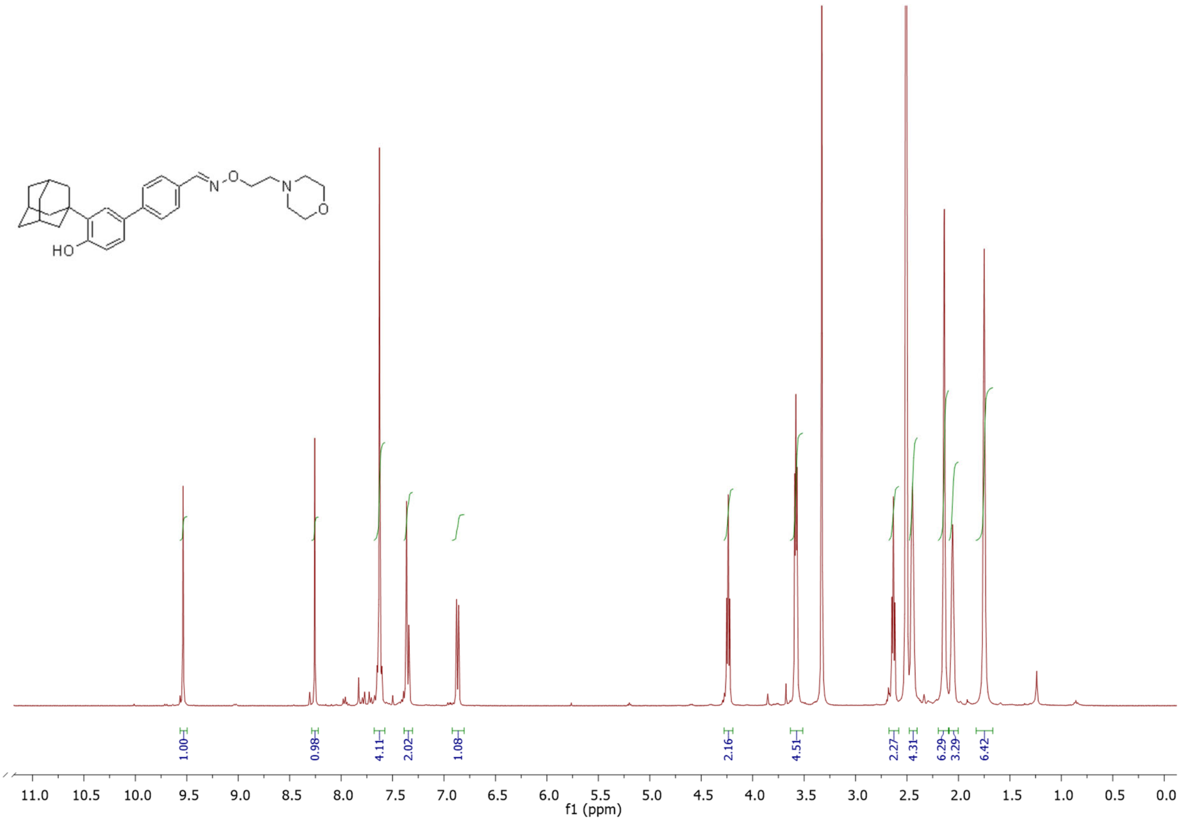

^{13}C-NMR (100 MHz, DMSO-d_6) compound **10**.

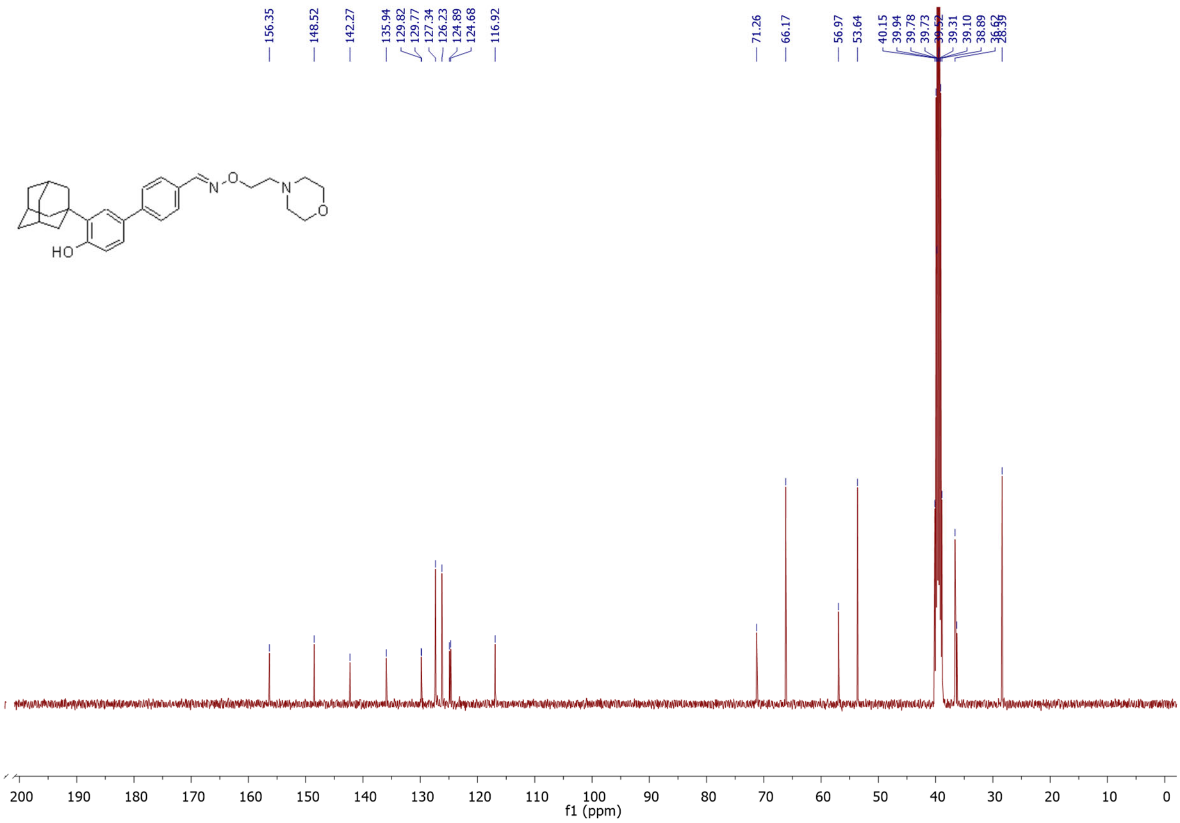

¹H-NMR (600 MHz, CH_3OH-*d*$_4$) compound **11**.

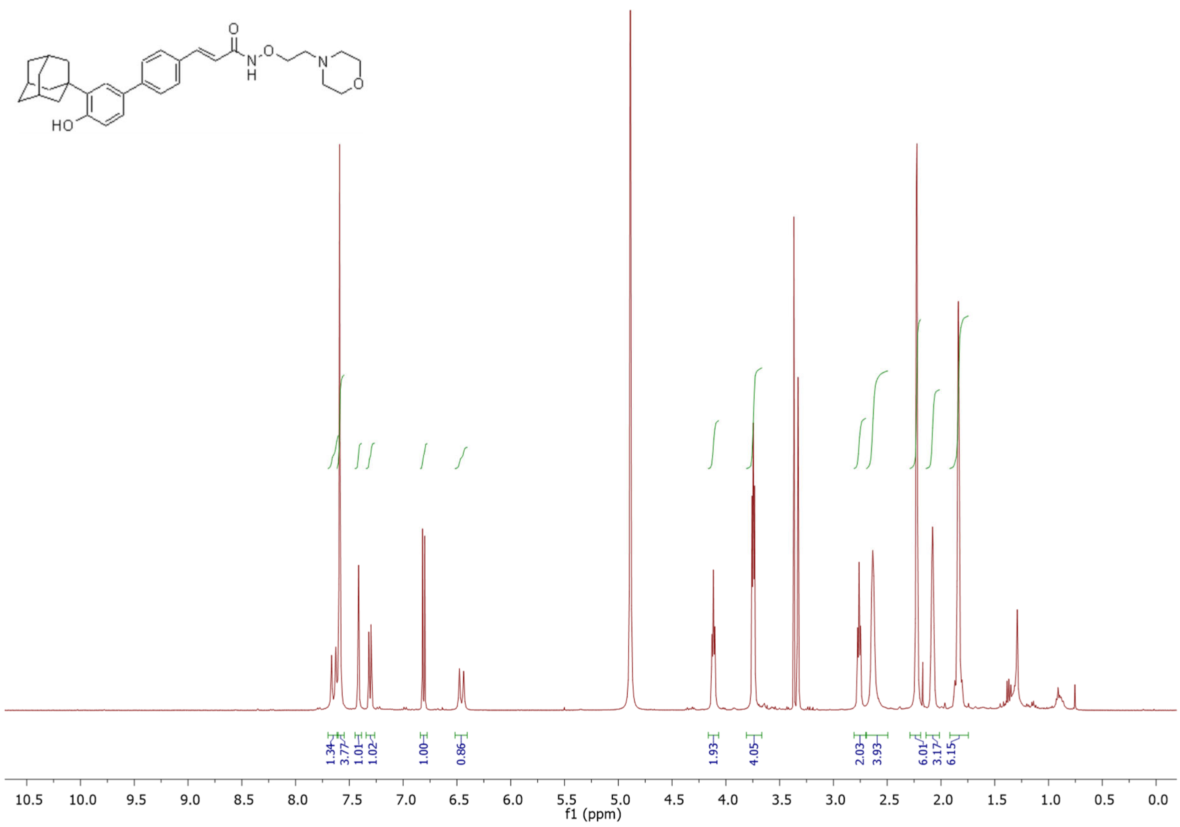

¹³C-NMR (150 MHz, CH_3OH-*d*$_4$) compound **11**.

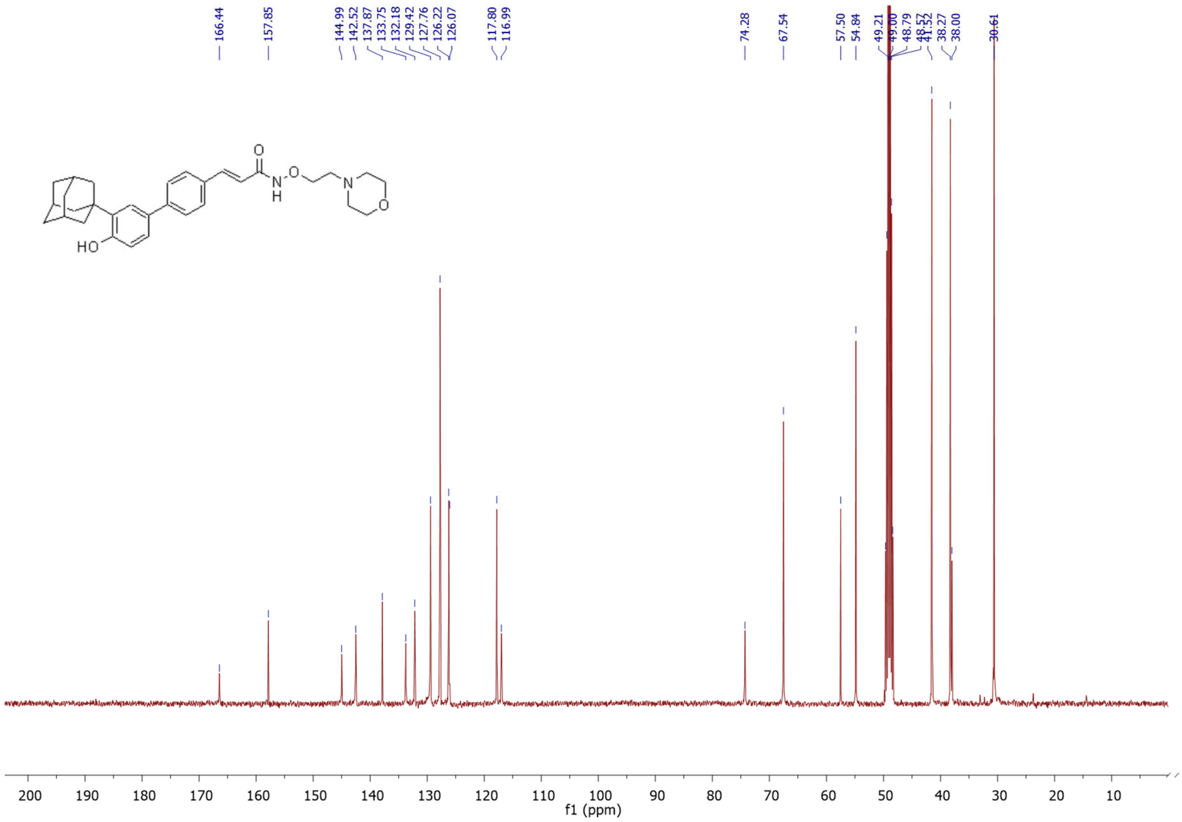

^{1}H-NMR (600 MHz, CH_3OH-*d*$_4$) compound **12**.

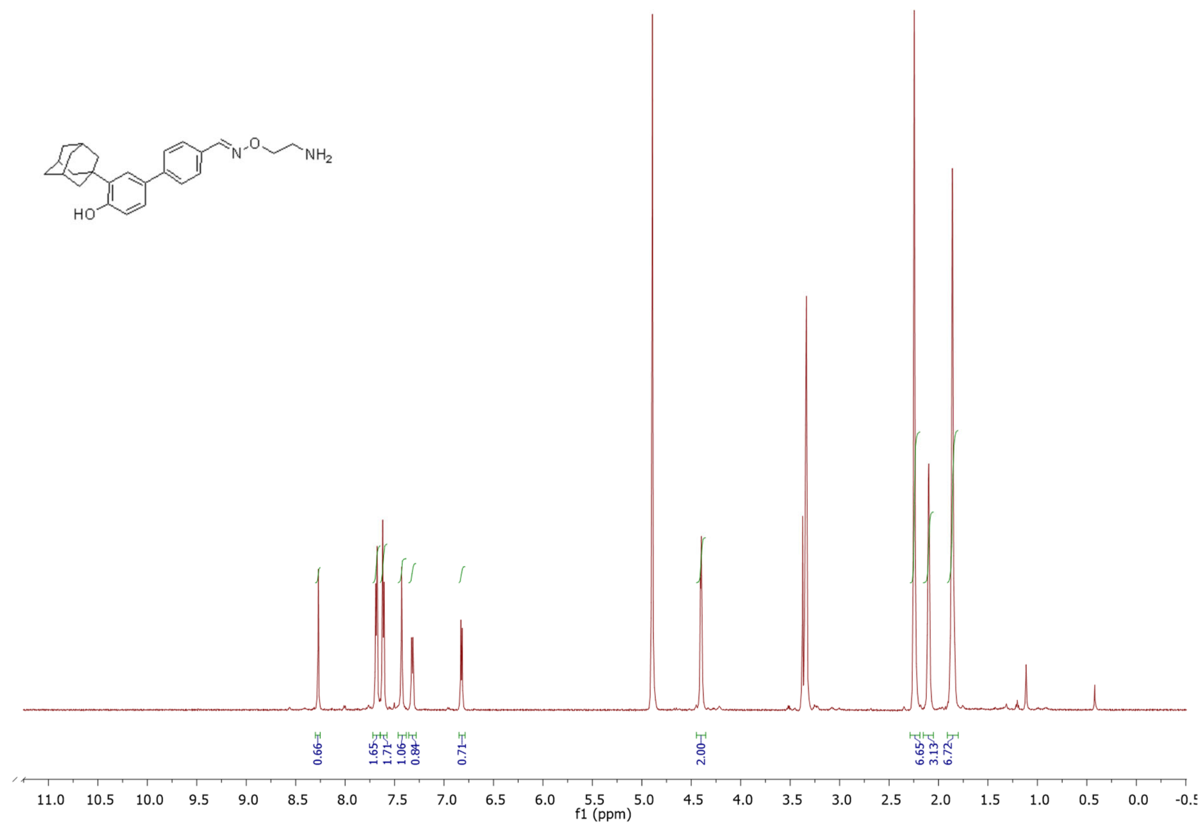

^{13}C-NMR (150 MHz, CH_3OH-*d*$_4$) compound **12**.

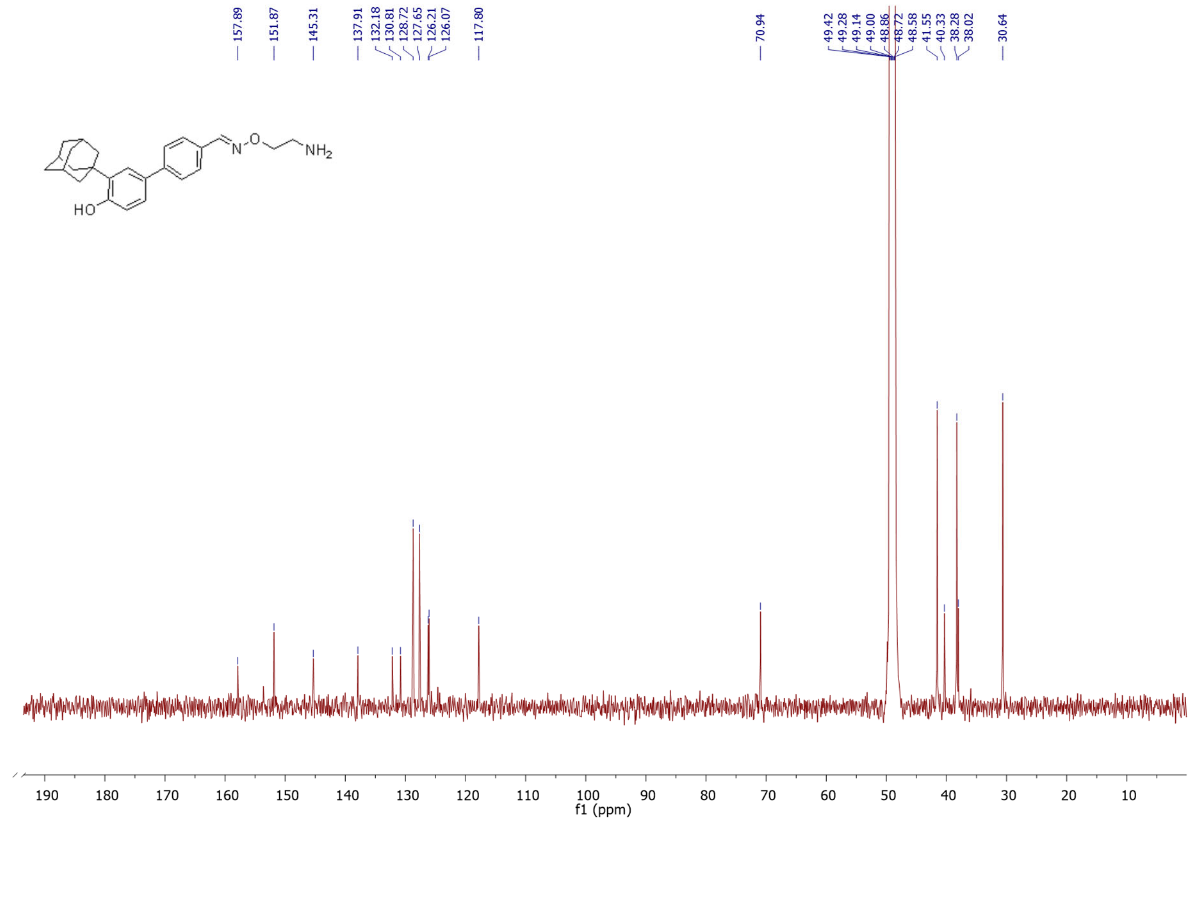

^{1}H-NMR (400 MHz, CH_3OH-d_4) compound **13**.

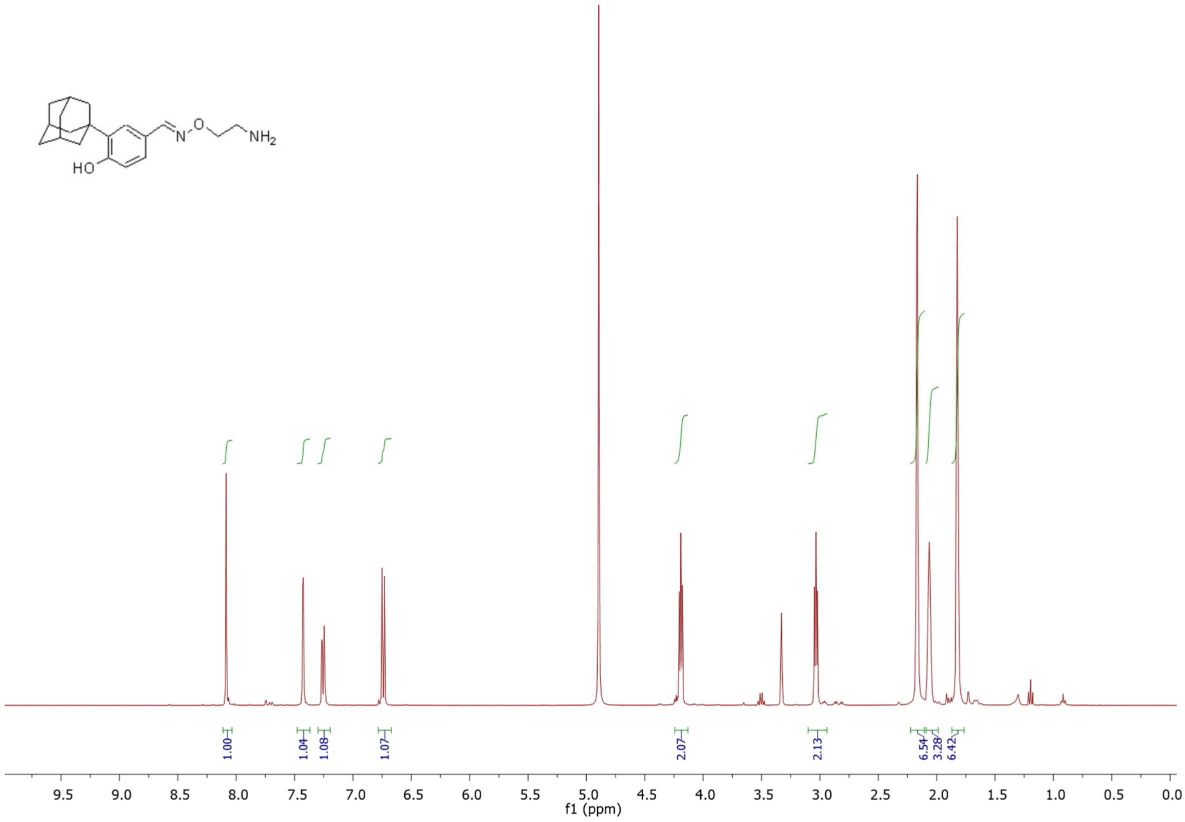

^{13}C-NMR (100 MHz, CH_3OH-d_4) compound **13**.

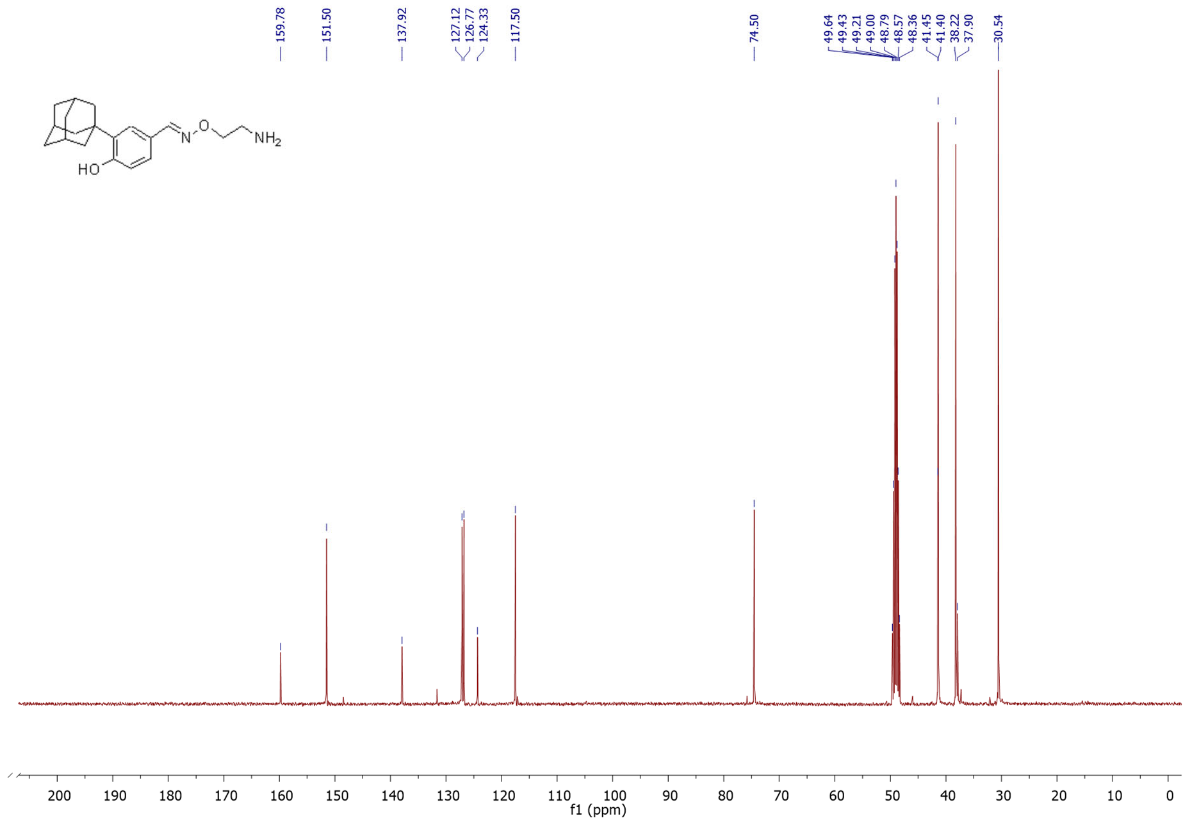

^{1}H-NMR (600 MHz, CH_3OH-*d*$_4$) compound **14**.

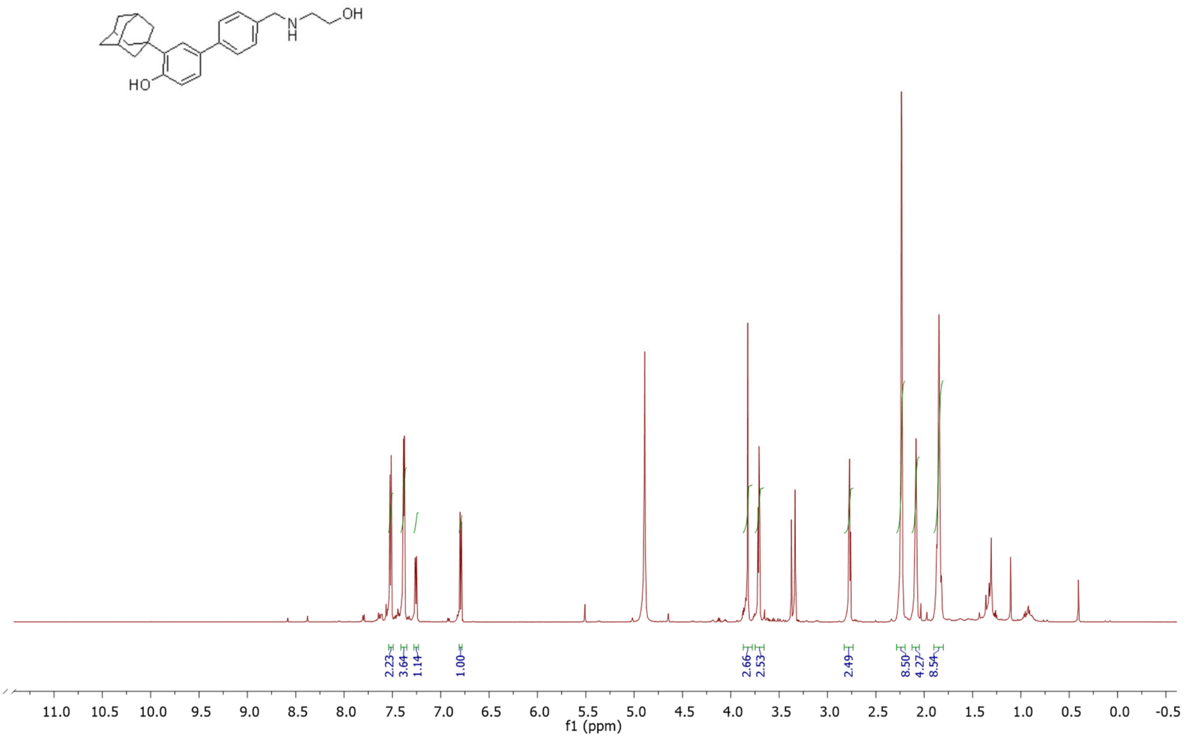

^{13}C-NMR (150 MHz, CH_3OH-*d*$_4$) compound **14**.

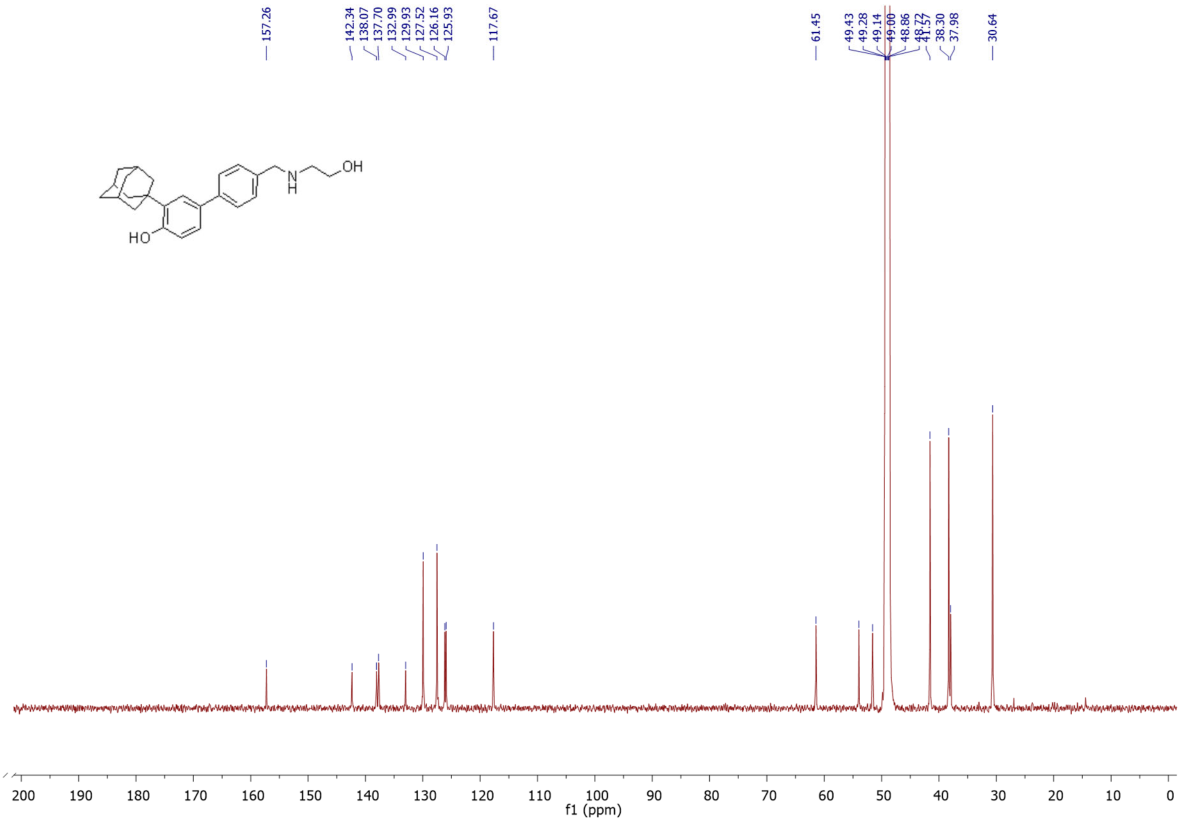

^{1}H-NMR (600 MHz, CH_3OH-*d*$_4$) compound **15**.

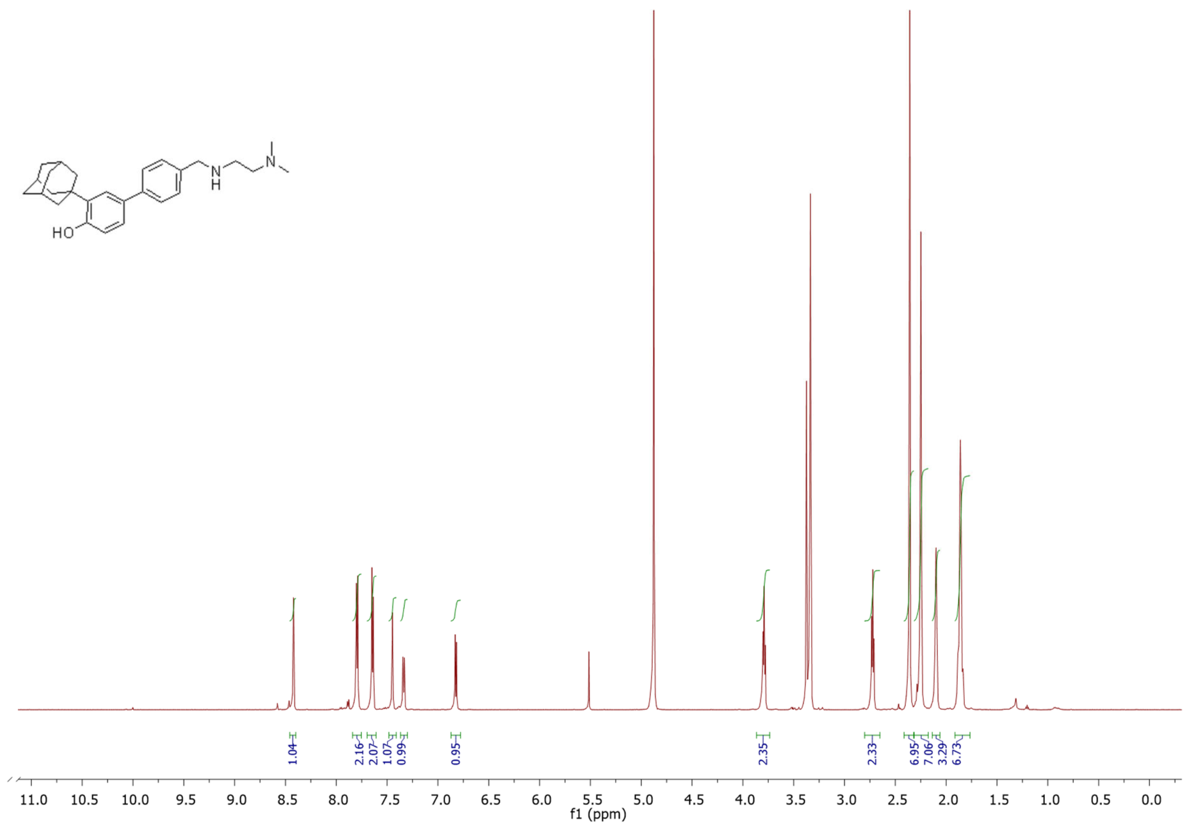

^{13}C-NMR (150 MHz, CH_3OH-*d*$_4$) compound **15**.

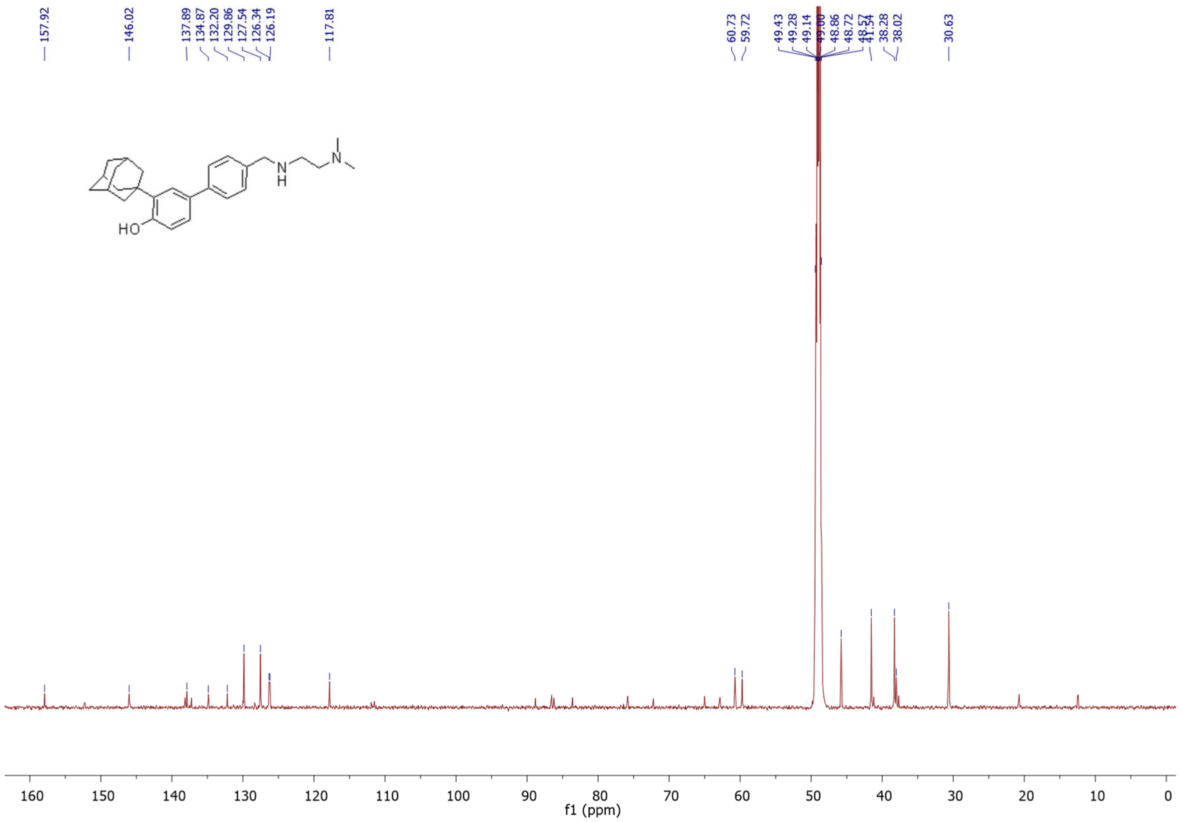

^{1}H-NMR (400 MHz, CH_3OH-*d₄*) compound **16**.

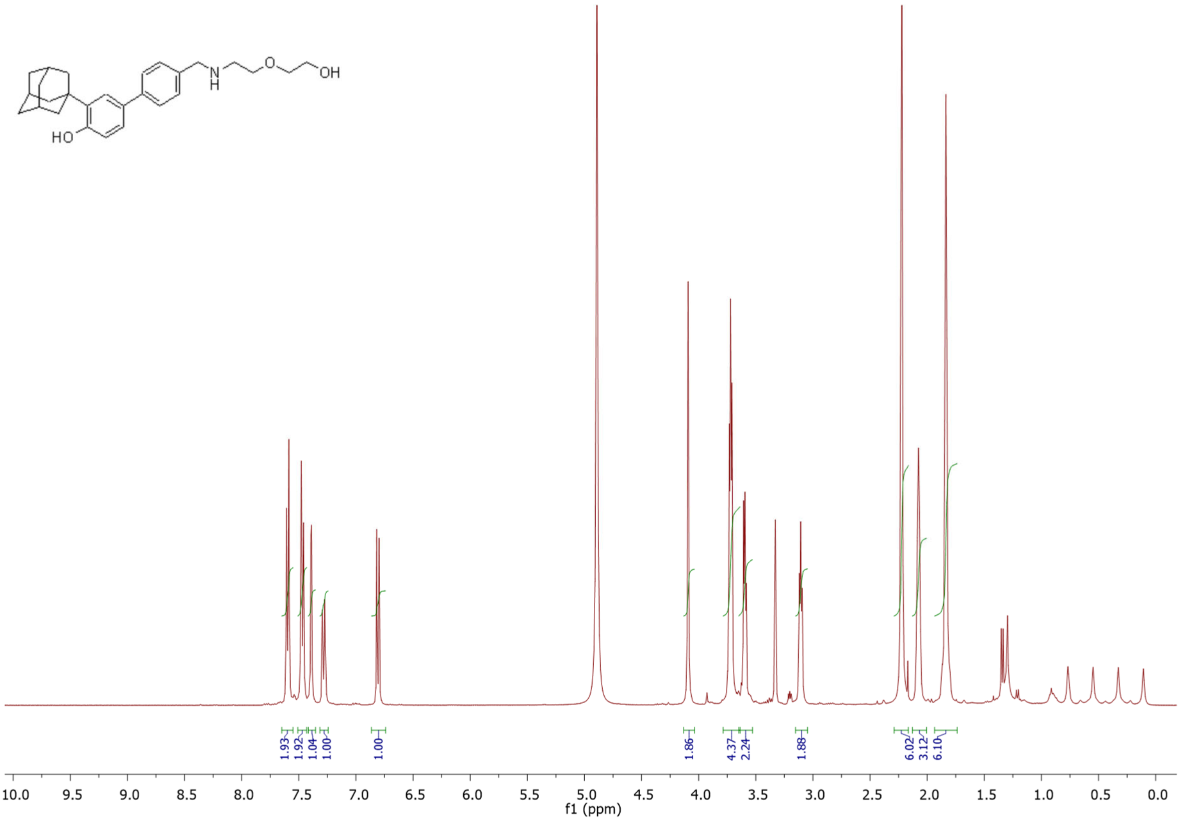

^{13}C-NMR (150 MHz, CH_3OH-*d₄*) compound **16**.

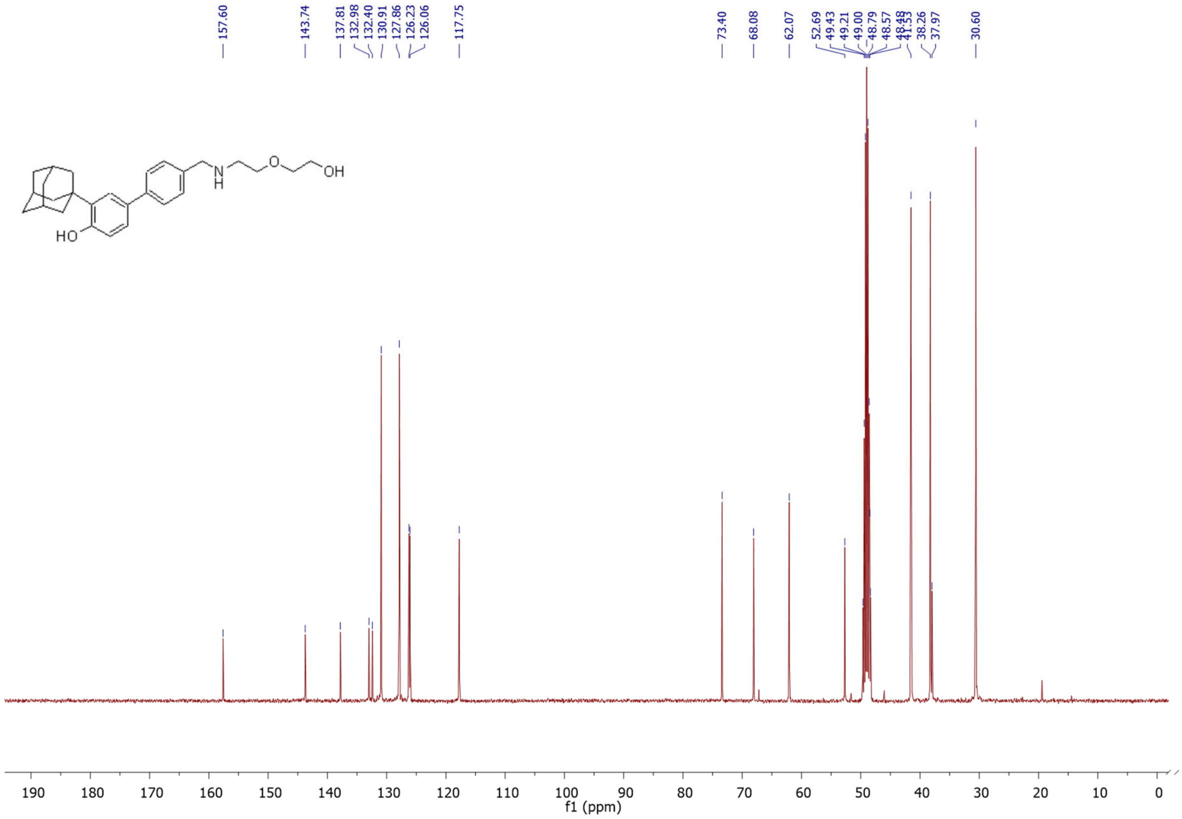

^{1}H-NMR (400 MHz, CH_3OH-d_4) compound **17**.

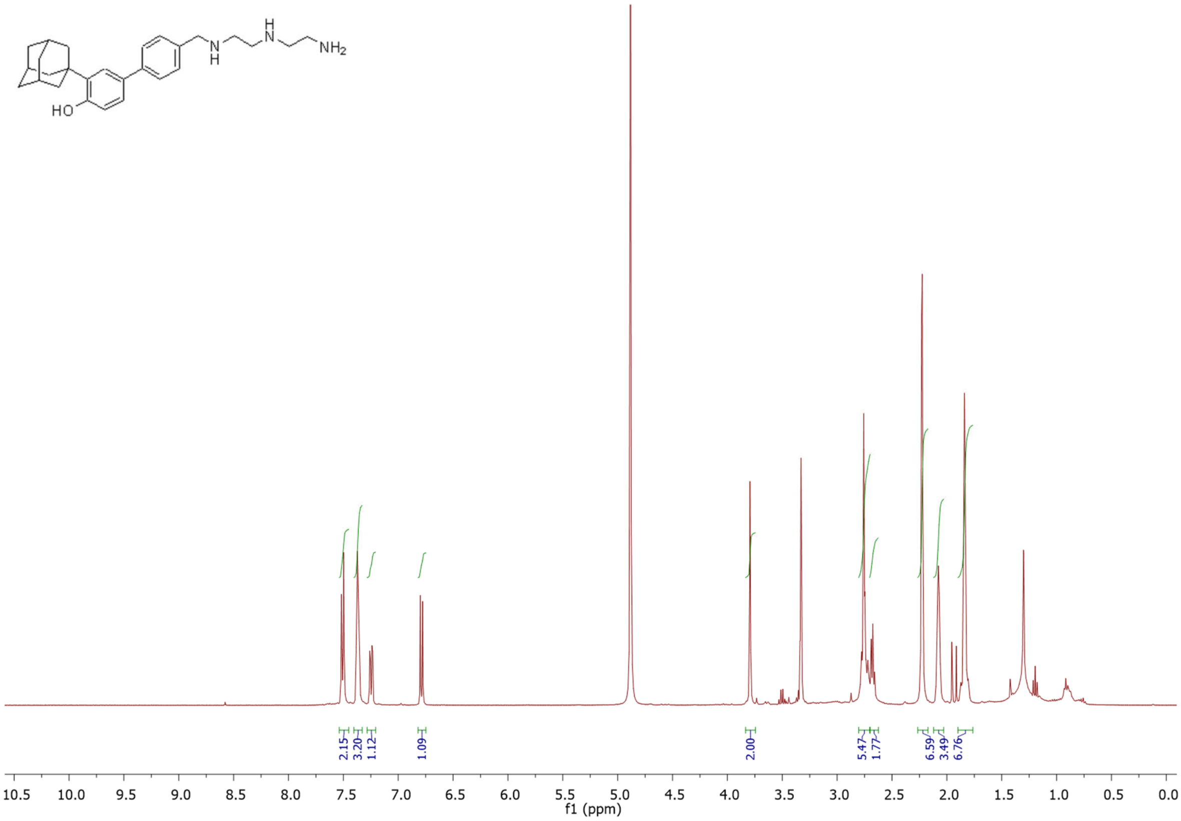

^{13}C-NMR (100 MHz, CH_3OH-d_4) compound **17**.

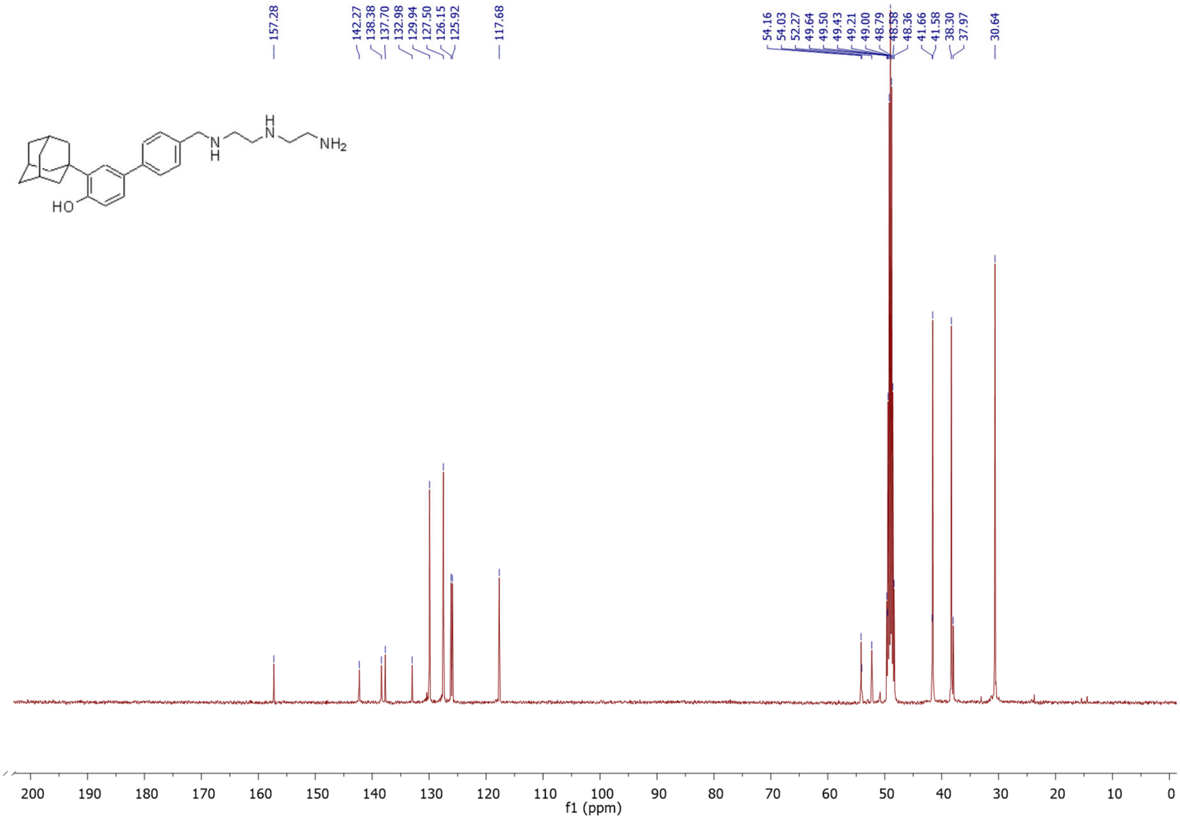

^{1}H-NMR (600 MHz, CH_3OH-*d*$_4$) compound **18**.

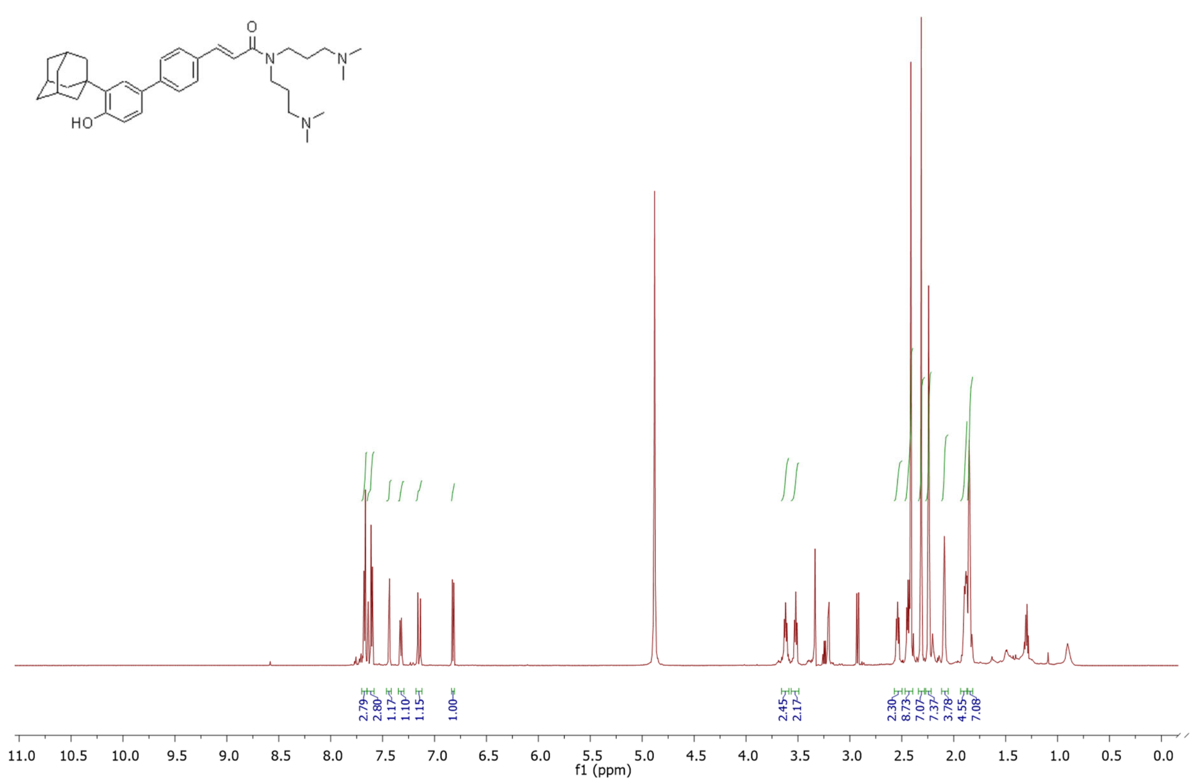

^{13}C-NMR (150 MHz, CH_3OH-*d*$_4$) compound **18**.

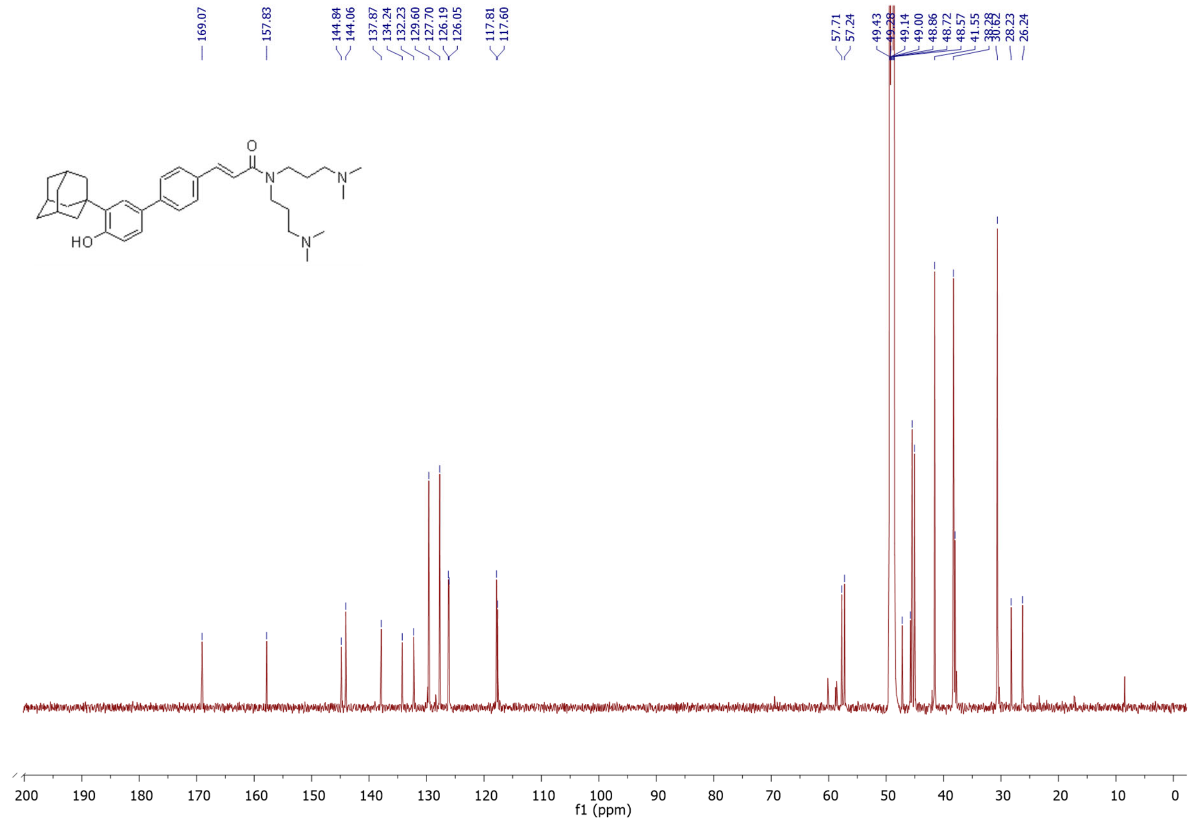

^{1}H-NMR (600 MHz, CH_3OH-d_4) compound **19**.

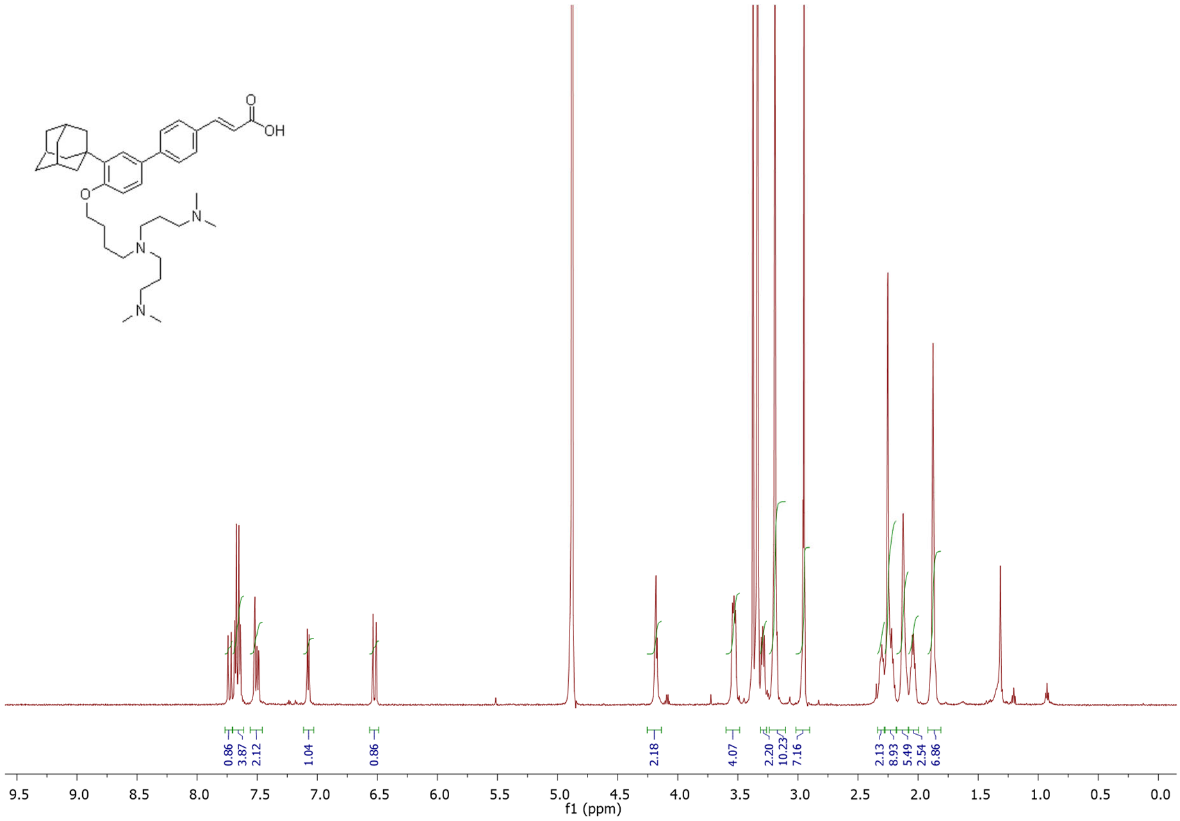

^{13}C-NMR (150 MHz, CH_3OH-d_4) compound **19**.

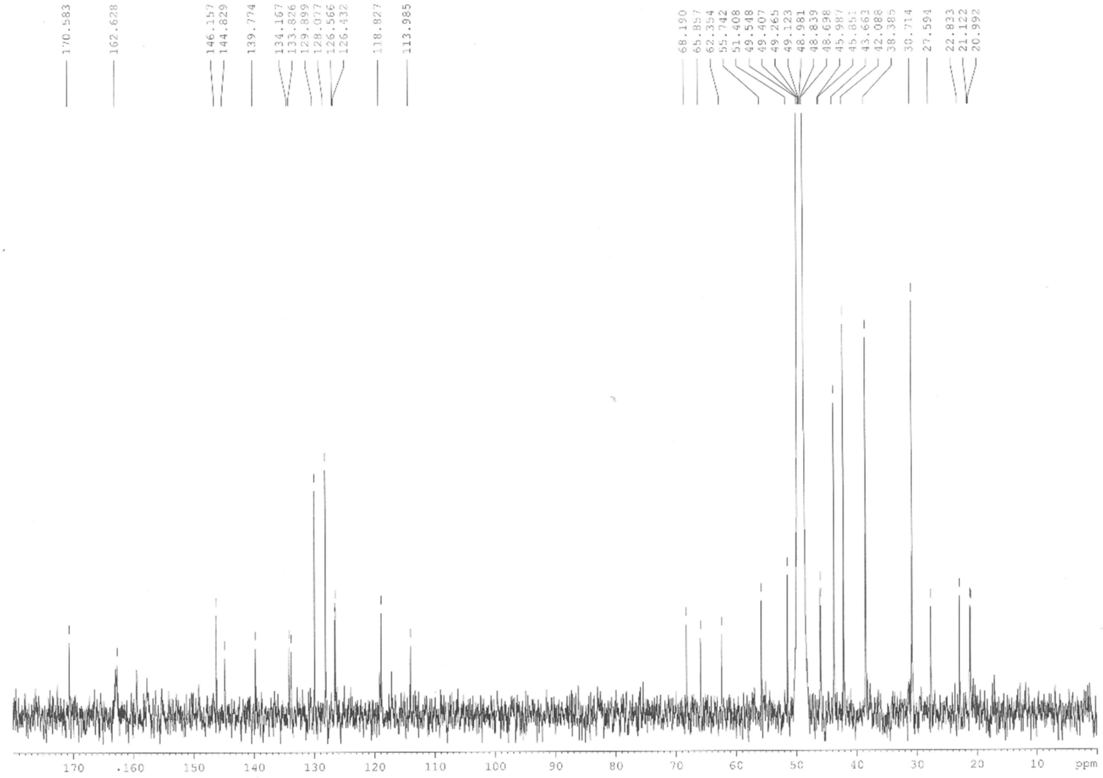

^{1}H-NMR (600 MHz, CH_3OH-*d*$_4$) compound **20**.

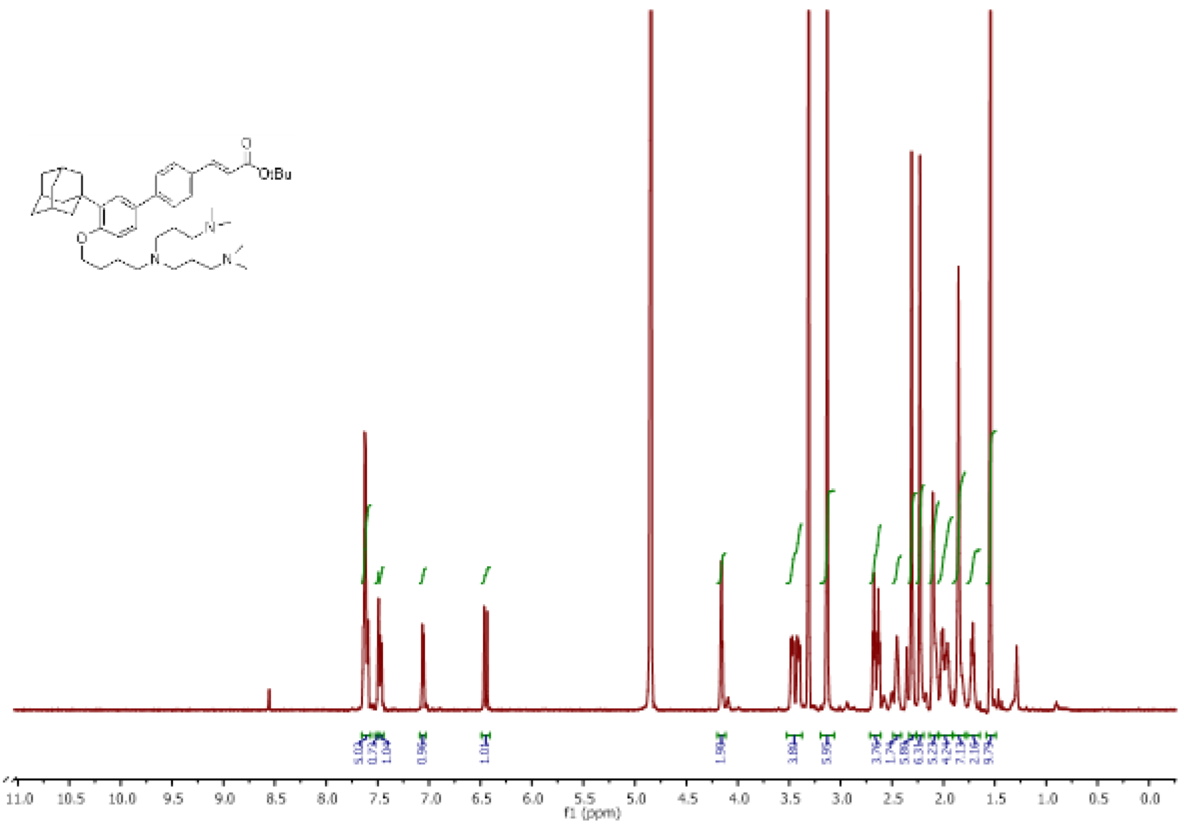

^{13}C-NMR (150 MHz, CH_3OH-*d*$_4$) compound **20**.

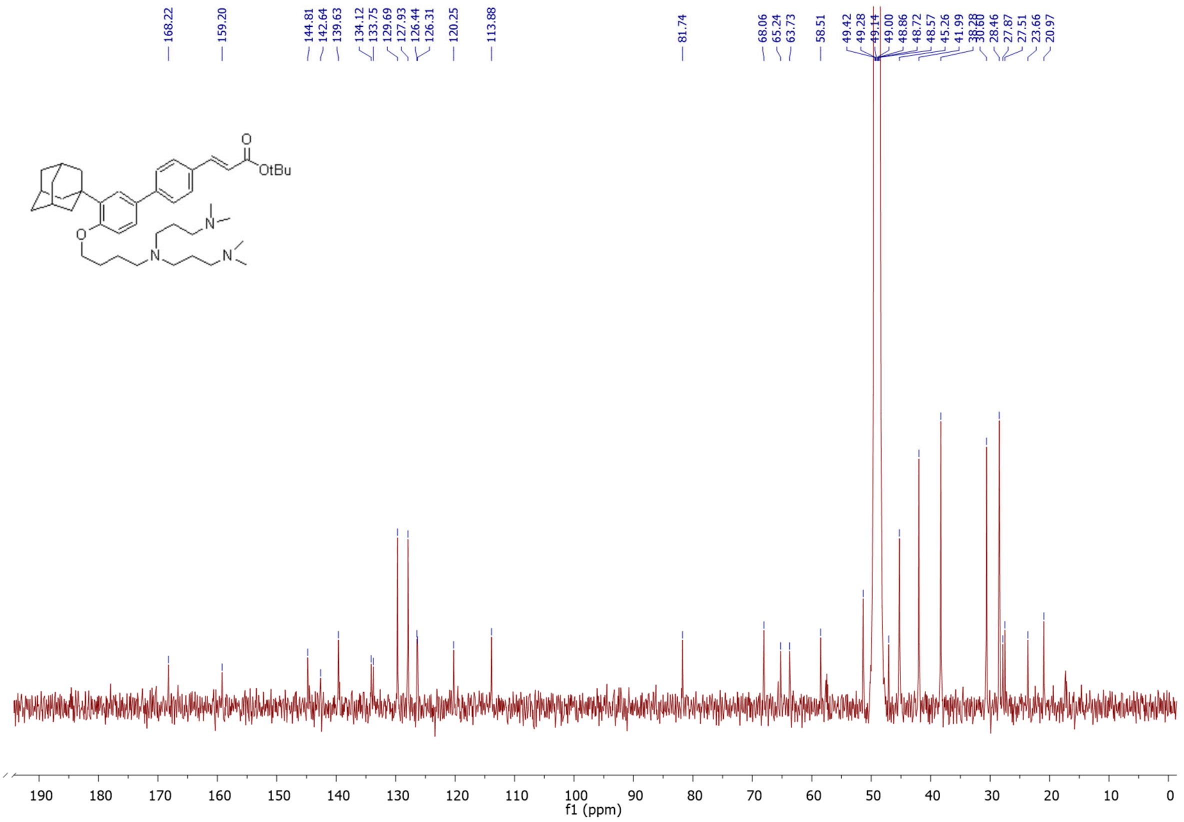

^{1}H-NMR (400 MHz, $CDCl_3$) compound **21**.

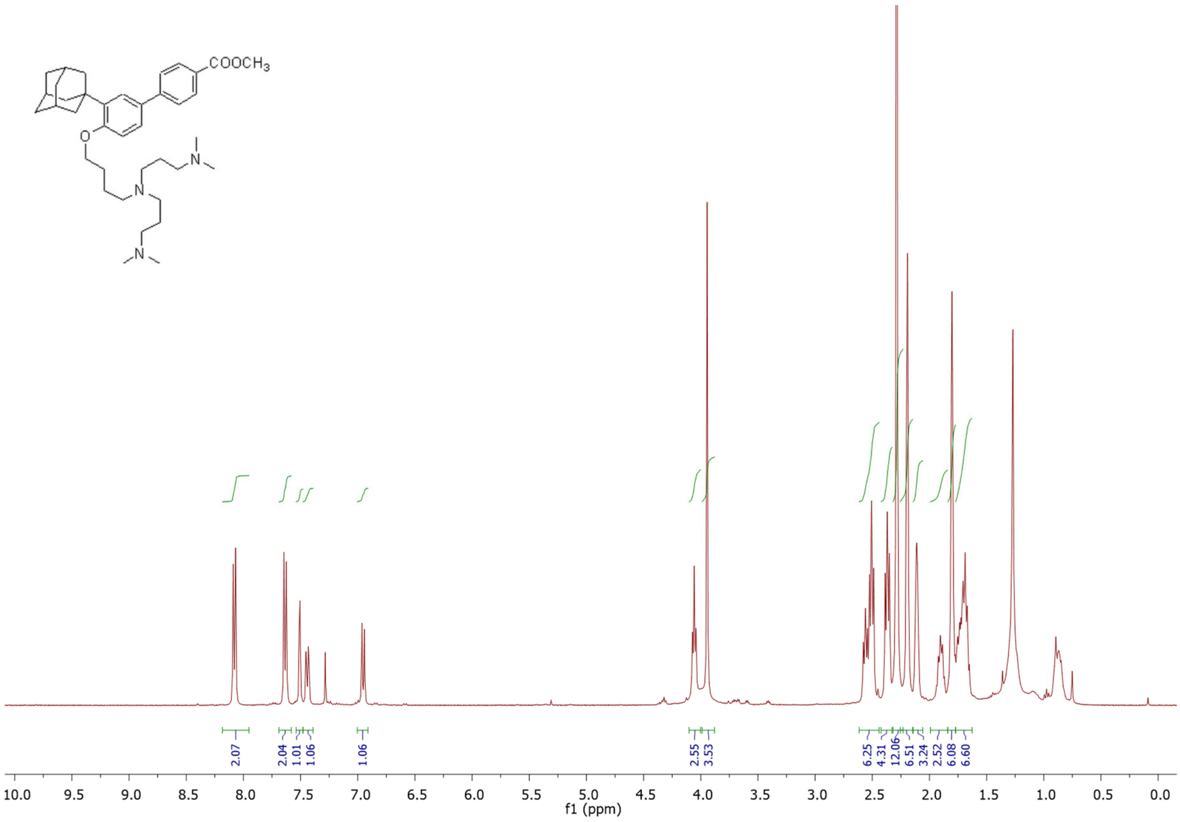

^{13}C-NMR (100 MHz, $CDCl_3$) compound **21**.

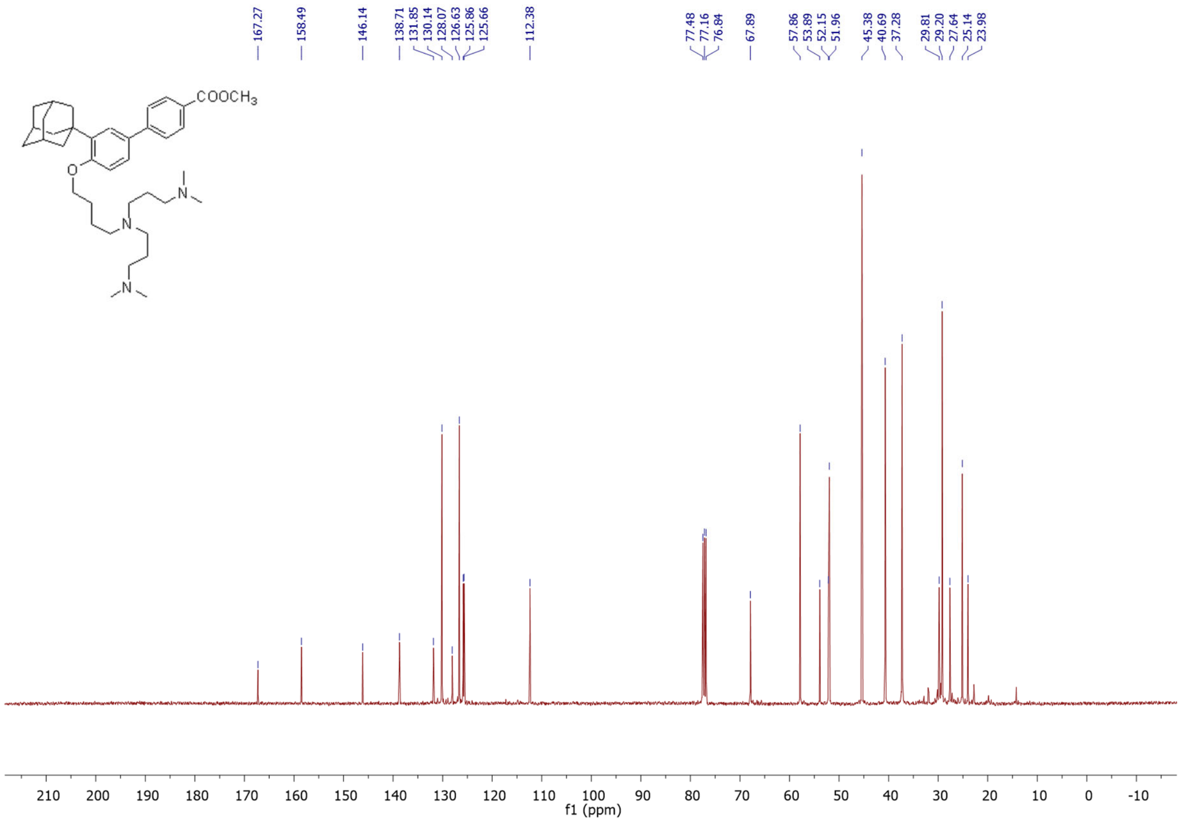

^{1}H-NMR (600 MHz, $CDCl_3$) compound **22**.

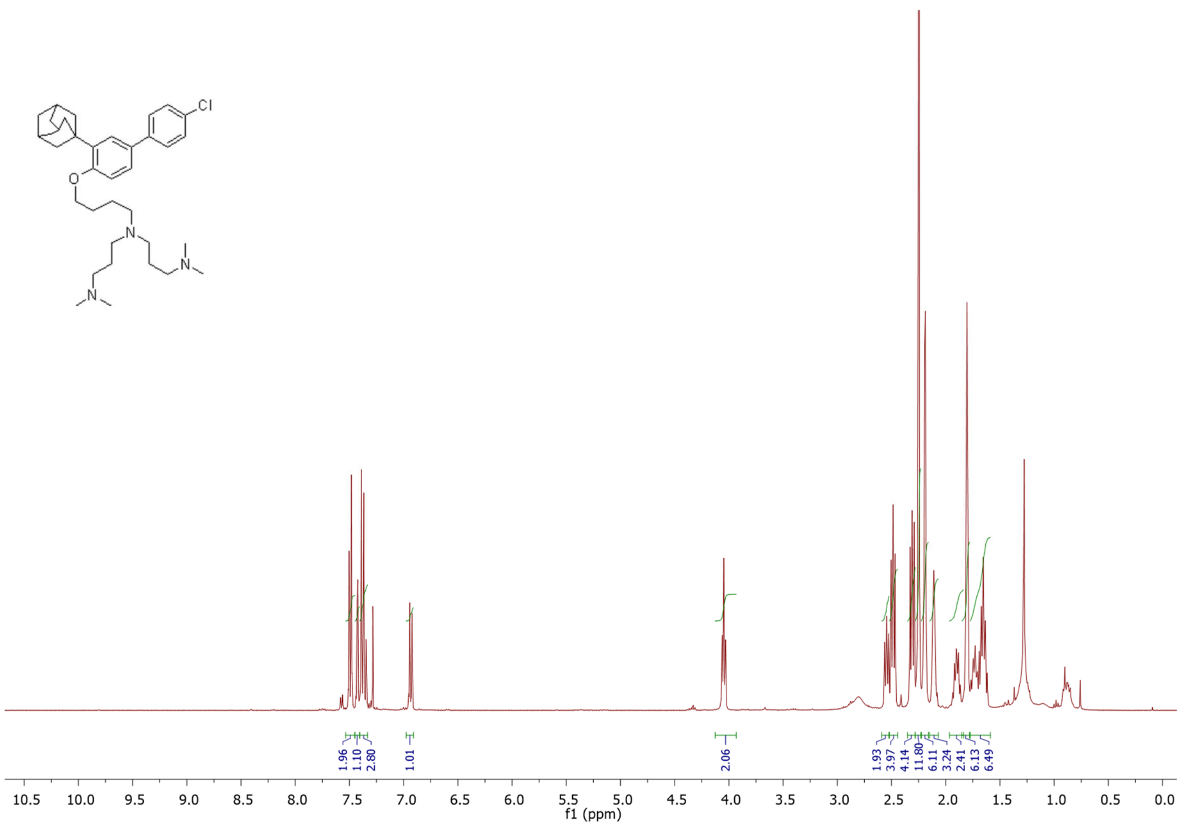

^{13}C-NMR (150 MHz, $CDCl_3$) compound **22**.

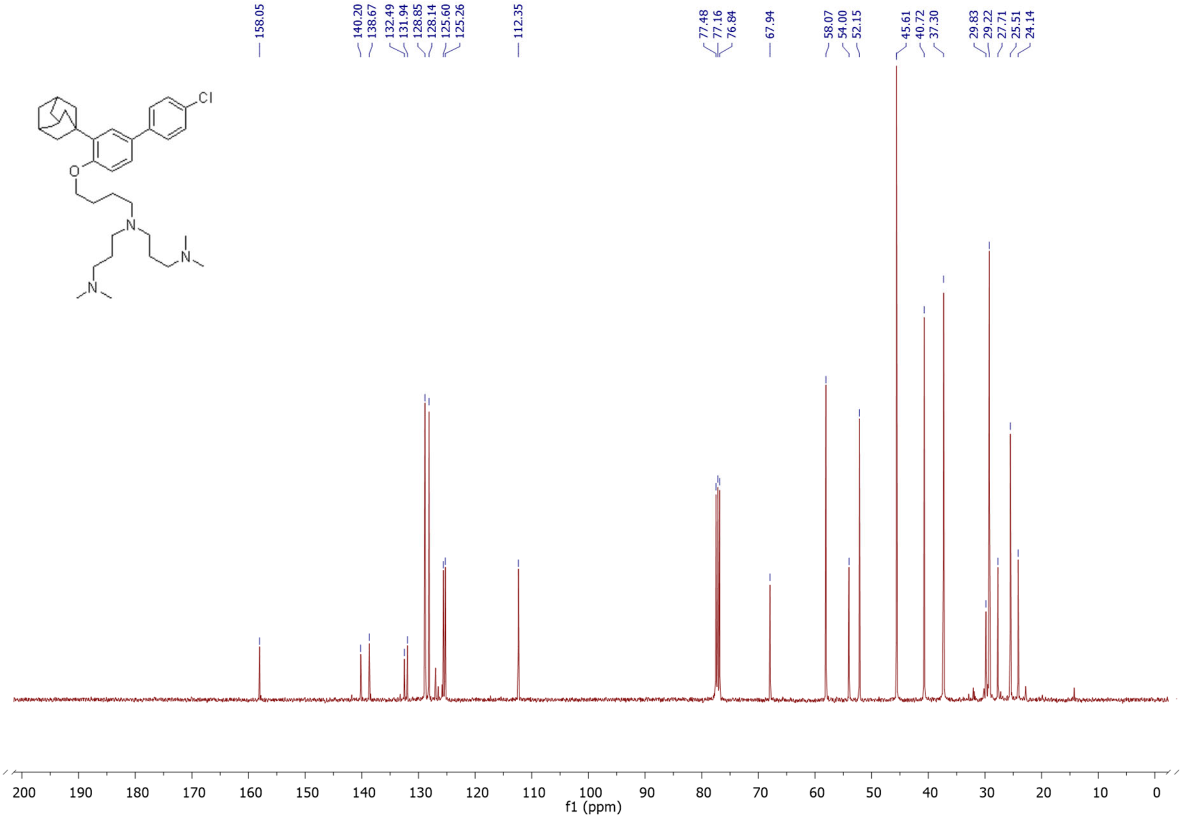

^{1}H-NMR (600 MHz, CH_3OH-d_4) compound **23**.

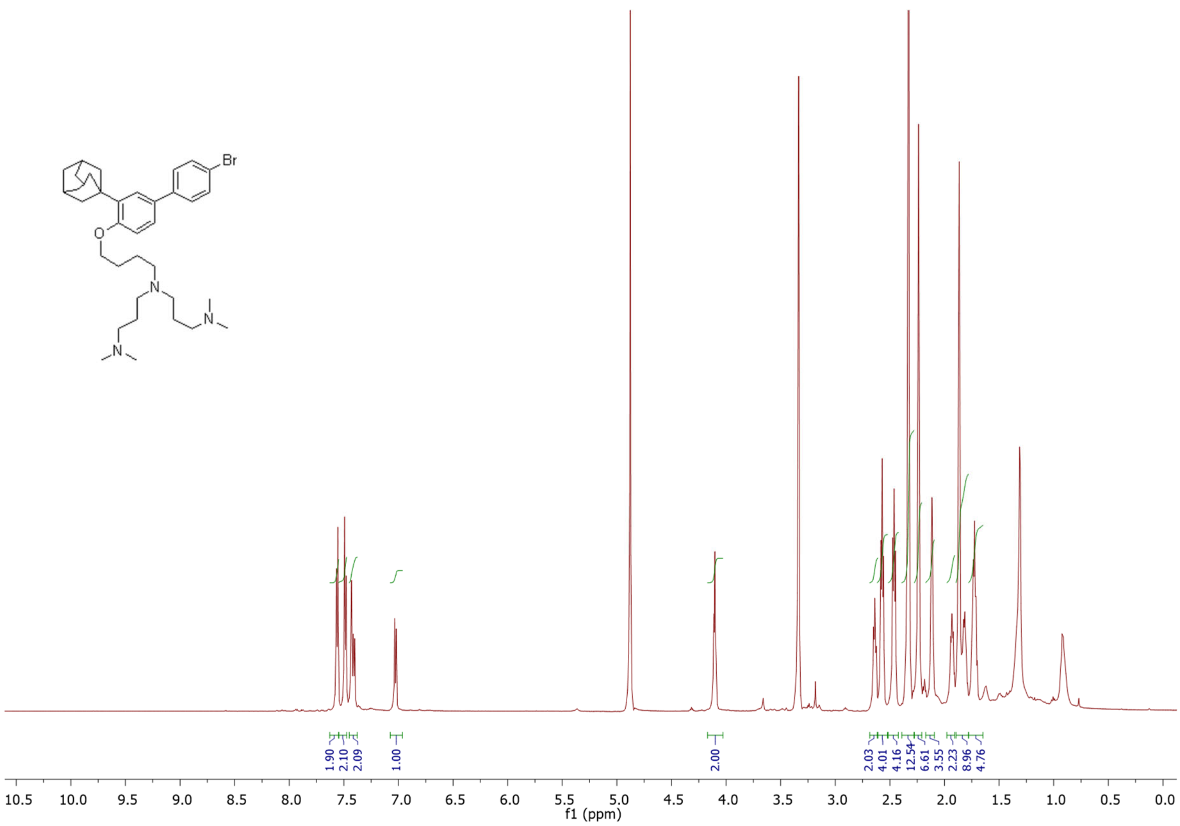

^{13}C-NMR (150 MHz, CH_3OH-d_4) compound **23**.

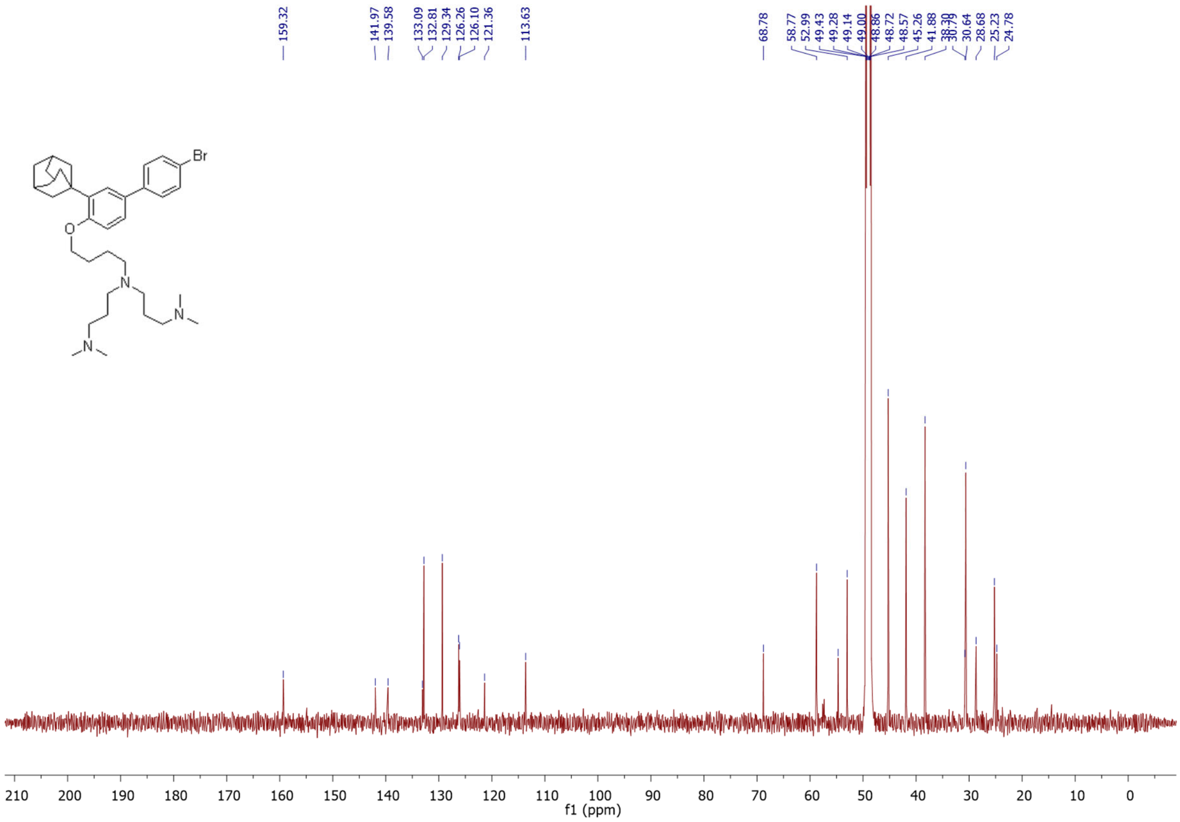

^{1}H-NMR (600 MHz, $CDCl_3$) compound **24**.

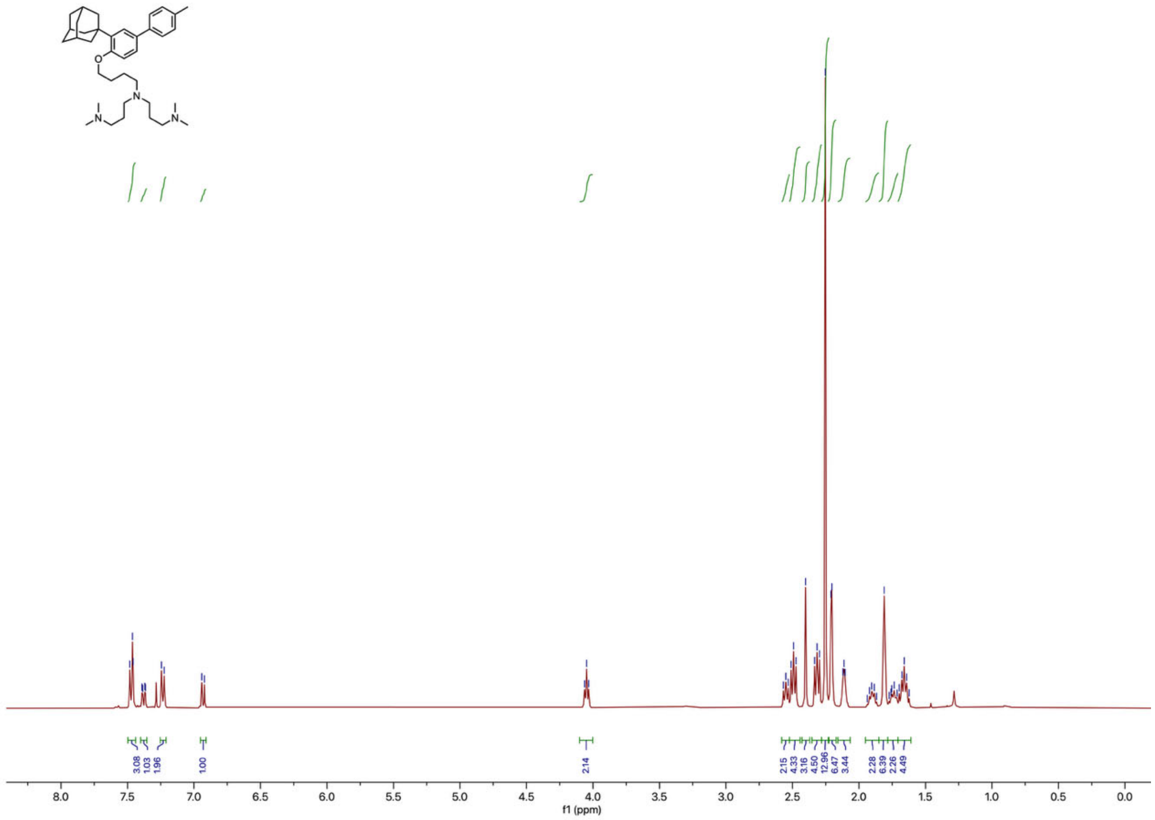

^{13}C-NMR (100 MHz, $CDCl_3$) compound **24**.

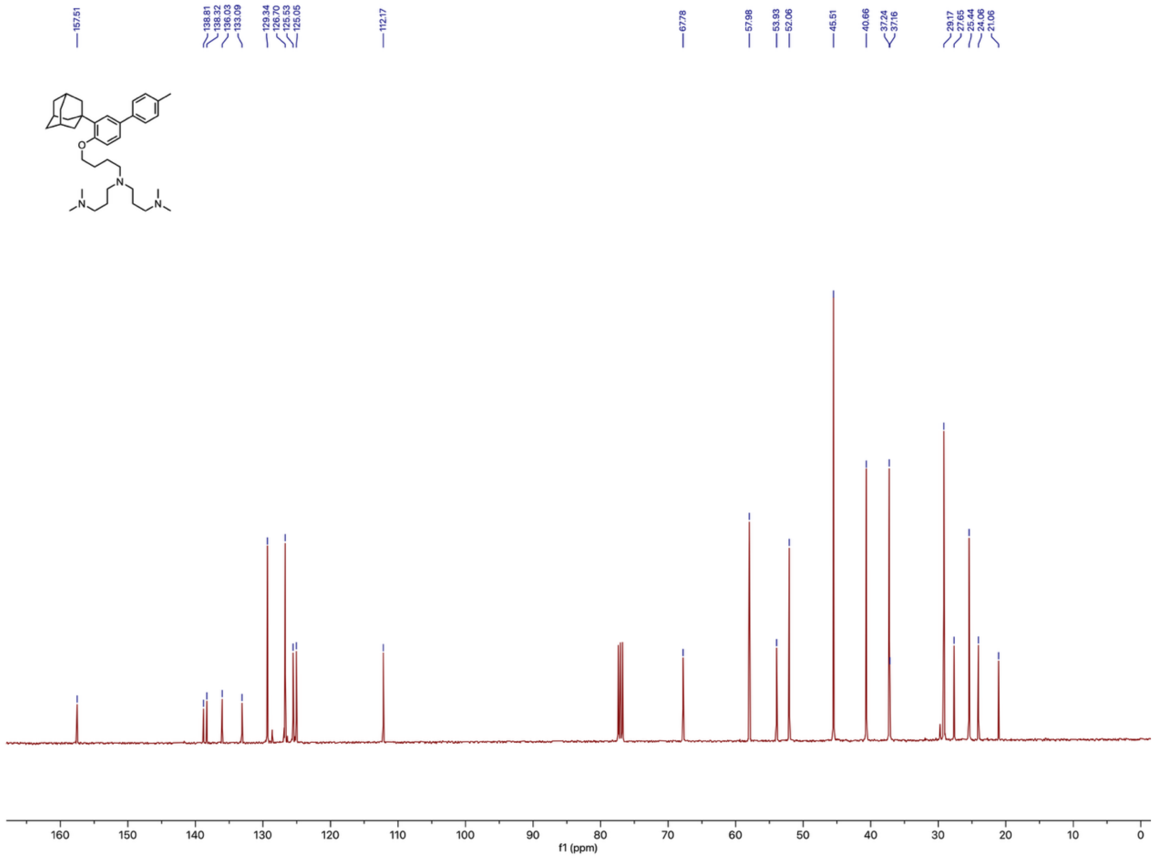

^{1}H-NMR (400 MHz, $CDCl_3$) compound **25**.

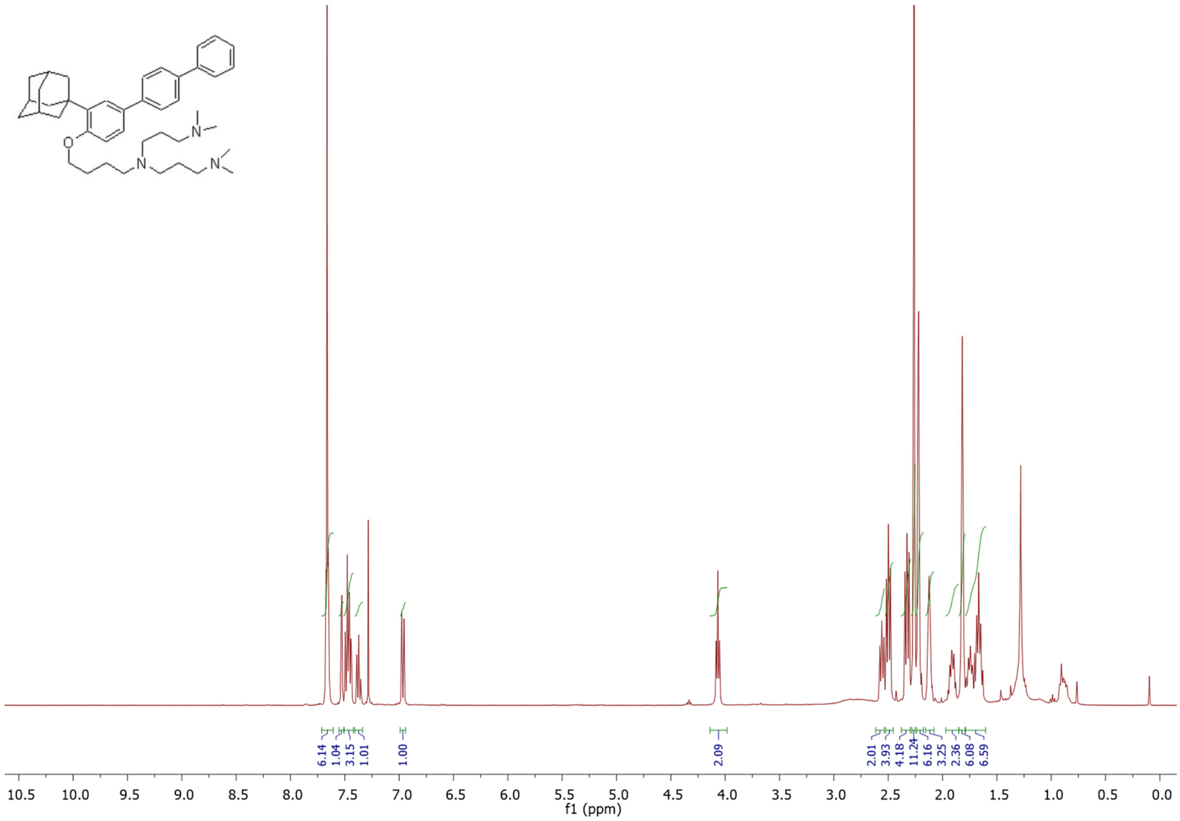

^{13}C-NMR (100 MHz, $CDCl_3$) compound **25**.

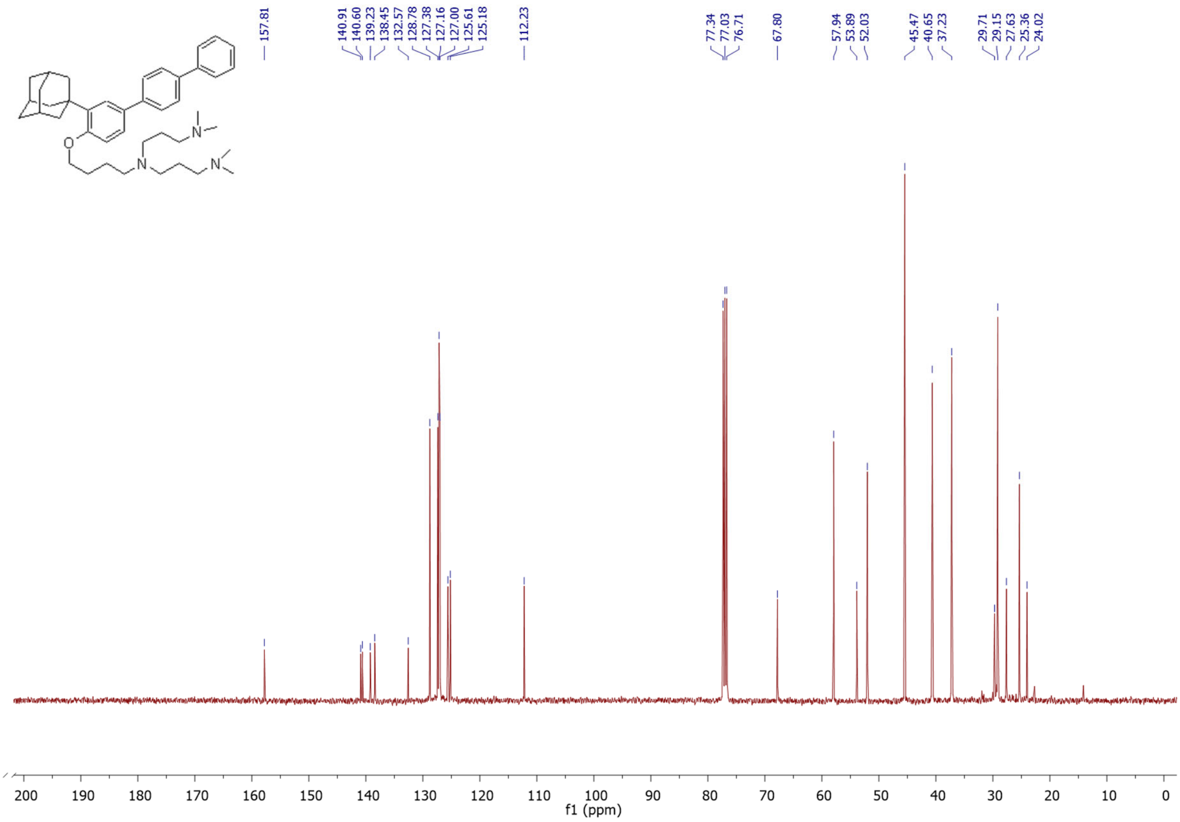

^{1}H-NMR (600 MHz, $CDCl_3$) compound **26**.

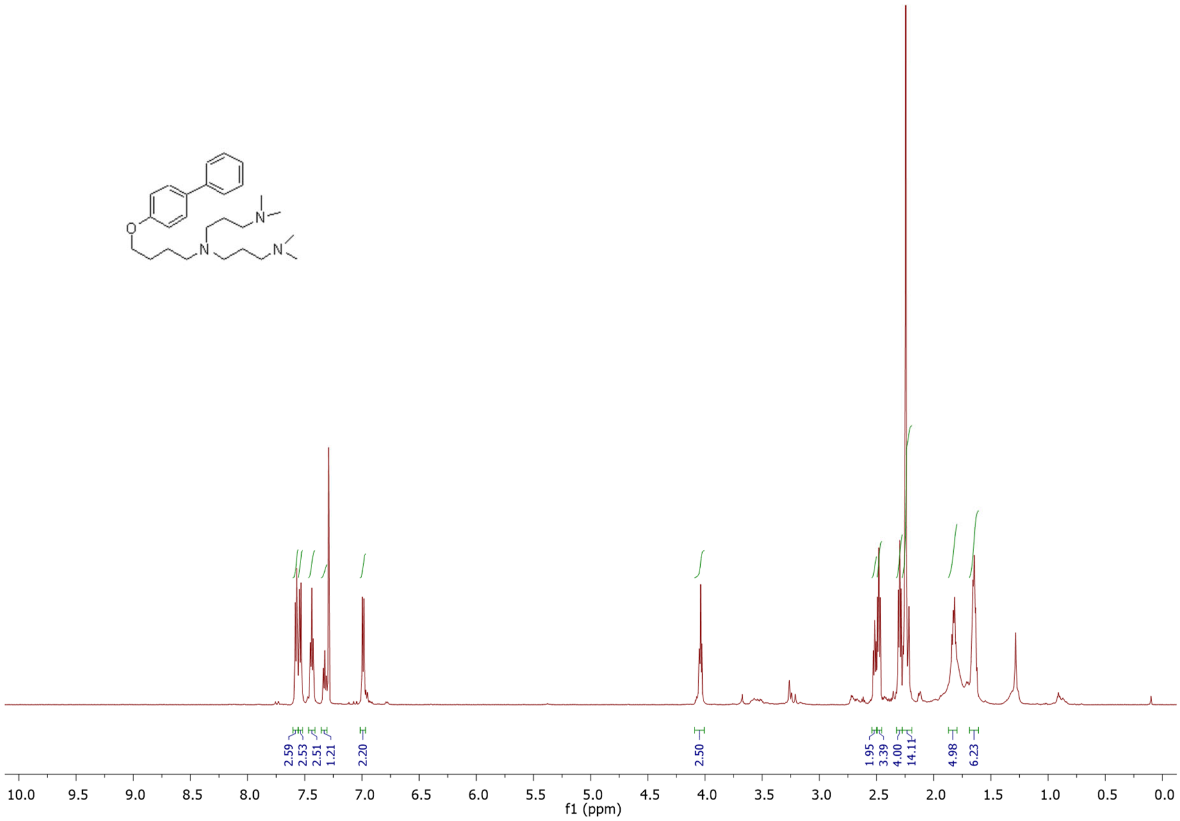

^{13}C-NMR (150 MHz, $CDCl_3$) compound **26**.

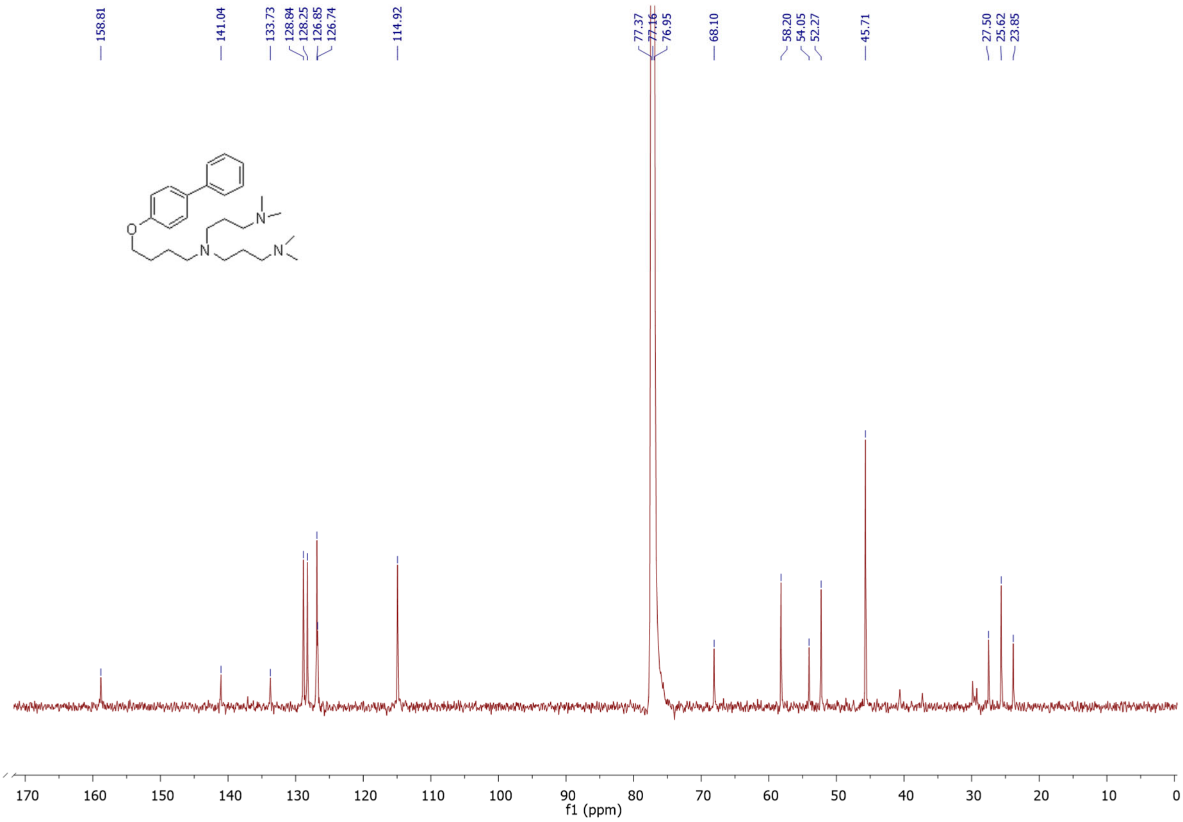

^{1}H-NMR (400 MHz, $CDCl_3$) compound **27**.

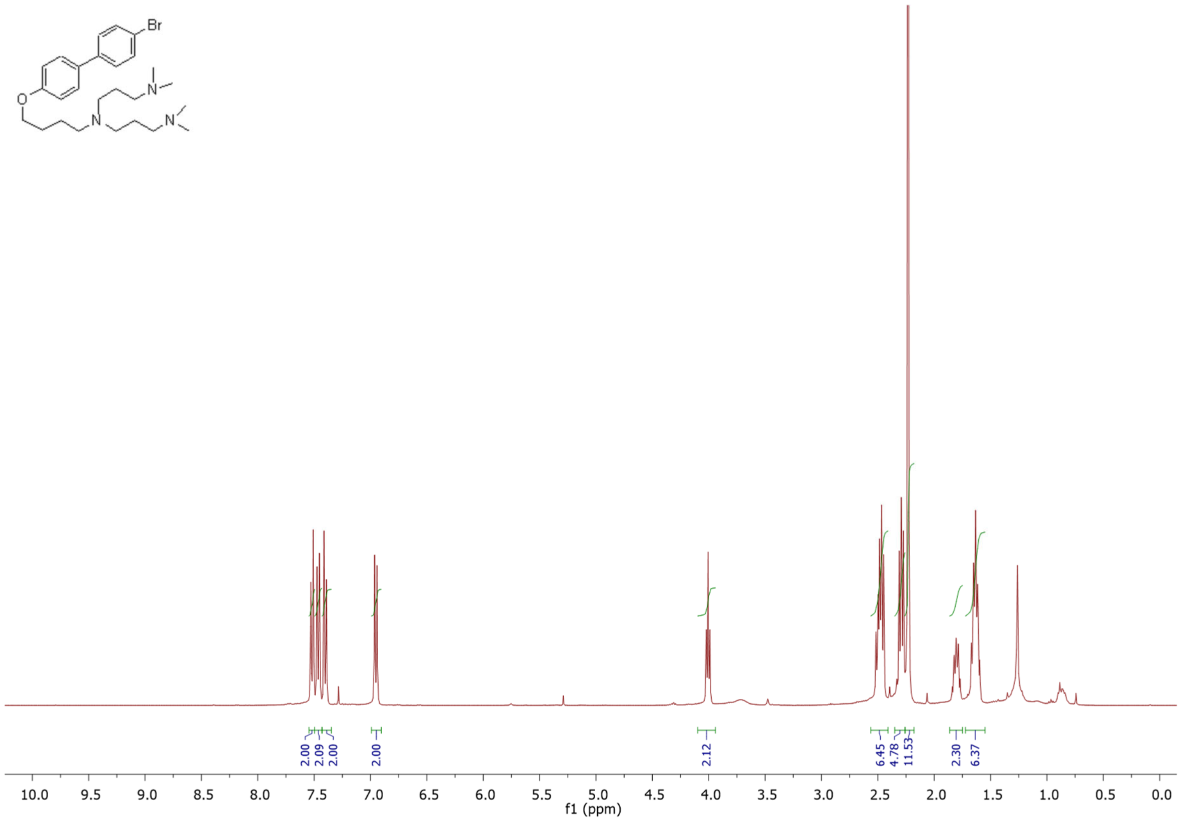

^{13}C-NMR (100 MHz, $CDCl_3$) compound **29**.

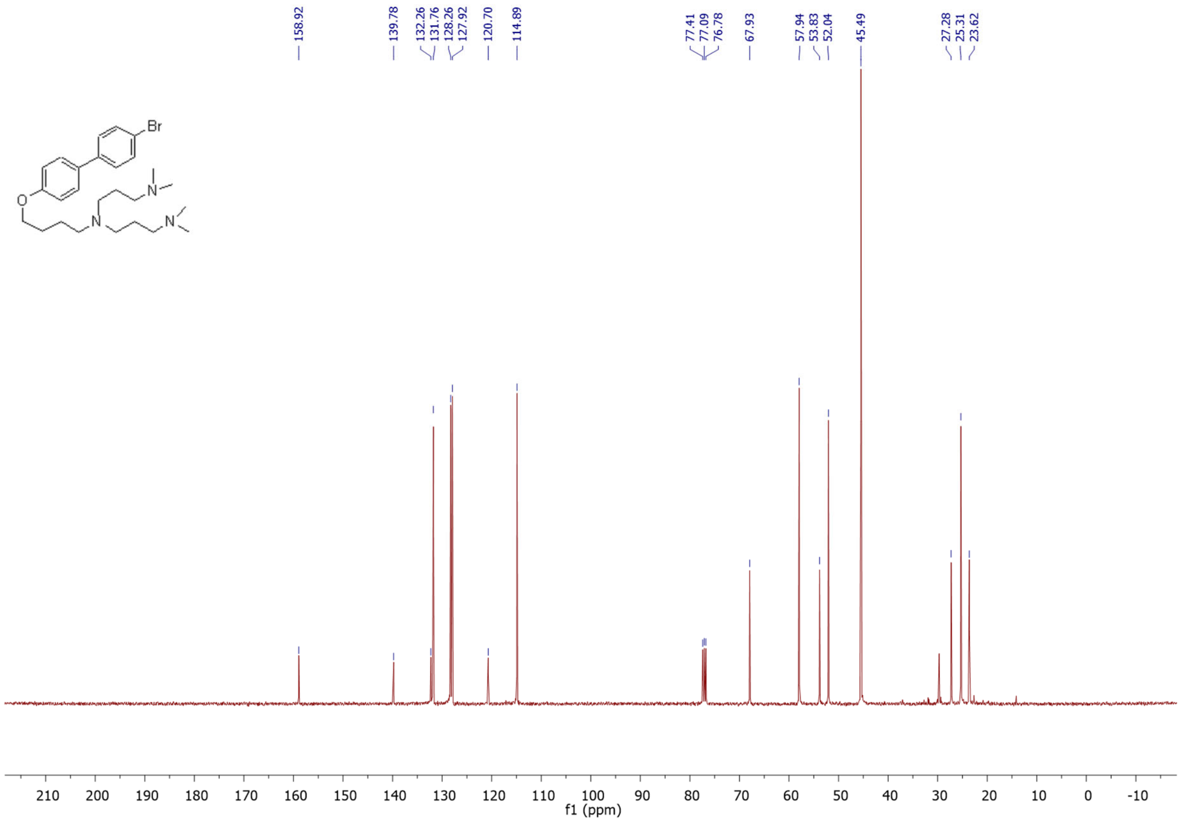

^{1}H-NMR (600 MHz, $CDCl_3$) compound **28**.

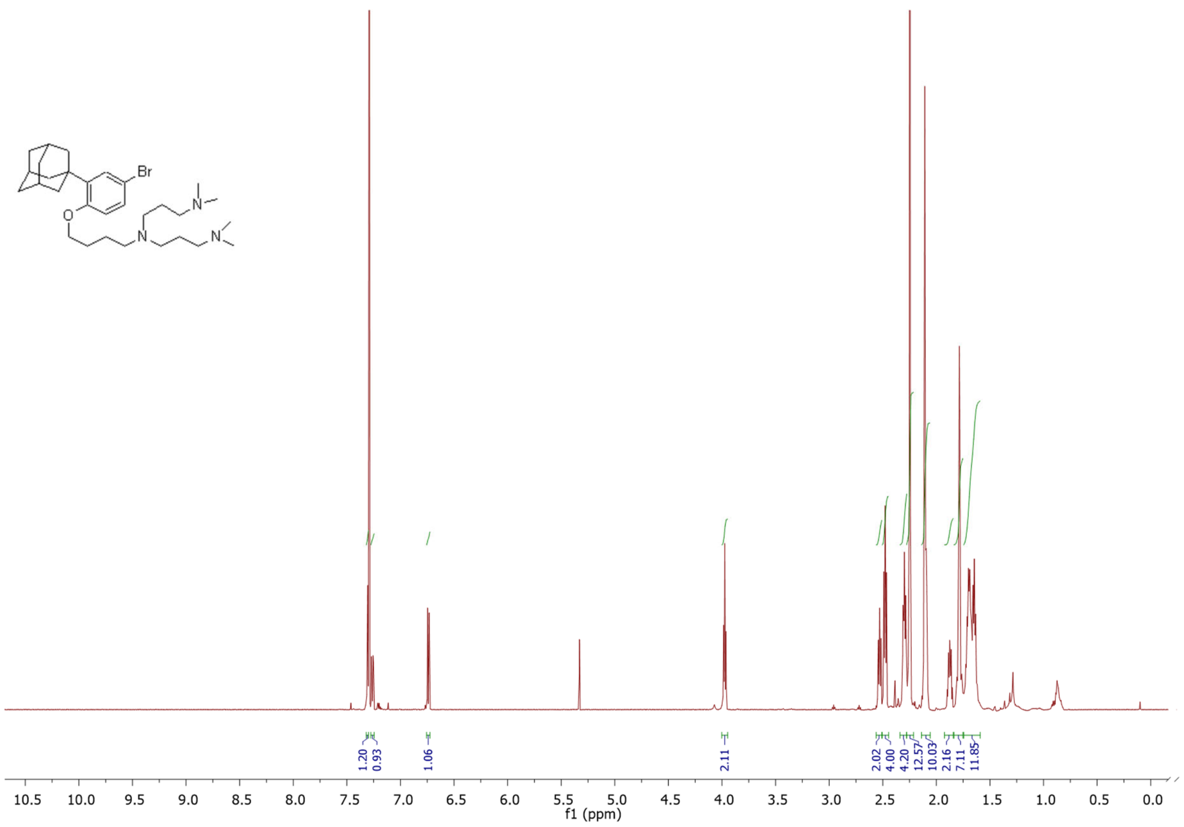

^{13}C-NMR (150 MHz, $CDCl_3$) compound **28**.

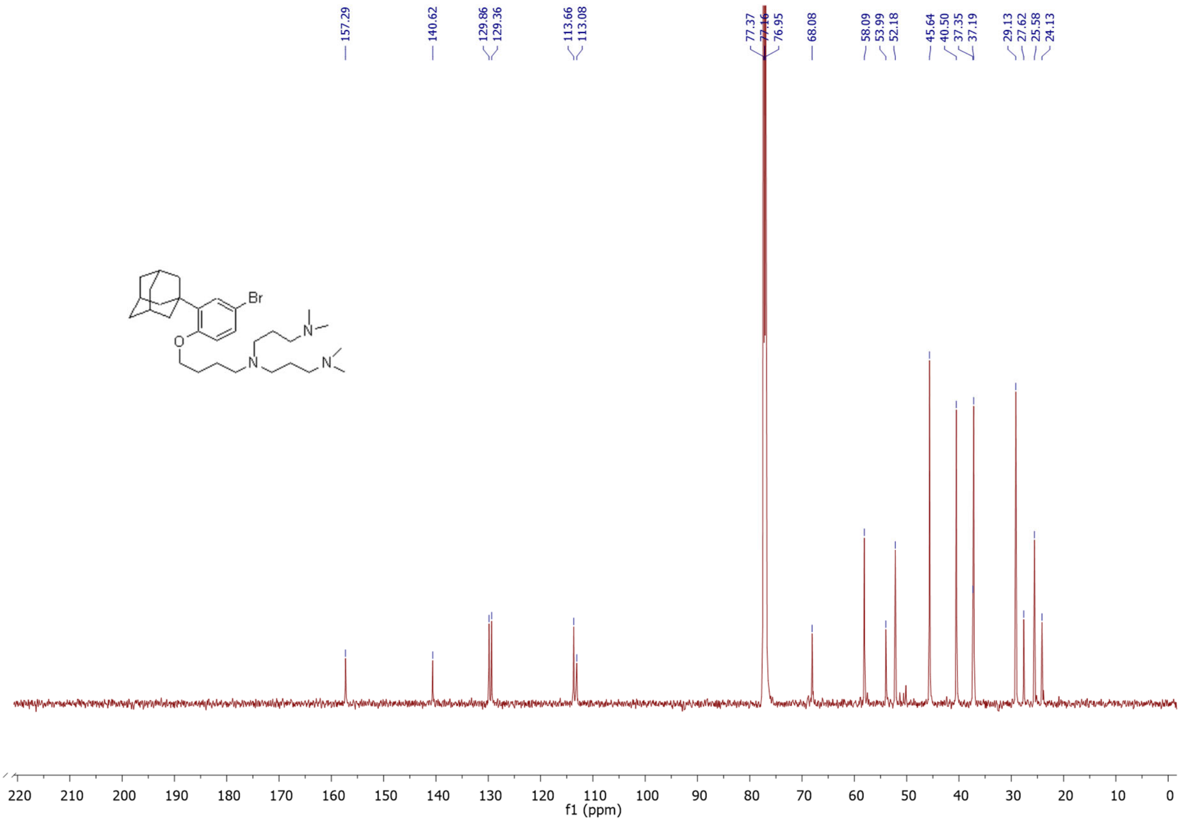

Supplement: Supplementary file 1 [file antibiotics-14-00956-s001.zip › antibiotics-3810612-supplementary.pdf]
